# M1 macrophage-derived extracellular vesicle containing tsRNA-5006c promotes osteogenic differentiation of aortic valve interstitial cells through regulating mitophagy

**DOI:** 10.7717/peerj.14307

**Published:** 2022-12-02

**Authors:** Hao Xia, Mingjian Gao, Jun Chen, Guanshen Huang, Xiuting Xiang, Yuyan Wang, Zhaohui Huang, Yongchun Li, Shuang Su, Zewei Zhao, Qingchun Zeng, Yunjun Ruan

**Affiliations:** 1 Department of Geriatrics, Nanfang Hospital, Southern Medical University, Guangzhou, Guangdong, China; 2 Department of Cardiology, Southern University of Science and Technology Hospital, Shenzhen, Guangdong, China; 3 Department of traditional Chinese Medicine, Nanfang Hospital, Southern Medical University, Guangzhou, Guangdong, China

**Keywords:** Osteogenic differentiation, tRNA-derived small RNA, Extracellular vesicles, Macrophage M1 polarization, Mitophagy

## Abstract

**Background:**

Osteogenic differentiation of aortic valve interstitial cells (AVICs) plays a key role in the calcific aortic valve disease progression. Extracellular vesicles (EVs)-derived from M1-polarized macrophages (M1-EVs) orchestrated intercellular communication by delivering non-coding RNAs such as tRNA-derived small RNAs (tsRNAs) is crucial for cardiovascular disease. However, the role and mechanism of M1-EVs tsRNAs in osteogenic differentiation of AVICs remains largely unclear.

**Methods:**

M1-EVs and PBS treated-RAW 264.7 cell-derived EVs (NC-EVs) were incubated with AVICs and subjected to small RNA sequencing. Candidate tsRNA in M1-EVs was silenced to explore their effects on AVIC osteogenic differentiation and mitophagy.

**Results:**

DiI-labeled M1-EVs were internalized by AVICs, resulting in significantly increased calcium nodule formation and expression of osteogenesis-related genes in AVICs, including RUNX2, BMP2, osteopontin, and SPP1, compared with NC-EVs. Small RNA sequencing revealed that 17 tsRNAs were significantly up-regulated such as tsRNA-5006c, while 28 tsRNAs were significantly down-regulated in M1-EVs compared with NC-EVs. Intriguingly, tsRNA-5006c-deleted M1-EVs treatment significantly reduced calcium nodule formation and expression of osteogenesis-related genes in AVICs relative to control group. Moreover, target genes of tsRNA-5006c were mainly involved in autophagy-related signaling pathways, such as MAPK, Ras, Wnt, and Hippo signaling pathway. Hallmarks of mitophagy activation in AVICs including mitophagosome formation, TMRM fluorescence, expression of LC3-II, BINP3, and PGC1α, were significantly elevated in the M1-EVs group compared with NC-EVs group, whereas M1-EVs tsRNA-5006c inhibitor led to a significant reduction in these indicators.

**Conclusion:**

M1-EVs carried tsRNA-5006c regulates AVIC osteogenic differentiation from the perspective of mitophagy, and we provide a new target for the prevention and treatment of aortic valve calcification.

## Introduction

Calcific aortic valve disease (CAVD) is a degenerative disease and is a major cause of aortic valve stenosis and has become the third largest cardiovascular disease in developed countries. The prevalence of CAVD increases dramatically with age, with a prevalence of >25% in people over 65 years and as high as 50% in people over 85 years (Kraler et al., 2022). CAVD is mainly presented by progressive fibro-calcific remodeling of the aortic valve leaflets that results in hemodynamic disturbances, cardiomyocyte apoptosis, necrosis, fibrosis change, *etc.*, and ultimately premature death (Shen et al., 2018). During the pathological process of CAVD, aortic valve interstitial cells (AVICs) undergo osteogenic reprogramming, which is mainly manifested as differentiation from quiescent fibroblast-like phenotype to osteoblast-like and fibrotic phenotype, which is accompanied by calcium deposition (Mkannez et al., 2018). Understanding the mechanism of the loss of the original phenotype and how to acquire the osteoblastic phenotype of AVIC will be beneficial to the development of targeted therapeutic strategies for ectopic calcification and provide a theoretical basis for the treatment of CAVD.

Increasing studies have observed the presence of macrophage infiltration in the heart valves of CAVD patients, and abnormal activation of macrophages is considered to be one of the key mechanisms of CAVD (Wada et al., 2013; Zhou et al., 2020). For example, recruitment of macrophages increased in a CAVD model, and macrophages interacted with AVICs led to promoting osteogenic differentiation but not dystrophic calcification and reducing alternative splicing of STAT3 β (Driscoll, Cruz & Butcher, 2021). As is well known, macrophages are mainly clustered into two polarized types M1 and M2 macrophages. Intriguingly, studies have shown that macrophage M1 polarization is the main phenotype that contributes to the progression of osteogenic differentiation AVICs of in CAVD. For instance, surface markers of macrophages were significantly enhanced in calcified aortic valve lobules compared to normal lobules, and infiltrating macrophage was dominated by the M1 macrophage subsets, and conditioned medium from M1 phenotype macrophages significantly enhanced osteogenic differentiation of AVIC cells (Li et al., 2017). Li et al. (2016) also proved that M1 macrophages facilitate osteogenic differentiation of AVICs. However, how M1 macrophages mediate AVIC osteogenic differentiation in CAVD progression remains unclear.

Extracellular vesicles (EVs) can act as intercellular messengers for macrophages to crosstalk other cells and cause phenotype changes in recipient cells (Bakhshian Nik, Hutcheson & Aikawa, 2017; Blaser & Aikawa, 2018) though transporting cargo (DNA, RNA, lipid, metabolites, protein, *etc.*) (Kalluri & LeBleu, 2020). Hutcheson and his colleagues deem that macrophages not only play an indirect role in promoting calcification remodeling but also directly promote cardiovascular calcification by releasing calcified EVs (Hutcheson, Blaser & Aikawa, 2017). New et al. (2013) also suggested that macrophage-derived matrix vesicles have high calcification and aggregation potential due to containing S100A9 (pro-inflammatory and pro-thrombotic factor) and annexin V. Therefore, we speculate that macrophage-derived EVs may be the key to macrophage-induced calcium deposition and osteogenic differentiation of AVIC.

tRNA-derived small RNA (tsRNAs) is a novel type of noncoding RNA cleavage from tRNA. tsRNAs are roughly divided into two categories according to their location on the tRNA: tRNA-derived fragments (tRF) and tRNA halves (tiRNA) (Guzzi & Bellodi, 2020). In the past, tsRNAs were considered to be random degradation products of tRNAs thereby without attracting much attention. However, in recent years, numerous studies have indicated that tsRNAs occur in a specific biogenesis pattern and play a non-negligible role in the progression of various diseases, such as tumors (Wen et al., 2021), reproductive-related diseases(Li et al., 2021), and cardiovascular diseases (Cao, Cowan & Wang, 2020). Importantly, many studies have shown that cardiac pathological conditions can induce abnormal expression of tsRNAs in various cardiovascular diseases, such as rheumatoid heart disease with atrial fibrillation (Yang et al., 2021), myocardial ischemia (Liu et al., 2020), and atherosclerosis (He et al., 2021; Wang et al., 2021). However, whether tsRNAs can mediate the involvement of macrophages in CAVD progression has not yet been reported.

In this study, we first obtained M1-polarized macrophage-derived EVs and then evaluated the effect of EVs on osteogenic differentiation. Next, the tsRNA expression profiles of EVs were characterized to screen out candidate tsRNAs. Inhibitor of candidate tsRNA was used to silence tsRNA expression in EVs, and the effects of tsRNA-deleted EVs on osteogenic differentiation and mitophagy of AVICs were explored. Our study will explore the molecular mechanism of AVICs calcification induced by M1 macrophages through EVs tsRNA crosstalk from the perspective of mitophagy.

## Material and Methods

### Cell culture and transfection

Mouse mononuclear macrophage leukemia cell line RAW 264.7 was purchased from Procell (CL-0190). Dulbecco’s modified Eagle’s medium (10-013-CVRC, Corning, NY, USA) contained 10% fetal bovine serum and 1% Penicillin-Streptomycin Solution (E607011; Sangon, China) was used to culture RAW 264.7 cells in a humidified atmosphere of 95% air and 5% CO_2_ at 37 °C. To obtain M1-polarized macrophages, RAW 264.7 cells were stimulated with 10 pg/mL lipopolysaccharide (LPS) and 20 ng/mL IFN-γ for 24 h.

Human AVICs were cultured in iCell Primary Mesenchymal Cell Culture System (PriMed-iCell-025; iCell Bioscience Inc., Shanghai, China) in a humidified atmosphere of 95% air and 5% CO_2_ at 37 °C. For osteogenic differentiation induction, AVICs were cultured in an osteogenic differentiation medium (PH-B002; PH Biomedicine) for 7 days at 37 °C.

To silencing of tsRNA-5006c, the tsRNA-5006c inhibitor (Table S1) purchased from GenePharm (China) was transfected into M1-polarized macrophages using Lipofectamine™ 2000 (Invitrogen, USA). A nonsense sequence was used as the inhibitor negative control (NC). The day before transfection, M1-polarized macrophages were seeded in a six-well plate with 3 ×10^5^ cells/well. When the confluence reached 80%–90%, transfection was carried out. Taken 5 µL each of tsRNA-5006c inhibitor and NC and diluted in 45 µL OPTI-MEM (Corning), and then mixed with Lipofectamine™ 2000 regent for 20 min at 25 °C. After that, the mixture was added to cell samples for another 24 h culture.

### Cell immunofluorescence (IF)

After the induction of M1 polarization in RAW 264.7 cells, the expression of M1 marker CD63 was detected by IF. Briefly, cells were fixed with 4% paraformaldehyde for 30 min at 25 °C and then permeabilized by 0.5% TritonX100 for 15 min at 25 °C. After washing three times with PBS, cells were incubated in a blocking solution of 3% BSA (diluted in PBS) for 30 min at 25 °C. Next, cells were incubated with pre-cooled anti-CD63 (1:200, ab239075; Abcam) at 4 °C overnight followed by incubation with goat anti-rat Alexa Flour 488 (1:300, ab150077; Abcam). Finally, cells were stained with DAPI (C1006; Beyotime, Shanghai, China) for 10 min in the dark condition and observed the fluorescence on a fluorescence microscope.

### RNA isolation

Total RNA was isolated from M1-polarized macrophage cells and EVs using the TRIzol reagent (Life Technologies). The concentration and purity of total RNA were assessed by spectrophotometer. The integrity of total RNA was assessed by agarose gel electrophoresis. Qualified total RNA was stored at 80 °C and used for subsequently RT-qPCR analysis and small RNA sequencing.

### RT-qPCR analysis

For mRNA expression, RNA was reverse-transcribed into cDNA by the RevertAid™ First Strand cDNA Synthesis Kit, with DNase I (K16225; Thermo Fisher, Waltham, MA, US), using random primers. For tsRNAs expression, RNA was pretreated with aRevertAid™ First Strand cDNA Synthesis Kit, with DNase I (K16225; Thermo Fisher, Waltham, MA, USA) using stem-loop reverse transcription primers. Next, the amplification reaction was carried out with cDNA as the template using the 2PCR Master Mix (Roche, Basel, Switzerland) on the ABI Q6 Flex Real-time PCR system (Applied Biosystems, Waltham, MA, USA). All primer’s sequences were synthesized by GenePharm (China) and shown in Table S1. The 2^−ΔΔCT^ method was used for normalizing gene expression relative to GAPDH or U6.

### ELISA assays

The production of IL-6, TNF-α, IL-1β, IL-10, IL-12, and IL-13 were detected by using Mouse IL-6 ELISA Kit (ab222503; Abcam), Mouse TNF alpha ELISA Kit (ab208348, Abcam), Mouse IL-1 beta ELISA Kit (ab197742, Abcam), Mouse IL-10 ELISA Kit (ab255729, Abcam), Mouse IL-12(p70) ELISA Kit (EK0422; ScienCell™), Mouse IL-13 ELISA Kit (BMS6015, invitrogen), respectively, according to the manufacturer’s instruction.

### Isolation of EVs

EVs were isolated from M1-polarized macrophages (M1-EVs) and PBS treated-RAW 264.7 cells (NC-EVs) using ultracentrifugation methods. Briefly, cells were cultured in a 10 cm dish with DMEM containing 10% FBS and 1% P/S and maintained in a humidified atmosphere of 95% air and 5% CO_2_ at 37 °C. The culture medium was replaced by a serum-free exosome-depleted medium (10 mL) when cell growth had reached the logarithmic phase (1 ×10^7^/dish). After culture for another 48 h, the medium was sequentially centrifuged at 500 g for 5 min, 2,000 g for 5 min, and 10,000 g for 30 min at 4 °C to remove the debris. Then, the supernatant was filtered through a 0.22-µm filter tube and centrifuged at 120,000 g for 2 h at 4 °C and the pellet was resuspended in PBS. Finally, crude EVs were centrifuged again at 120,000 g for 2 h at 4 °C to discard the contaminating protein to harvest purified EVs.

### Identification of EVs

The size and morphology of EVs were determined by transmission electron microscopy (TEM) and the distribution and concentration of nanoparticles were accessed by nanoparticle tracking analysis (NTA; ZetaView), according to the manufacturer’s instruction. The presence of M1 marker of CD63 was detected by western blot.

### Western blot

Total protein was extracted from EVs, AVICs, and arterial tissues using RIPA reagent, and the protein concentration was analyzed by a BCA kit. Then, approximately 20 µg protein sample was separated on 10% SDS-PAGE and transferred onto PVDF membrane. After that, membranes were blocked with 5% skimmed milk in TBST for 1 h at 25 °C followed by incubated with the primary antibodies at 4 °C overnight and incubated with secondary antibodies of Goat Anti-Mouse IgG H&L(HRP) (1:1000, ab205719; Abcam) or Goat Anti-Rabbit IgG H&L (HRP) (1:20000, ab6721; Abcam) for 1 h at 25 °C. Primary antibodies included CD63 (1:10000, ab216130; Abcam), BNIP3 (1:5000, ab109362; Abcam), PGC1 α (1:1000, ab106814; Abcam), LC3-II (1:1000, 3868Y; CST), BMP2 (1:1000, ab214821; Abcam), RUNX2 (1:200, sc-390715; Santa Cruz Biotechnology), osteopontin (1:1000, 25715-1-AP; Proteintech), GAPDH (1:2000, 60004-1-Lg; Proteintech). Finally, membranes were exposed to ECL and imaged by Chemiluminometer (Clinx Science Instruments, Shanghai, China).

### EVs uptake experiment

The cell membrane red fluorescent probe DiI (C1036; Beyotime, Shanghai, China) was used to label EVs. Briefly, EVs were co-cultured with DiI dye solution for 10 min at 25 °C and then filtered away the unbound dye. The DiI-labeled EVs were co-cultured with AVICs in iCell medium for 24 h. To investigate the EVs internalization, AVICs underwent routine fixation, permeabilization, and DAPI nuclear staining as described in IF after co-culture. Finally, EVs internalization was visualized by a fluorescence microscope.

### Alizarin red staining

The calcium deposit in AVICs was evaluated by Alizarin red staining. Briefly, AVICs were fixed in 4% paraformaldehyde for 15 min and then stained with Alizarin red solution for 30 min. After washing three times with PBS to remove the free dye, added deionized-water to protect cells from drying. Finally, AVICs were observed and imaged using a microscope.

### Small RNA sequencing

RNA extracted from M1-EVs (*n* = 3) and NC-EVs (*n* = 3) were used for small RNA sequencing. The Multiplex Small RNA Library Prep Kit was used to prepare Small RNA libraries. Briefly, a 3′ adaptor was ligated to RNA and, then hybridization of reverse transcription primers followed by ligated with a 5′adaptor. Next, RNA was reverse transcribed into first-strand cDNA with bidirectional adaptor sequences as the template. After PCR amplification, size within 135-170 bp fragment was separated from 8% gel electrophoresis and purified. The PCR-amplified libraries were accessed by a Agilent 2100 BioAnalyzer. Finally, small RNA sequencing was carried out in a single-end 150 bp run using the HiSeq2500 platform (Illumina, USA). The statistical power of this experimental design, calculated in RNASeqPower, is 0.115.

Raw data were filtered through Fast-QC software to obtain clean data. Then, clean reads were mapped onto miRBase and piRNAcluster databases to exclude contamination and interferences from the miRNA and piRNA. The remaining clean reads were mapped onto tRFdb and tRFMINTbase database to obtain tsRNA, and the read counts of tsRNA were considered as expression level. The differentially expressed tsRNAs (DEtsRNAs) was identified under the condition of log2 fold change >1 or <−1, *P* value <0.05 using DESeq package. All the DEtsRNAs were subjected to GO and KEGG analysis.

### Mitophagy observation

Lysosomes and mitochondria were labeled with the Lyso-Tracker Green (C1048; Beyotime, Shanghai, China) and Mito-Tracker Red (C1048; Beyotime, Shanghai, China) probes, respectively, and the co-localization of lysosomes and mitochondria was visualized to characterize mitophagy flux. Briefly, Lyso-Tracker Red solution was diluted into a final concentration of 50 nM using medium (1:13,333). Mito-Tracker Red solution was diluted into a final concentration of 20 nM using a medium (1:5000). Next, cells were incubated with Lyso-Tracker Red for 5 min at 37 °C followed by incubation with Mito-Tracker Red for 15 min at 37 °C. Finally, cells were observed with a fluorescence microscope.

### TMRM staining analyzed by flow cytometry

Mitochondrial membrane potential (MMP) of AVICs was measured by flow cytometry using TMRM staining. AVICs were harvested and made into a single cell suspension of density 2∼5 ×10^5^ cells/mL. Added 50 nM of prepared TMRM Perchlorate staining solution to each tube of cell samples, mixed and incubated at 37 °C for 60 min in the dark. After centrifugation, the supernatant was removed and the cell pellet was washed with PBS. Finally, the AVICs were subjected to a flow cytometer to detect the fluorescence of TMRM.

### Statistical analysis

One-way analysis of variance (ANOVA) with Tukey pos *t*-test was used to assess the difference between three groups, *t*-test was used to assess the difference between two groups. The significance threshold was *P* value less than 0.05. Data analysis was performed with GraphPad Prism 9.0 (GraphPad, San Diego, CA, USA).

## Results

### Identification of EVs derived from M1 macrophages

**Figure 1:**
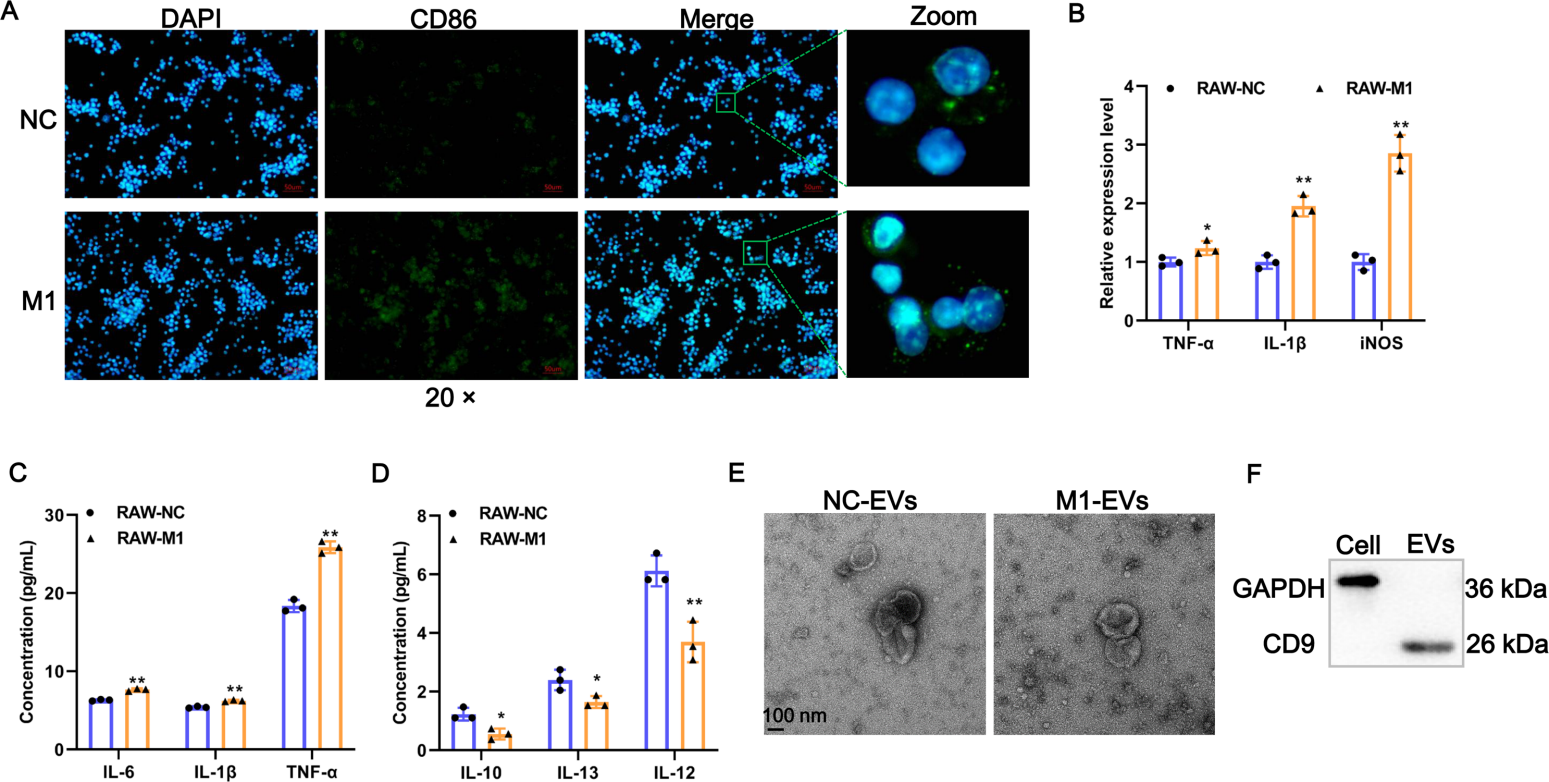
Identification of EV-derived from M1 macrophages. (A) Representative image of the immunofluorescence of CD86 in RAW 264.7 macrophage induced by LPS/IFN-γ (RAW-M1 group) or PBS (RAW-NC group). Scale bar: 50 µm. (B) The expression of TNF-α, IL-1β, and iNOS of M1 polarization markers was detected by RT-qPCR. (C) The concentration of TNF- α, IL-1β, and IL-6 of M1 polarization markers was detected by ELISA assays. (D) The concentration of IL-10, IL-12, and IL-13 of M1 polarization markers was detected by ELISA assays. (E) Representative image of EVs of TEM. EVs isolated from RAW 264.7 macrophage induced by LPS/IFN-γ (M1-EVs group) or PBS (NC-EVs group). (F) Surface marker of CD9 of EVs was detected by western blot. * *P* < 0.05, ** *P* < 0.01.

To investigate whether M1 macrophages can affect the phenotypic changes of AVICs, we first prepared M1-polarized macrophages. Results of IF assays showed that LPS/IFN-γ induction significantly provoked the expression of M1 polarization marker CD86 (Fig. 1A). The expression of TNF-α, IL-1β, and iNOS of M1 polarization markers were significantly increased after LPS/IFN-γ induction (Fig. 1B). Consistently, the concentration of pro-inflammatory cytokines of IL-6, TNF-α, and IL-1β in cell supernatant fluid were elevated following LPS/IFN-γ induction (Fig. 1C). On the contrary, the levels of M2-related anti-inflammatory cytokines of IL-10, IL-12, and IL-13 were significantly decreased after LPS/IFN-γ induction (Fig. 1D). Together, these results indicate that we successfully obtained M1-polarized macrophages. Next, EVs were isolated from M1-polarized macrophages (referred to as M1-EVs) or PBS treated-RAW 264.7 cells (referred to as NC-EVs) and characterized through TEM and western blot. These EVs were 50-200 nm and exhibited oval-shaped with a slight depression in the center (Fig. 1E) and readily expressed CD9 protein (Fig. 1F). Thus, M1-EVs and NC-EVs were harvested successfully.

### M1-EVs promote osteogenic differentiation of AVICs

**Figure 2:**
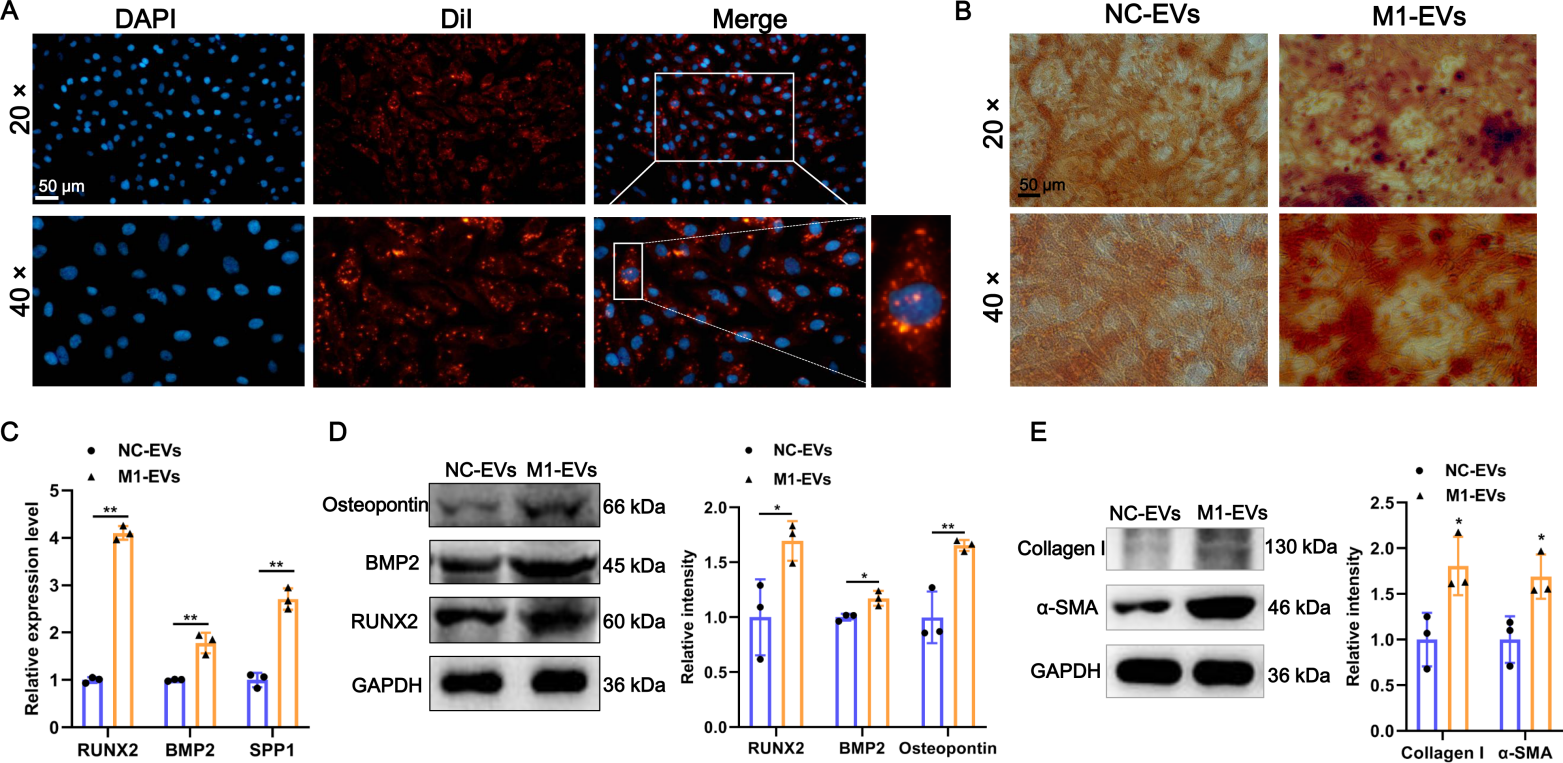
M1-EVs promote osteogenic differentiation of AVICs. (A) Representative image of DiI-labeled M1-EVs was internalized by AVICs. Scale bar: 50 µm. (B) Representative image of Alizarin red staining of AVICs pretreated with M1-EVs or NC- EVs. Scale bar: 50 µm. (C) The mRNA expression of RUNX2, BMP2, and SPP1 of osteogenesis-related genes in AVICs after incubated with M1-EVs was detected by RT-qPCR. (D) The protein expression of RUNX2, BMP2, and osteopontin of osteogenesis-related genes in AVICs after incubated with M1-EVs was detected by western blot. (E) The protein expression of α-SMA and Collagen I of fibrotic markers in AVICs after incubated with M1-EVs was detected by western blot. ns *P* > 0.05, * *P* < 0.05, ** *P* < 0.01.

Additionally, to investigate the function of M1-EVs on the AVICs, we first examined whether M1-EVs can be taken up into AVICs. As shown in Fig. 2A, after incubation with DiI-labeled M1-EVs, bright red fluorescent DiI internalization was present in the AVICs, demonstrating that M1-EVs were readily internalized into AVICs. Next, to explore the function of M1-EVs on the osteogenic differentiation of AVICs, we assessed the formation of calcium nodules and expression of osteogenesis-related genes. Alizarin red staining results revealed that the number of mineralized nodules was significantly elevated after M1-EVs incubation compared with NC-EVs (Fig. 2B). Similarly, M1-EVs incubation led to a significant up-regulation in the expression of osteogenesis-related genes RUNX2, BMP2, and SPP1 in both mRNA and protein level (Figs. 2C and 2D). Moreover, we also detected the effect of M1-EVs on collagen-related proteins in AVICs. The results showed that compared with the NC-EVs group, the expression of α-SMA and Collagen I in AVICs in the M1-EVs incubation group were significantly increased, suggesting that M1-EVs promote the fibrotic process of AVICs (Fig. 2E). Collectively, these results suggested that M1-EVs promote osteogenic differentiation of AVICs.

### tsRNA profile alters in M1-EVs

**Figure 3:**
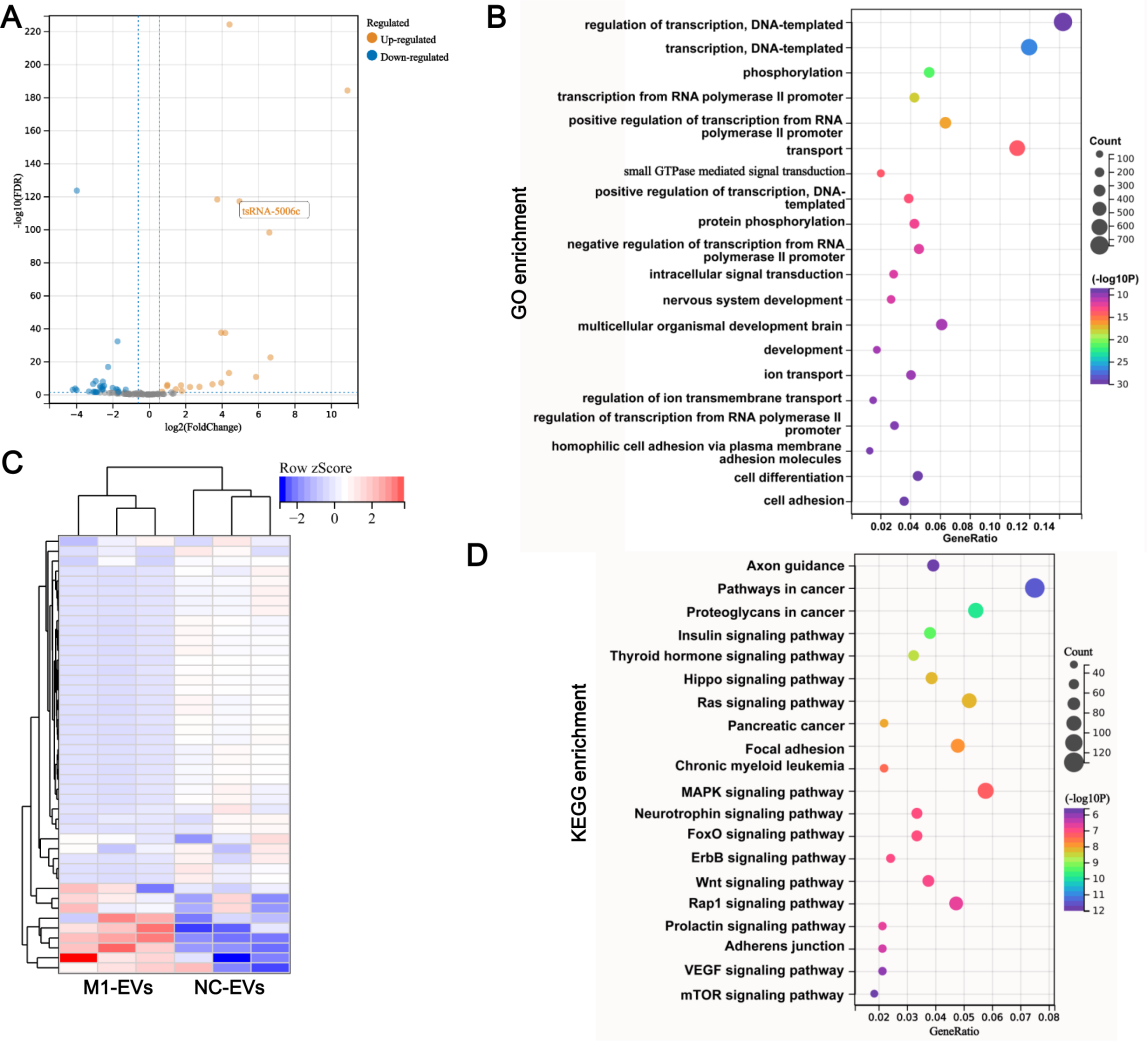
tsRNA profile alters in M1-EVs. Volcano plots (A) and heat map (B) are presenting the differentially expressed tsRNAs (DEtsRNAs). Red are up-regulated and green are down-regulated DEtsRNAs in the M1-EVs group compared with the NC- EVs group, respectively. (C) Top 20 of GO enrichment DEtsRNAs. (D) Top 20 of KEGG enrichment DEtsRNAs.

To determine whether EVs functional delivery by transporting the tsRNA, we subjected M1-EVs and NC-EVs to small RNA sequencing. RNA-seq revealed that 17 tsRNAs were significantly up-regulated such as tsRNA-5006c, while 28 tsRNAs were significantly down-regulated in M1-EVs compared with NC-EVs (Fig. 3A). These DEtsRNAs were separated into two categories and displayed in a heatmap (Fig. 3B). We predicted the target gene for DEtsRNAs and subjected it to the function and pathway enrichment analysis. Intriguingly, among the top 20 GO enrichment terms, seven terms related-transcriptional regulation were identified (Fig. 3C), suggesting that DEtsRNAs may mediate the transcriptional process of target genes. Target genes of DEtsRNAs were also enriched in cell differentiation of GO terms (Fig. 3C). Moreover, these DEtsRNAs were mainly involved in autophagy-related signaling pathways, such as MAPK, Ras, Wnt, mTOR, and the Hippo signaling pathway (Fig. 3D), suggesting that DEtsRNAs may change autophagy in recipient cells. Therefore, these M1-EVs DEtsRNAs may alter the phenotype of recipient cells through transcriptional regulation and autophagy-related pathways.

### tsRNA-5006c may be responsible for the M1-EVs effects

**Figure 4:**
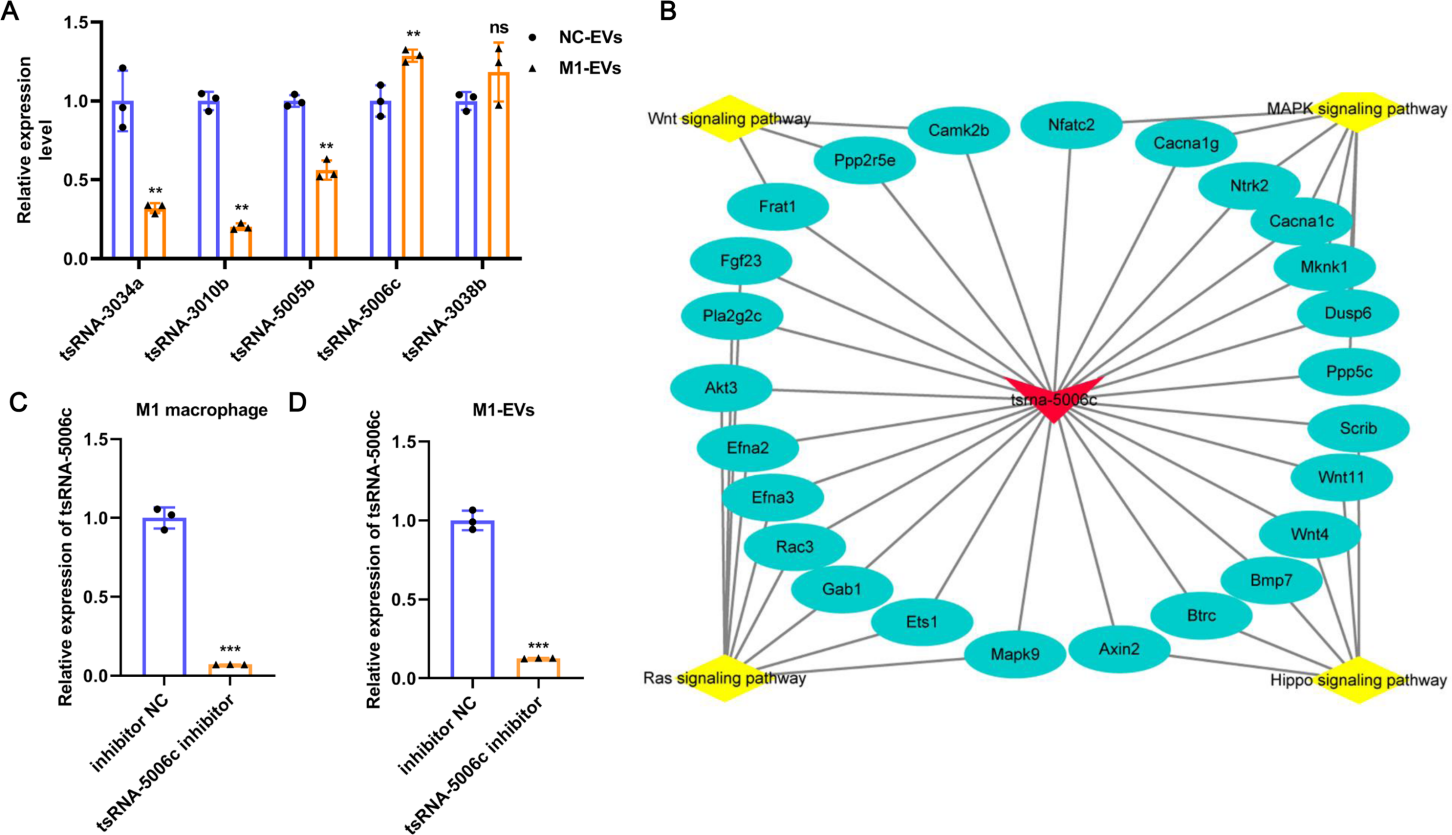
tsRNA-5006c may be responsible for the M1-EVs effects. (A) RT-qPCR was used to validate RNA sequencing results on the five DEtsRNAs. (B) The regulatory network of tsRNA-5006c _target genes_pathway. (C) The interference efficiency of tsRNA-5006c in M1 macrophages was detected by RT-qPCR. (D) The interference efficiency of tsRNA-5006c in M1-EVs was detected by RT-qPCR. ns *P* > 0.05, ** *P* < 0.01, *** *P* < 0.001.

To further verify the reliability of the sequencing results and screen out the key functional molecules in EVs, we selected five tsRNAs with high expression abundance and up-regulation in M1-EVs for RT-qPCR validation. As evident from Fig. 4A, compared with NC-EVs, only tsRNA-5006c expression was significantly up-regulated in M1-EVs, which was consistent with sequencing results. Accordingly, we focus on the tsRNA-5006c. According to MINTbase v2.0 (http://cm.jefferson.edu/MINTbase/), the tsRNA-5006c (5′-GCCCGGCTAGCTCAGTCGGTAGAGCATGGGAC-3′) also termed as tRF-32-PSQP4PW3FJIK1 and belonged to 5′-half tsRNA. The tsRNA-5006c is derived from the mature tRNA cleavage at the site of Lys-CTT. The regulatory network of tsRNA-5006c _target genes_pathway hinted at the underlying mechanisms of tsRNA-5006c function (Fig. 4B). For example, tsRNA-5006c could target Wnt4 and Btrc to involve in Hippo signaling pathway, target Ntrk2 and Mknk1 to involve in the MAPK signaling pathway, target Ppp2r5e and Camk2b to involve in the Wnt signaling pathway, and target Akt3 and Mapk9 to involve in Ras signaling pathway (Fig. 4B). Intriguingly, these four signaling pathways were associated with autophagy (Lorzadeh et al., 2021; Schmukler, Kloog & Pinkas-Kramarski, 2014; Wang et al., 2020; Zhou et al., 2015), which plays a crucial role in osteogenic differentiation (Zhou et al., 2021). Together, tsRNA-5006c may be responsible for the M1-EVs enhanced osteogenic differentiation of AVICs, through autophagy-related pathway.

### M1-EVs tsRNA-5006c inhibitor suppress osteogenic differentiation of AVICs

**Figure 5:**
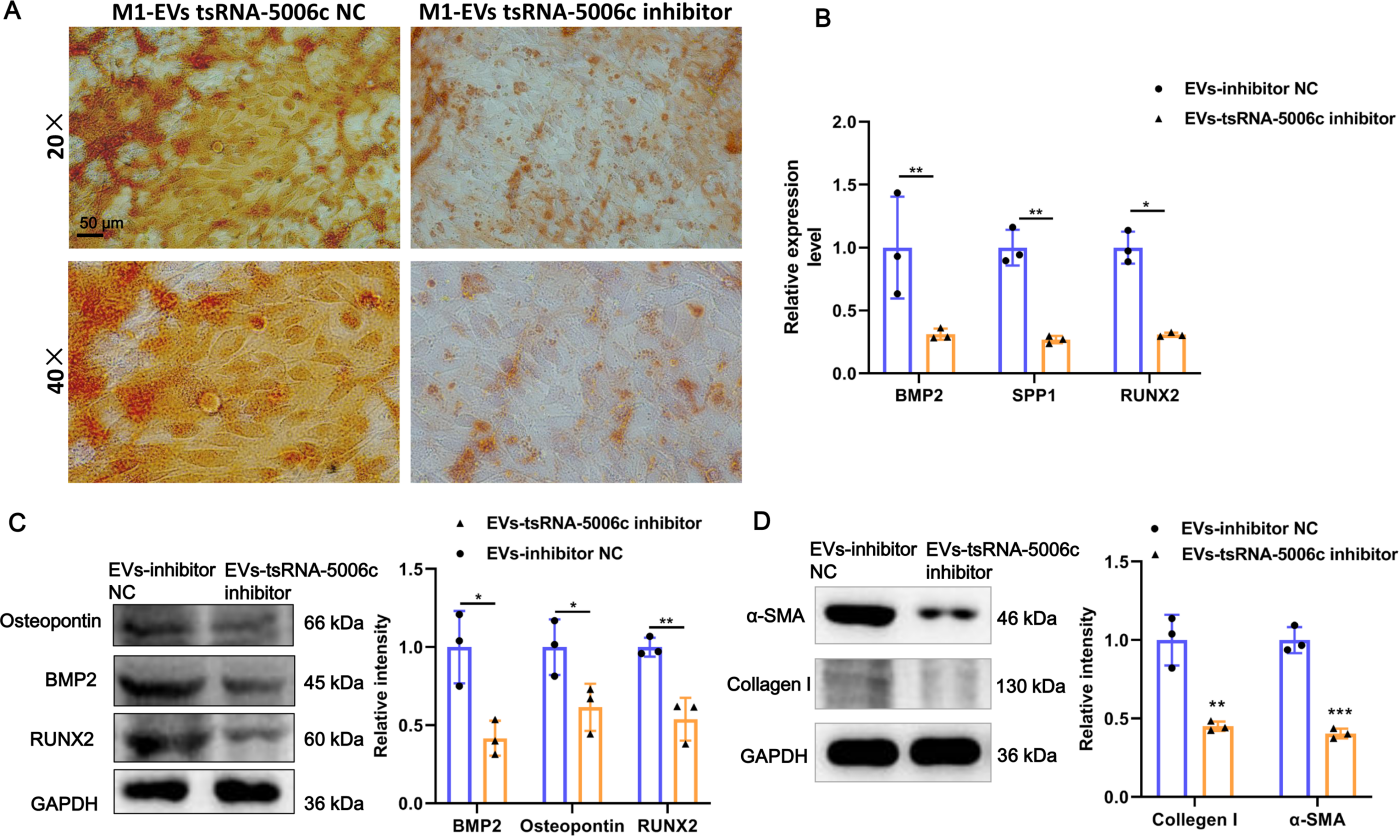
M1-EVs tsRNA-5006c inhibitor suppress osteogenic differentiation of AVICs. (A) Representative image of Alizarin red staining of AVICs pretreated with M1-EVs after tsRNA-5006c silencing. Scale bar: 50 µm. (B) The mRNA expression of RUNX2, BMP2, and SPP1 of osteogenesis-related genes in AVICs pretreated with M1-EVs after tsRNA-5006c silencing was detected by RT-qPCR. (C) The protein expression of RUNX2, BMP2, and osteopontin of osteogenesis-related genes in AVICs pretreated with M1-EVs after tsRNA-5006c silencing was detected by and western blot. (D) The protein expression of α-SMA and Collagen I of fibrotic markers in AVICs-pretreated with M1-EVs after tsRNA-5006c silencing was detected by western blot. NC is the negative control of tsRNA-5006c inhibitor. * *P* < 0.05, ** *P* < 0.01.

To interrogate the role of M1-EVs tsRNA-5006c in osteogenic differentiation of AVICs, we first treated M1 macrophages with tsRNA-5006c inhibitor and then harvested EVs. After tsRNA-5006c inhibitor administration, tsRNA-5006c expression was significantly reduced in both M1 macrophages and M1-EVs, compared to the tsRNA-5006c inhibitor NC treatment (Figs. 4C and 4D), suggesting that the interference efficiency of tsRNA-5006c was good. Then, tsRNA-5006c-deleted M1-EVs were co-cultured with AVICs. As the consequence of tsRNA-5006c interference, calcium nodule formation of AVICs was significantly diminished by Alizarin Red staining (Fig. 5A). The expression of osteogenesis-related genes RUNX2, BMP2, and SPP1 in recipient AVICs were significantly decreased in both mRNA and protein level upon tsRNA-5006c silencing (Figs. 5B and 5C). Also, the protein expression of fibrotic makers Collagen I and α-SMA were significantly decreased after tsRNA-5006c silencing (Fig. 5D). Therefore, our data suggest that M1-EVs promote osteogenic differentiation of AVICs by delivering tsRNA-5006c.

### M1-EVs tsRNA-5006c enhance osteogenic differentiation of AVICs through mitophagy

**Figure 6:**
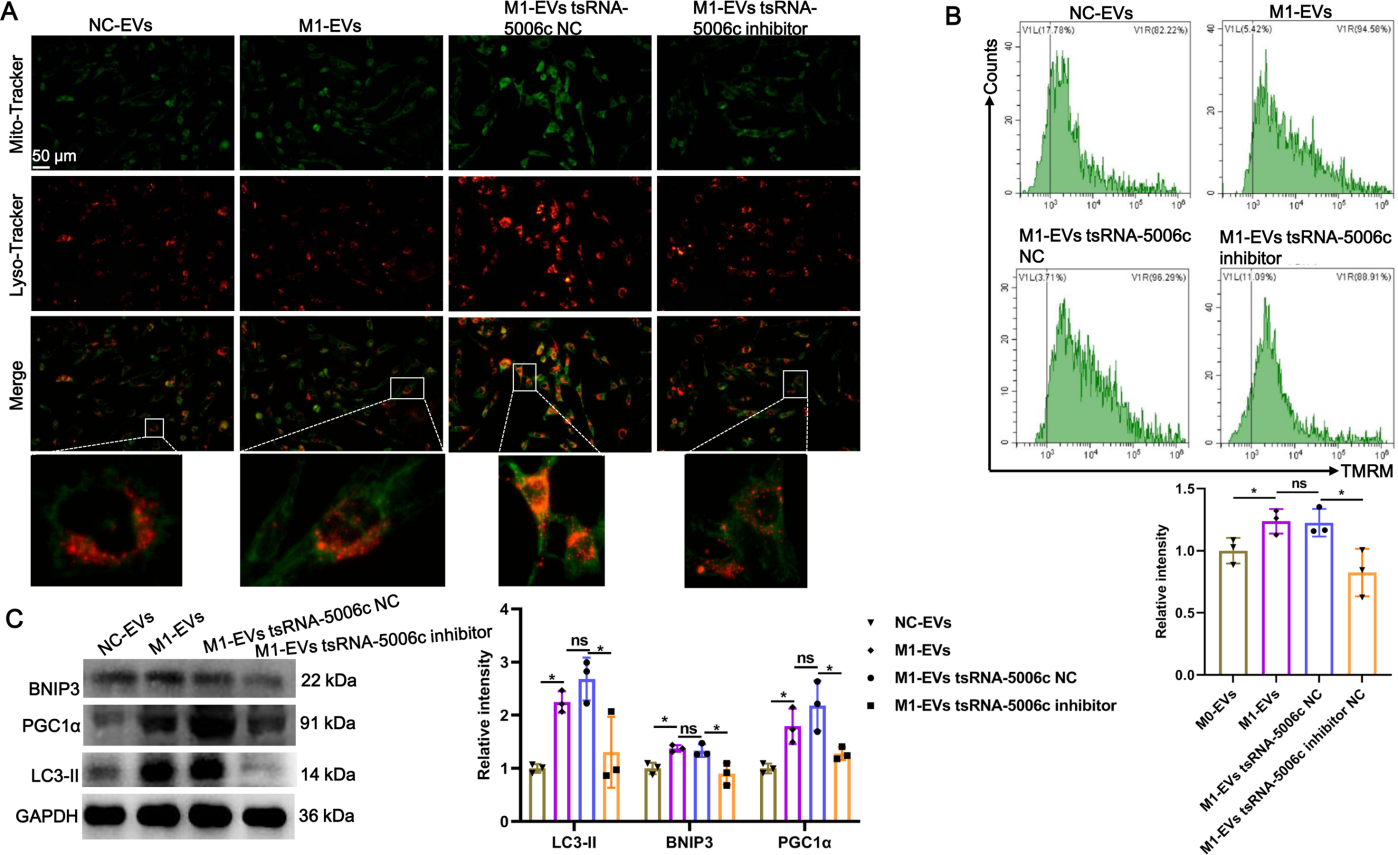
M1-EVs tsRNA-5006c enhance osteogenic differentiation of AVICs through mitophagy. (A) Co-location of Mito-Tracker Green and Lyso-Tracker Red imaging in AVICs to indicate mitophagy puncta. Scale bar: 50 µm. (B) MMP of AVICs was measured by flow cytometry using TMRM staining. (C) The expression of LC3-II, BINP3, and PGC1 α of hallmarks of mitophagy activation was detected by western blot. NC is the negative control of tsRNA-5006c inhibitor. ns *P* > 0.05, * *P* < 0.05, *** *P* < 0.001.

Recent studies have revealed that mitochondria are abundant in the heart and mitochondrial dysfunction is associated with various cardiac diseases, including cardiomyopathy (Wang et al., 2022c). Combined with the aforementioned “tsRNA-5006c—target gene—autophagy-related pathways” network results, we speculated that an enhanced osteogenic differentiation capacity by M1-EVs tsRNA-5006c may be linked to autophagy/mitophagy. To validate this conjecture, we stained lysosomes and mitochondria of AVICs after incubation M1-EVs with tsRNA-5006c-deleted (M1-EVs tsRNA-5006c inhibitor group) or not (M1-EVs tsRNA-5006c NC group) by using Lyso-Tracker (Red) and Mito-Tracker (Green), respectively. Colocation of green and red dyes allows us to readily monitor mitophagy flux in cells. As expected, mitophagosome formation of AVICs was significantly increased in the M1-EVs group compared with the NC-EVs group, whereas tsRNA-5006c interference in M1-EVs led to a significant reduce in the mitophagosome formation of AVICs (Fig. 6A). We also measured MMP of AVICs by flow cytometry using TMRM staining. As shown in Fig. 6B, M1-EVs incubation caused a significant increase of TMRM fluorescence in AVICs relative to NC-EVs, while tsRNA-5006c-deleted M1-EVs have significantly lost this ability, suggesting that M1-EVs tsRNA-5006c can enhance MMP. Besides, the expression of LC3-II, BINP3, and PGC1 α, hallmarks of mitophagy activation, were significantly elevated in the M1-EVs group compared with the NC-EVs group, but tsRNA-5006c inhibitor significantly blocked this elevation (Fig. 6C). Thus, we surmise that M1-EVs tsRNA-5006c enhance osteogenic differentiation of AVICs through mitophagy.

## Discussion

**Figure 7:**
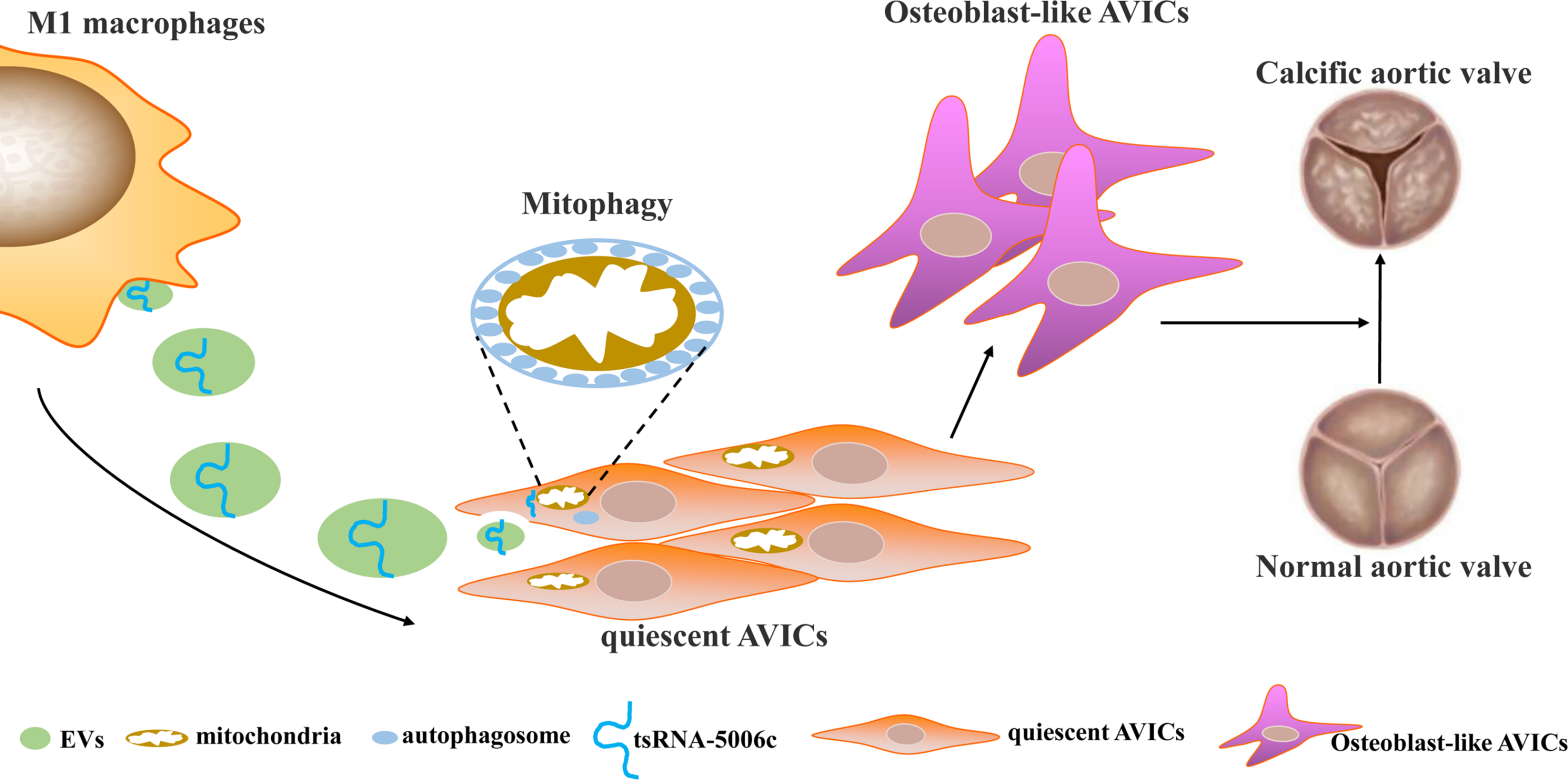
Simulation diagram. EVs is the medium of communication between M1-polarized macrophage and AVICs, and M1-EVs triggered osteogenic differentiation through delivering tsRNA-5006c to enhance mitophagy of AVICs.

Osteogenic reprogramming of AVICs has been reported to be closely related to the pathological process of CAVD (Jiao et al., 2019). Abnormal M1 polarization of macrophages partially explained osteogenic calcification of AVICs in CAVD (Lu et al., 2022). Therefore, investigating how M1-polarized macrophages trigger osteogenic differentiation of AVICs may provide a novel understanding of CAVD pathogenesis. In this study, we demonstrated that EVs are the medium of communication between M1-polarized macrophages and AVICs, and M1-EVs triggered osteogenic differentiation by delivering tsRNA-5006c to enhance the mitophagy of AVICs (Fig. 7).

In recent years, increasing studies have begun to pay attention to the function of macrophage-secreted EVs in a variety of diseases. For example, exosomal miR-212-5p secreted by M1 macrophage impaired beta cell insulin secretion by inhibiting SIRT2/Akt/GSK-3 β/β-catenin pathway in mice (Qian et al., 2021). Magnesium ion-mediated macrophage-derived exosomes could be facilitating the osteogenic differentiation of bone marrow stromal stem cells (BMSCs) by containing miR-381 (Zhu et al., 2022). Exosomal miR-222 derived from M1 macrophage also could induce apoptosis of BMSCs (Qi et al., 2021). Similarly, studies showed that M1 macrophages could promote osteogenic differentiation of BMSCs through miR-21a-5p (Liu et al., 2021). These results suggest that M1-EVs, including exosomes, play a very important role in the remote function of macrophages.Of course, these results also support our conclusion that M1 macrophages act on osteogenic differentiation of AVICs by secreting EVs. Based on this principle, a novel therapeutic approach, engineered EVs, is emerging. For instance, engineered M1 macrophage-derived exosomes significantly prevented tumor growth in mice through reprogramming tumor-associated macrophages (Gunassekaran et al., 2021), as well as enhanced sonodynamic therapy by disguising biodegradable nanoplatforms in glioblastoma (Wu et al., 2022). Therefore, we hope that the M1-EVs presented here will provide meager clues for future research into engineering EVs to treat CAVD.

In this study, we first identified tsRNA-5006c as one of the key reasons for M1-EV to promote the osteogenic differentiation of AVICs. Recently, more and more EVs-encapsulated tsRNAs of different cellular origins are uncovered. For example, Zhang et al. revealed that plasma exosomal tRF-25-R9ODMJ6B26, tRF-38-QB1MK8YUBS68BFD2, and tRF-18-BS68BFD2 might be diagnostic biomarkers for osteoporosis detection (Zhang et al., 2018). Mast cell-derived exosomal tRF-Leu-AAG-001 may have a promotion function in inflammation and angiogenesis and leucorrhea exosomal tRF-Leu-AAG-001 might be diagnostic biomarkers for endometriosis (Li et al., 2022). Alveolar macrophage-derived exosomal tRF-22-8BWS7K092 could prompt acute lung injury through the ferroptosis pathway (Wang et al., 2022b). These abundant studies not only indicate that the tsRNAs have great research value but also reflect the lack of understanding of tsRNAs at this stage. As we reported tsRNA-5006c, its function and structure have never been reported, but the interference of tsRNA-5006c expression significantly reduced the osteogenic differentiation of AVIC, leading us to believe that these never-recognized tsRNAs play a hidden role in the disease. Therefore, we will continue to invest more time, money, and effort in exploring the functions and mechanisms of tsRNAs in CAVD.

Mitophagy is the specific selective form of autophagic degradation, in which mitochondria are packaged in autophagosomes and then fuse with the lysosome to form an autolysosome and ultimately degrade mitochondria (Wang et al., 2022d). Abnormal mitophagy, including mitophagy inhibition and mitophagy excess, both disrupt mitochondrial homeostasis and contribute to disease progression. Recently, the role of mitophagy in cardiovascular and cerebrovascular diseases has attracted attention. Research published in 2021 by Giampaolo et al. is the first to demonstrate that excessive mitophagy/autophagy favors aorta osteogenic differentiation, and cardiomyocyte death, and exacerbates disease progression in calcific aortic valve stenosis (Morciano et al., 2021). Mitophagy/autophagy drives osteogenic differentiation also has been proved in the human dental pulp cells (Liu et al., 2022b), heterogeneous osteogenic cells (Fei et al., 2021), mesenchymal stem cells (Gong et al., 2021), and human gingival mesenchymal stem cells (Vidoni et al., 2019), These results are similar to those in our study, we revealed that M1-EVs treatment led to a mitophagy excess accelerating osteogenic differentiation of AVICs. Regrettably, the function of M1-EVs tsRNA-5006c on mitophagy was not verified in animals, and the relevant studies are now too few to support our conclusion. Perhaps, studies on the regulation of mitophagy by noncoding RNAs can partially support our results such as miR-155 (Tsujimoto et al., 2020) and lncRNA LOC105378097 (Liu et al., 2022a). For instance, promoting AMPK-mediated mitophagy mediated by miRNA-134-5p knockdown could rescue dendritic deficits in a mouse model of depression (Wang et al., 2022a). The circ608/miR-222/PINK1 axis mediated mitophagy regulate liver fibrosis in nonalcoholic steatohepatitis-related mice (Xu et al., 2022). In sum, the function of tsRNA-5006c on mitophagy deserve to be studied in the future.

## Conclusions

We demonstrated that EVs derived from M1-polarized macrophages could promote osteogenic differentiation of AVICs. Small RNA sequencing revealed the tsRNAs expression profile of M1-EVs and identified 45 DEtsRNAs, among which tsRNA-5006c was up-regulated in M1-EVs compared to the NC-EVs. Furthermore, we found that M1-EVs function on osteogenic differentiation of AVICs through mitophagy by delivering tsRNA-5006c. Our findings may shed novel light on therapeutic strategies for CAVD.

##  Supplemental Information

10.7717/peerj.14307/supp-1 Supplemental Information 1 The primers used in this study Note: F means forward primers, R means reverse primers, RT means reverse transcription.

10.7717/peerj.14307/supp-2 Supplemental Information 2 Raw data

## Additional Information and Declarations

### DNA Deposition

The following information was supplied regarding the deposition of DNA sequences: The sequences are available at NCBI: PRJNA868525.

### Data Availability

The following information was supplied regarding data availability: The raw measurements are available in the Supplemental Files.
