# Supplementary material for: M1 macrophage-derived extracellular vesicle containing tsRNA-5006c promotes osteogenic differentiation of aortic valve interstitial cells through regulating mitophagy
Source: PeerJ. 2022 Dec 2;10:e14307. doi: 10.7717/peerj.14307 (PMC9744173; doi:10.7717/peerj.14307)
Supplement: Supplemental Information 1 — Note: F means forward primers, R means reverse primers, RT means reverse transcription. [file peerj-10-14307-s001.docx]

| Gene | | Primers (5’ - 3’) | |
| --- | --- | --- | --- |
| GAPDH-F | | CAAAATGGTGAAGGTCGGTGT | |
| GAPDH-R | | GAGGTCAATGAAGGGGTCGTT | |
| iNOS-F | | GGAGTGACGGCAAACATGACT | |
| iNOS-R | | TCGATGCACAACTGGGTGAAC | |
| IL-1β-F | | TGTGTCTTTCCCGTGGACCTT | |
| IL-1β-R | | TGTTCATCTCGGAGCCTGTAGTG | |
| TNF-α-F | | GCCCAGACCCTCACACTCAG | |
| TNF-α-R | | ACTTGGTGGTTTGCTACGACG | |
| RUNX2-F | | GAATGCTTCATTCGCCTCACA |  |
| RUNX2-R | | GTGACCTGCGGAGATTAACCA |  |
| SPP1-F | | CTCCATTGACTCGAACGACTC |  |
| SPP1-R | | CAGGTCTGCGAAACTTCTTAGAT |  |
| BMP2-F | | ACGAGGTCCTGAGCGAGTTC |  |
| BMP2-R | | GACCTGAGTGCCTGCGATA |  |
| U6-F | | CGATACAGAGAAGATTAGCATGGC |  |
| U6-R | | AACGCTTCACGAATTTGCGT |  |
| tsRNA-3034a-RT | | GTCGTATCCAGTGCGTGTCGTGGAGTCGGCAATTGCACTGGATACGACTGGCGAC |  |
| tsRNA-3010b-RT | | GTCGTATCCAGTGCGTGTCGTGGAGTCGGCAATTGCACTGGATACGACTGGTACC |  |
| tsRNA-5005b -RT | | GTCGTATCCAGTGCGTGTCGTGGAGTCGGCAATTGCACTGGATACGACTCTGATG |  |
| tsRNA-5006c-RT | | GTCGTATCCAGTGCGTGTCGTGGAGTCGGCAATTGCACTGGATACGACGTCCCAT |  |
| tsRNA-3038b-RT | | GTCGTATCCAGTGCGTGTCGTGGAGTCGGCAATTGCACTGGATACGACTGGTGGC |  |
| tsRNA-3034a-F | | GCAGGTCCCACCAGAG |  |
| tsRNA-3010b-F | | GAACCCCACTCCTGGT |  |
| tsRNA-5005b -F | | GGATAGCTCAGTCGGTAGAG |  |
| tsRNA-5006c-F | | GCTCAGTCGGTAGAGCA |  |
| tsRNA-3038b-F | | TCGATCCCCGTACGG |  |
| tsRNA-5006c inhibitor | | GTCCCATCGTCTACCGACTGAGCTAGCCGGGC |  |
| tsRNA-5006c inhibitor NC | | UUCUCCGAACGUGUCACGUTT  ACGUGACACGUUCGGAGAATT |  |
