# Supplementary figures and images for: M1 macrophage-derived extracellular vesicle containing tsRNA-5006c promotes osteogenic differentiation of aortic valve interstitial cells through regulating mitophagy
Source: PeerJ. 2022 Dec 2;10:e14307. doi: 10.7717/peerj.14307 (PMC9744173; doi:10.7717/peerj.14307)

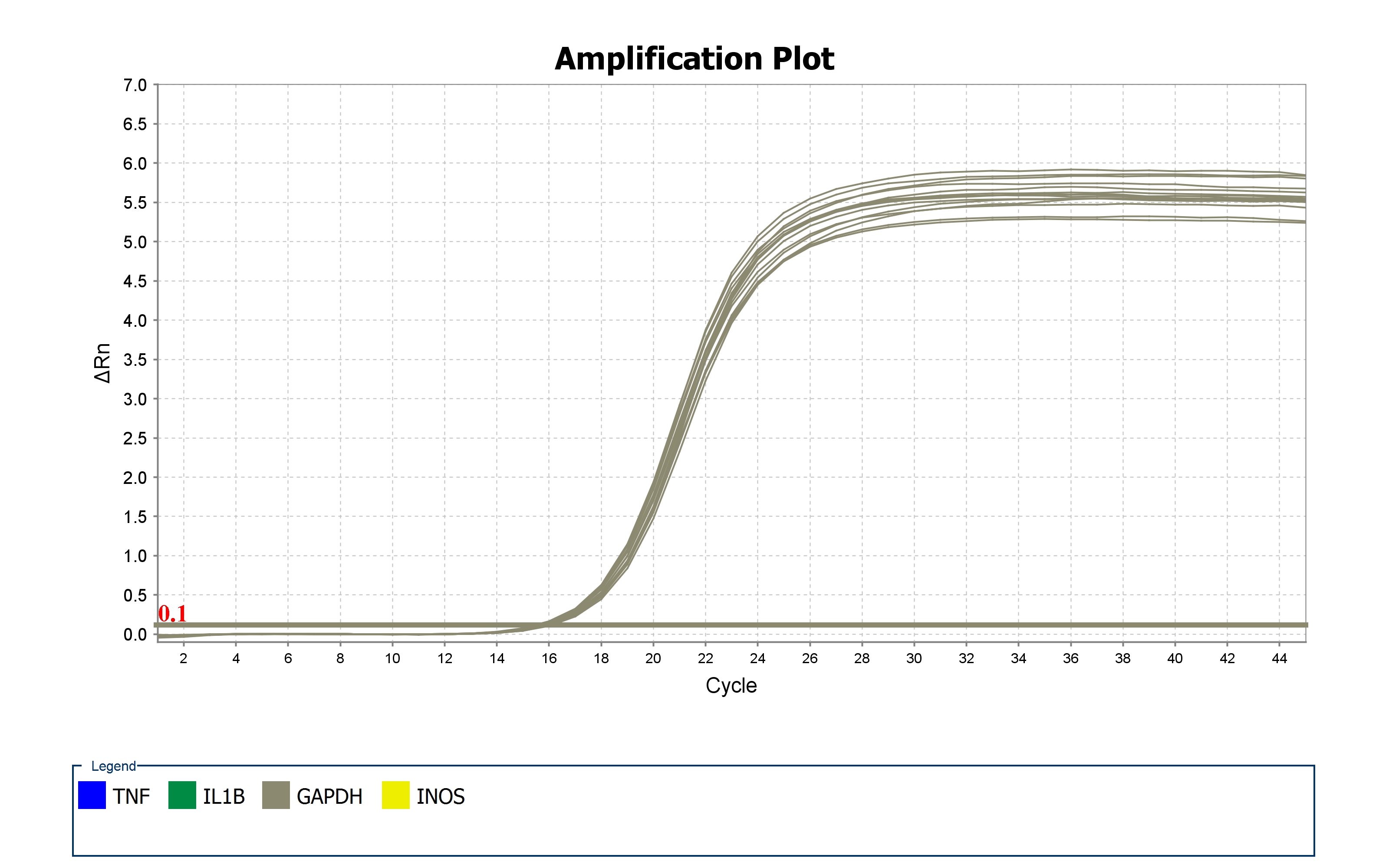

Supplement: Supplemental Information 2 [file peerj-10-14307-s002.zip › Raw data/Figure 1B RT-qPCR/Raw data/Amplification Plot GAPDH.jpg]

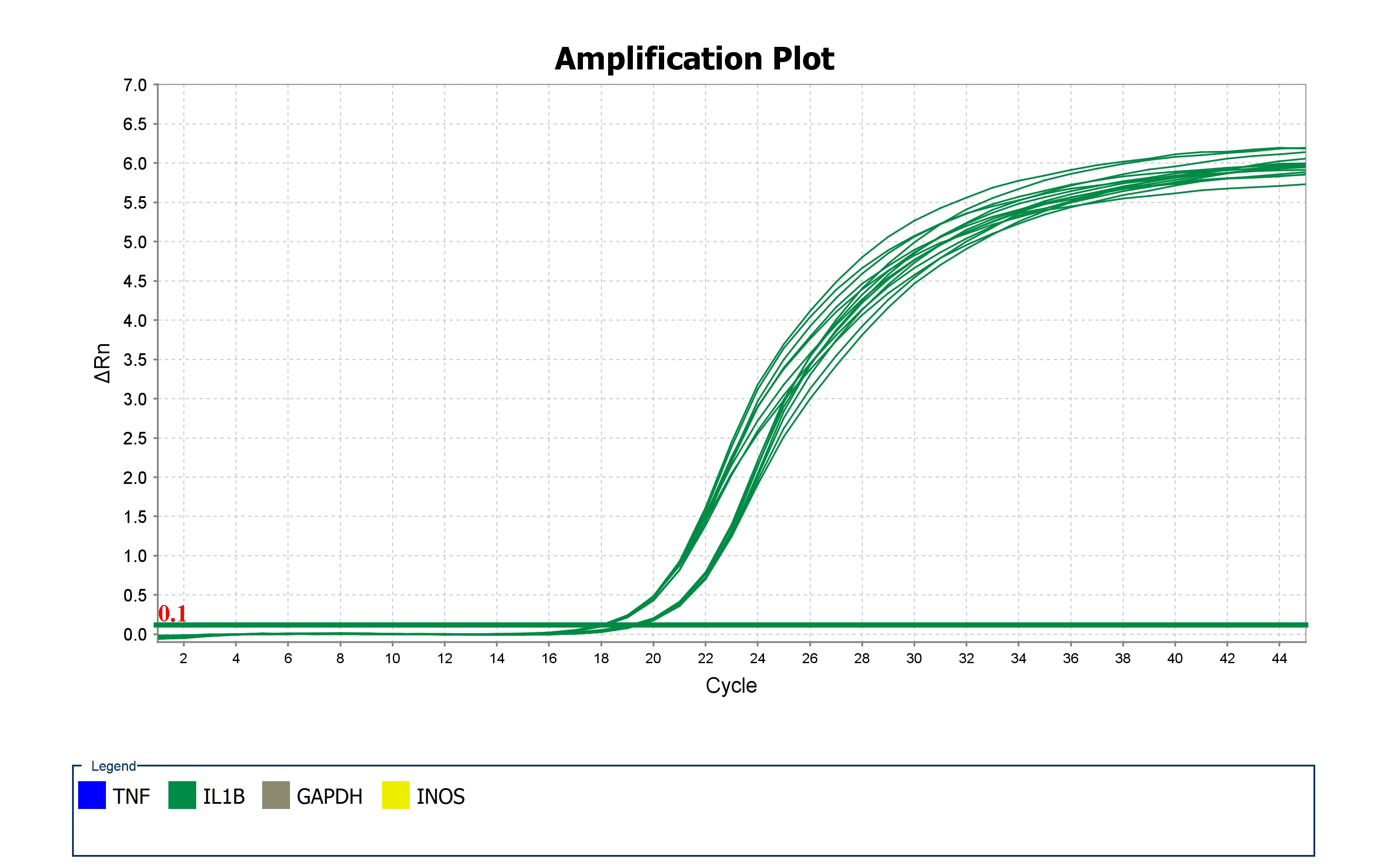

Supplement: Supplemental Information 2 [file peerj-10-14307-s002.zip › Raw data/Figure 1B RT-qPCR/Raw data/Amplification Plot IL1B.jpg]

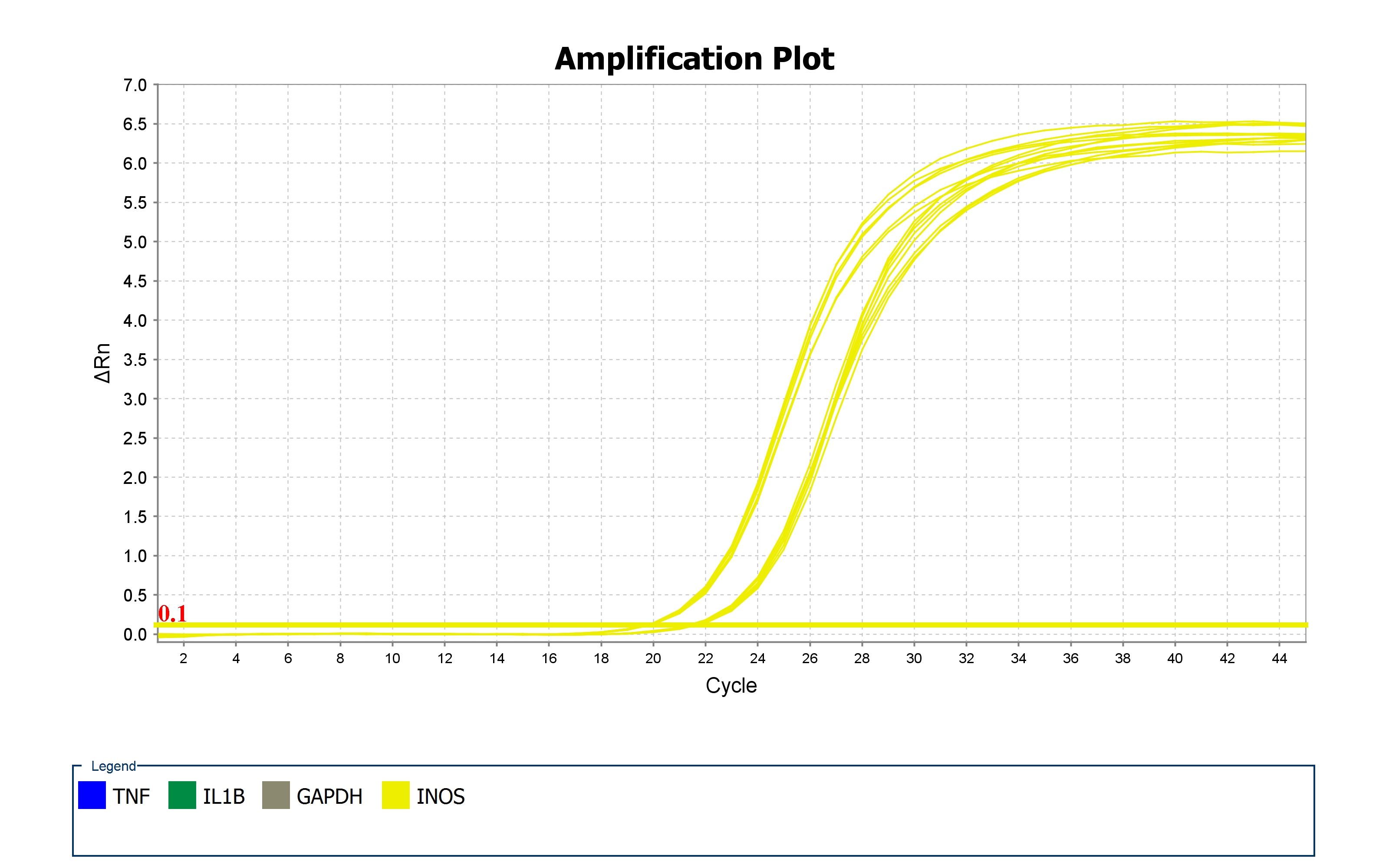

Supplement: Supplemental Information 2 [file peerj-10-14307-s002.zip › Raw data/Figure 1B RT-qPCR/Raw data/Amplification Plot INOS.jpg]

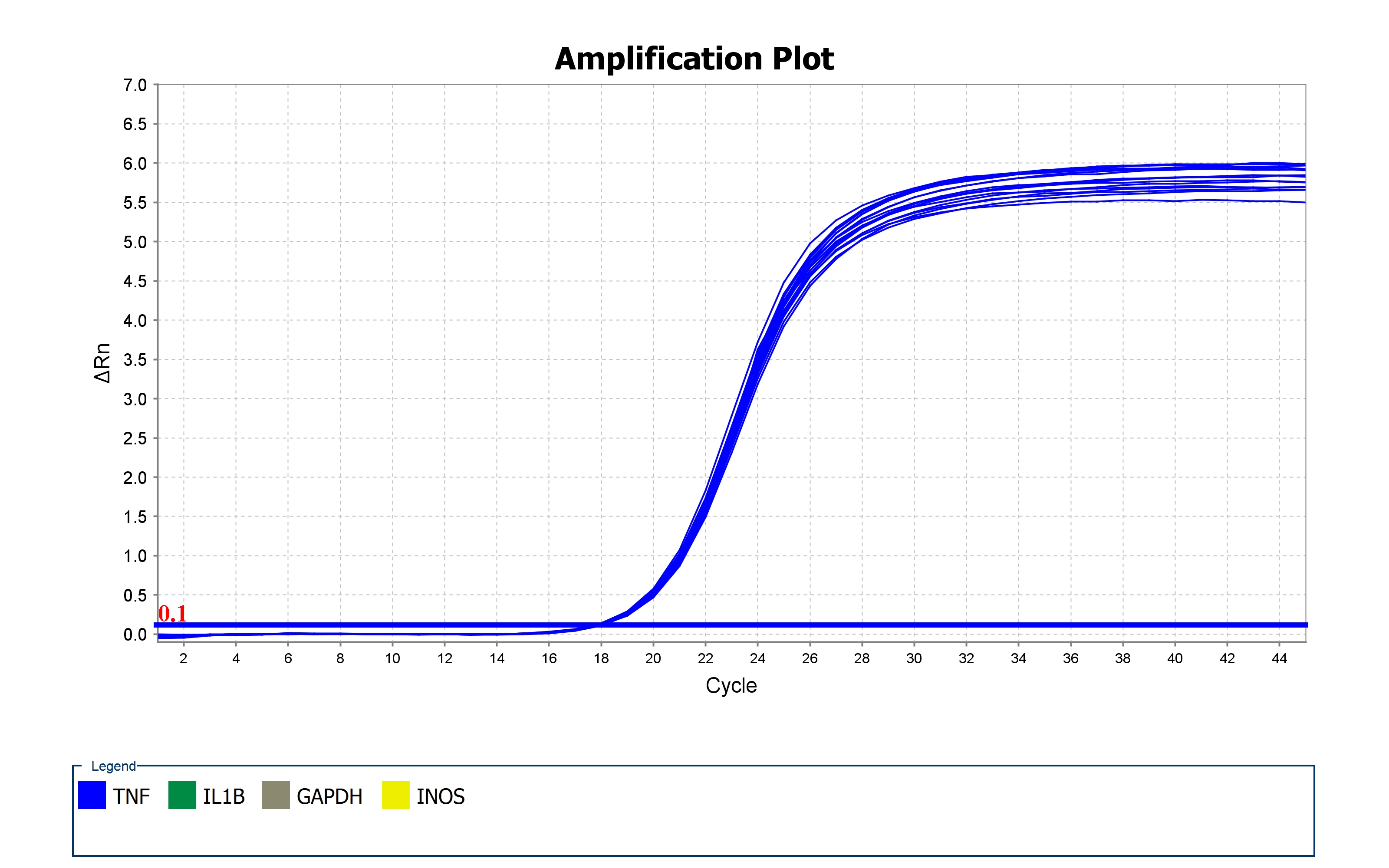

Supplement: Supplemental Information 2 [file peerj-10-14307-s002.zip › Raw data/Figure 1B RT-qPCR/Raw data/Amplification Plot TNF.jpg]

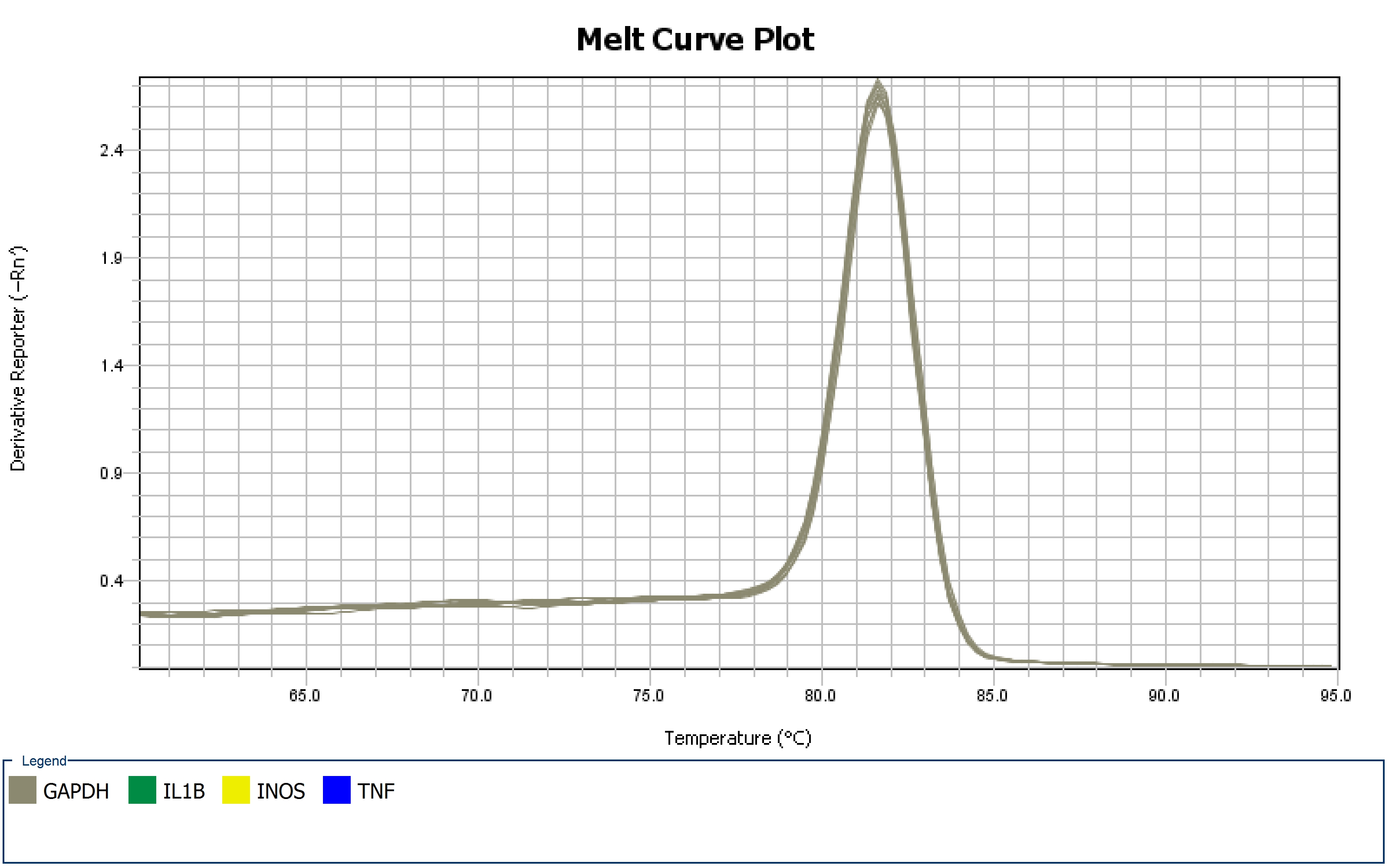

Supplement: Supplemental Information 2 [file peerj-10-14307-s002.zip › Raw data/Figure 1B RT-qPCR/Raw data/Melt Curve Plot GAPDH.jpg]

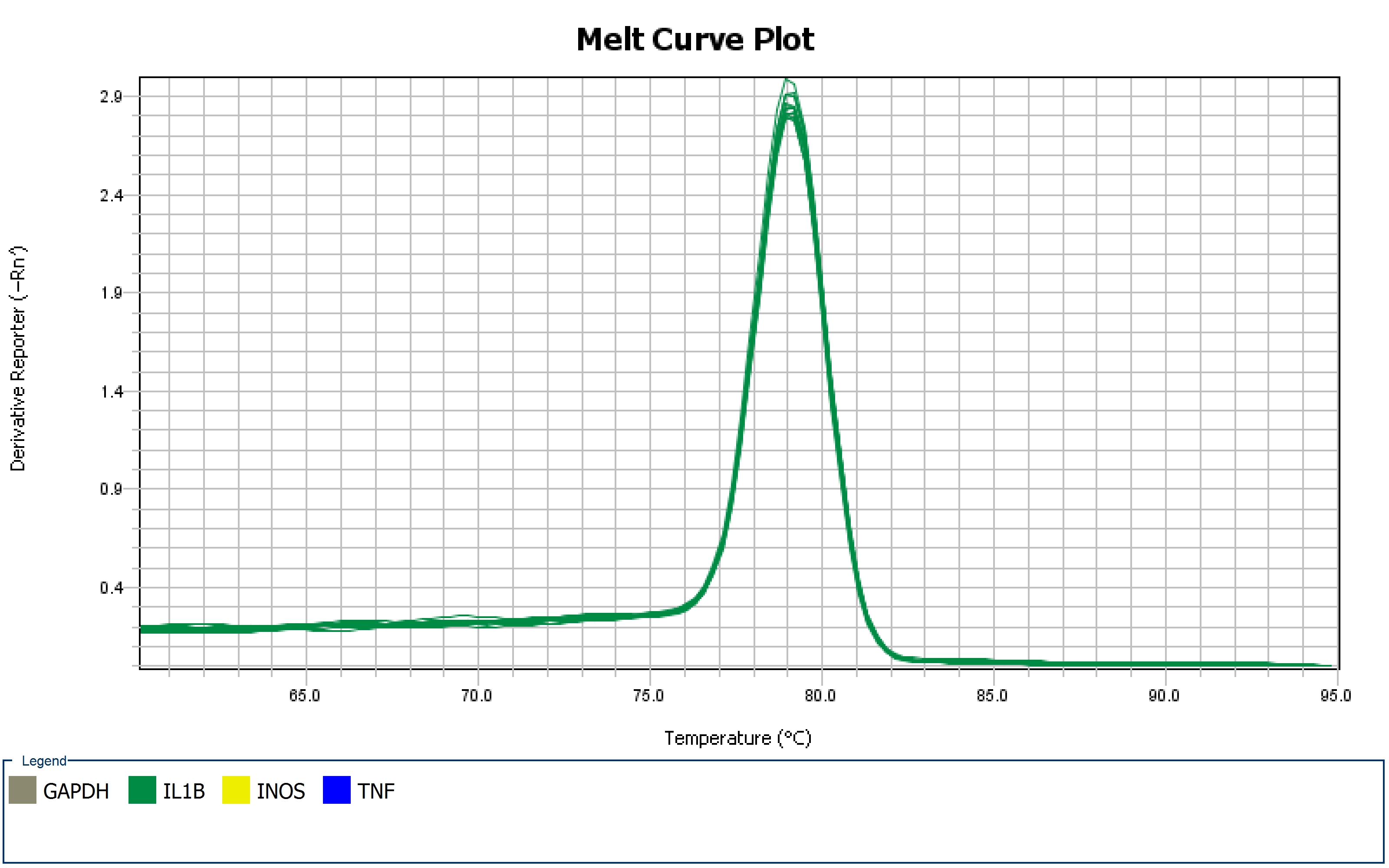

Supplement: Supplemental Information 2 [file peerj-10-14307-s002.zip › Raw data/Figure 1B RT-qPCR/Raw data/Melt Curve Plot IL1B.jpg]

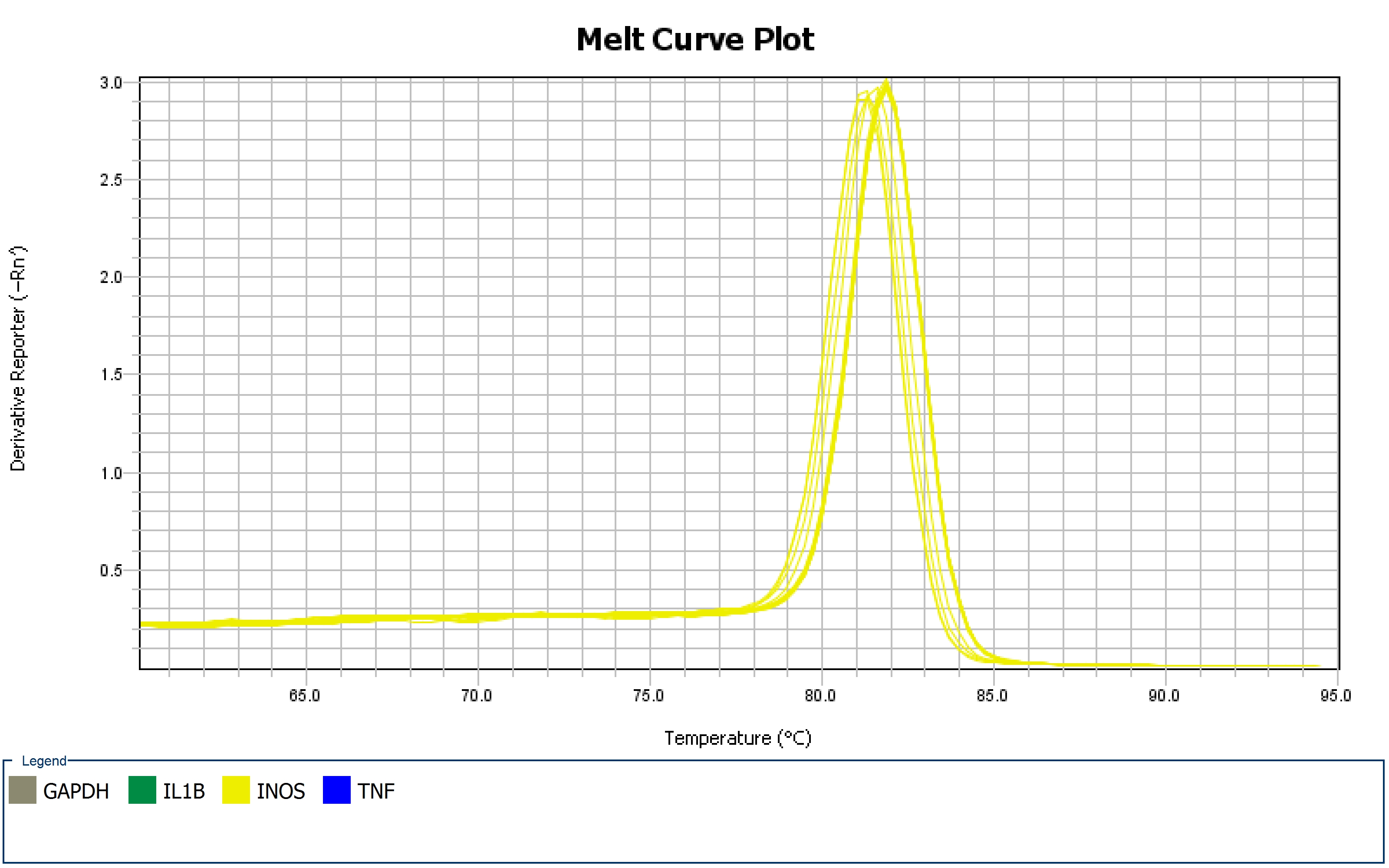

Supplement: Supplemental Information 2 [file peerj-10-14307-s002.zip › Raw data/Figure 1B RT-qPCR/Raw data/Melt Curve Plot INOS.jpg]

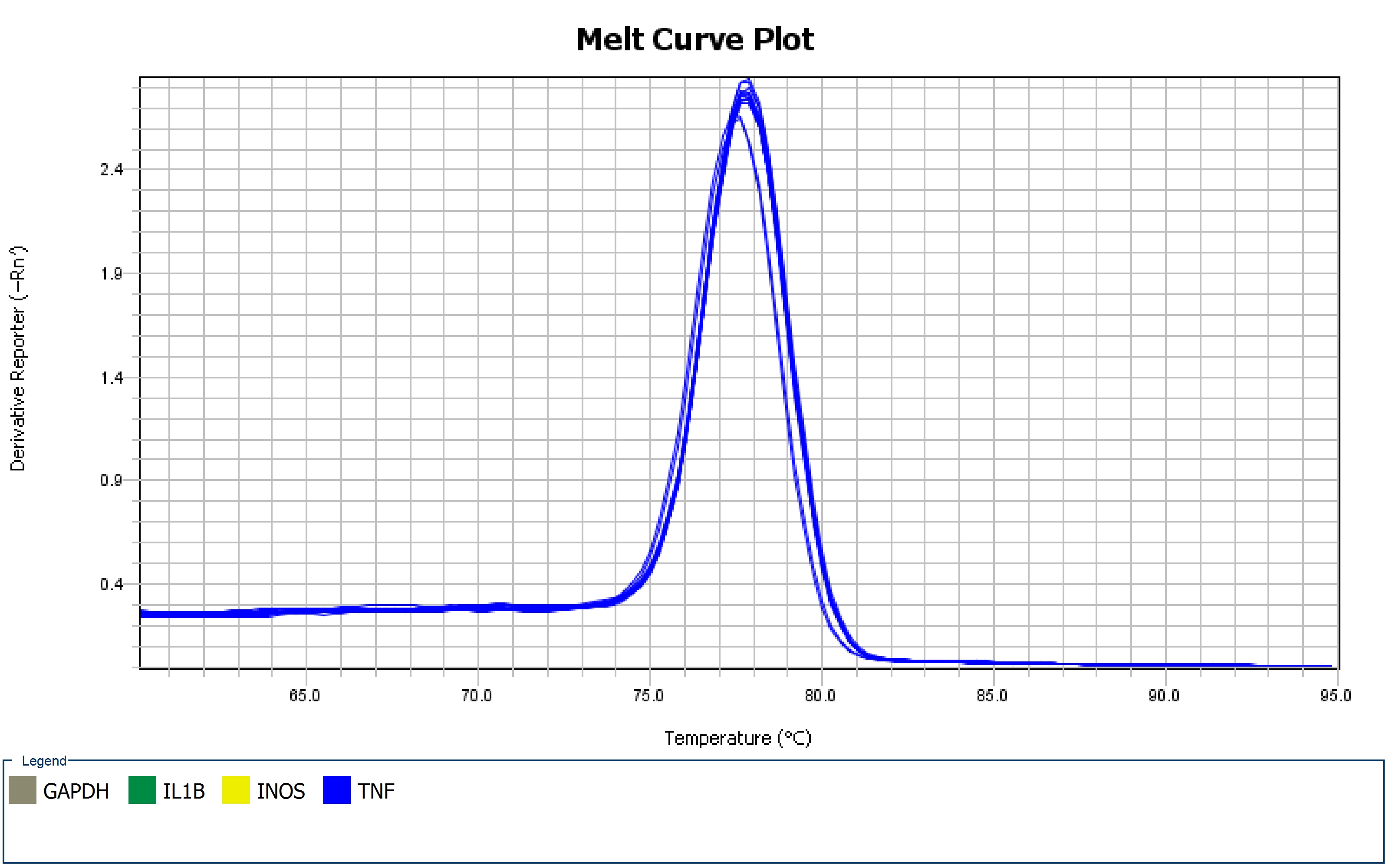

Supplement: Supplemental Information 2 [file peerj-10-14307-s002.zip › Raw data/Figure 1B RT-qPCR/Raw data/Melt Curve Plot TNF.jpg]

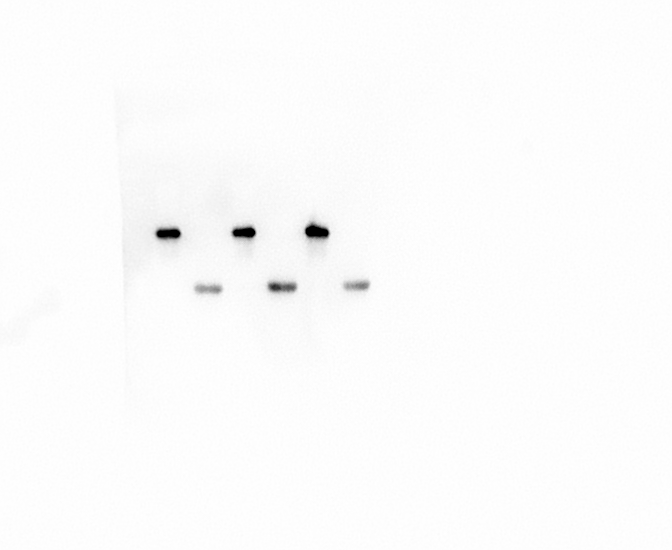

Supplement: Supplemental Information 2 [file peerj-10-14307-s002.zip › Raw data/Figure 1F WB/GAPDH+CD63.tif]

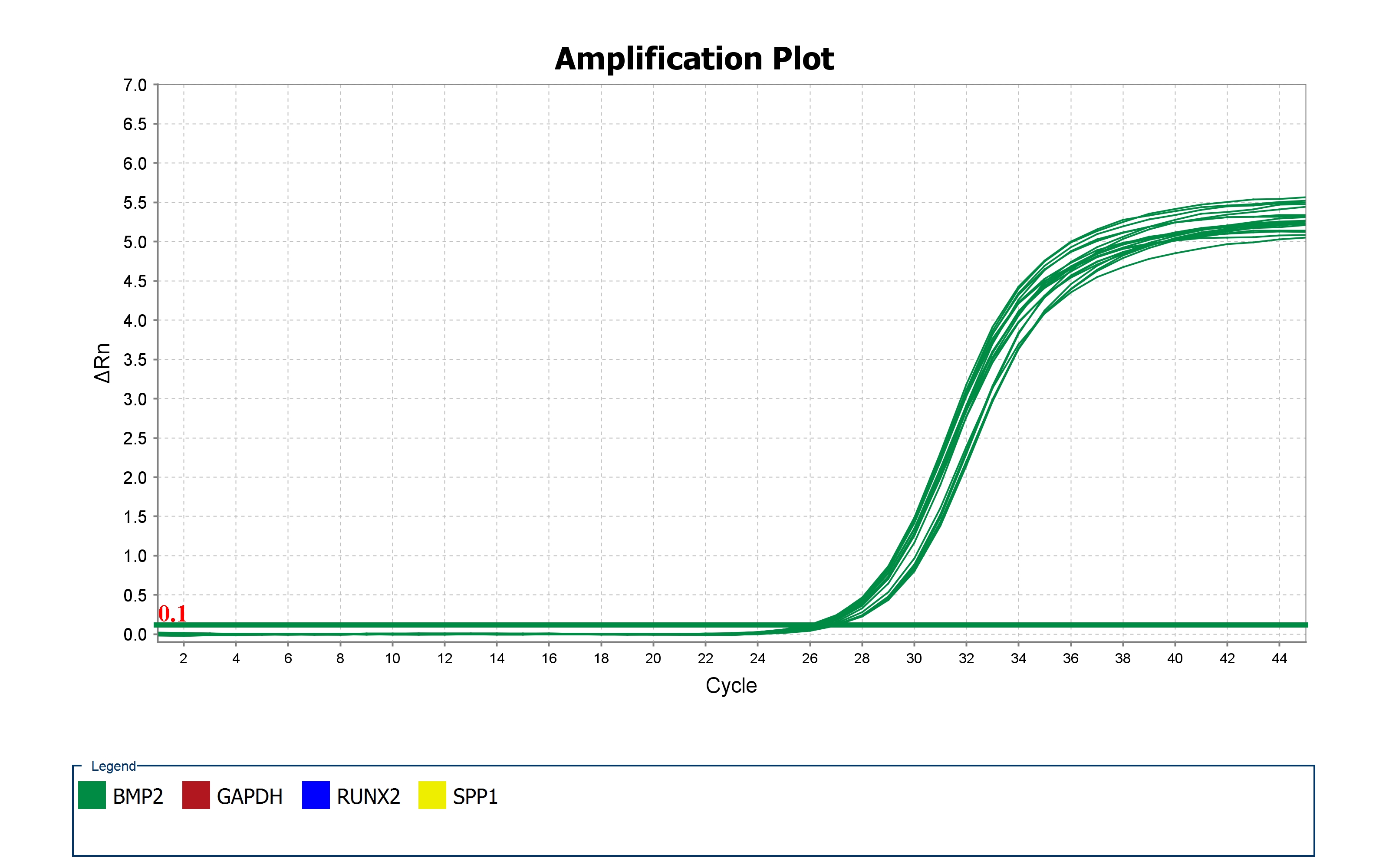

Supplement: Supplemental Information 2 [file peerj-10-14307-s002.zip › Raw data/Figure 2C RT-qPCR/Raw data/Amplification Plot BMP2.jpg]

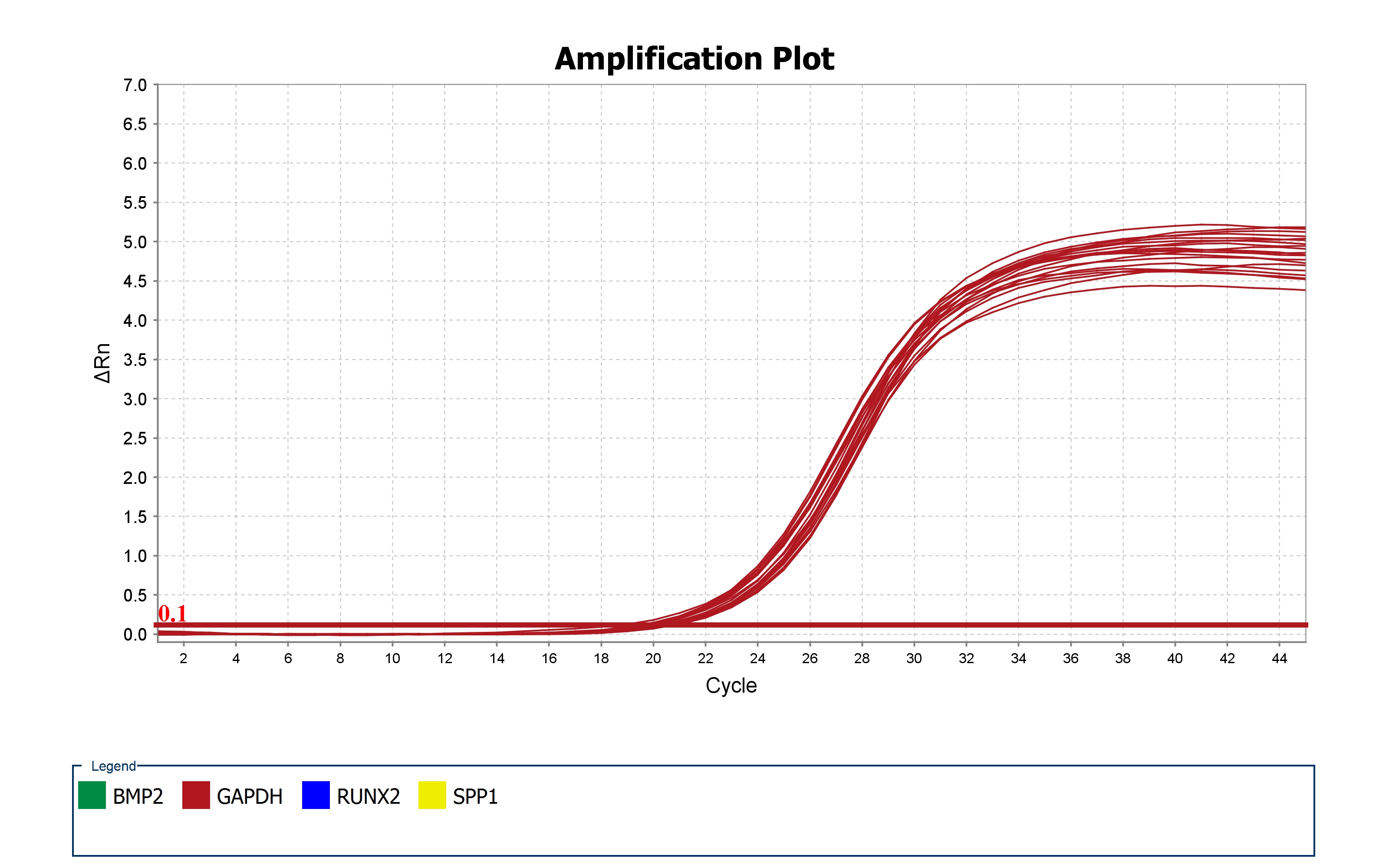

Supplement: Supplemental Information 2 [file peerj-10-14307-s002.zip › Raw data/Figure 2C RT-qPCR/Raw data/Amplification Plot GAPDH.jpg]

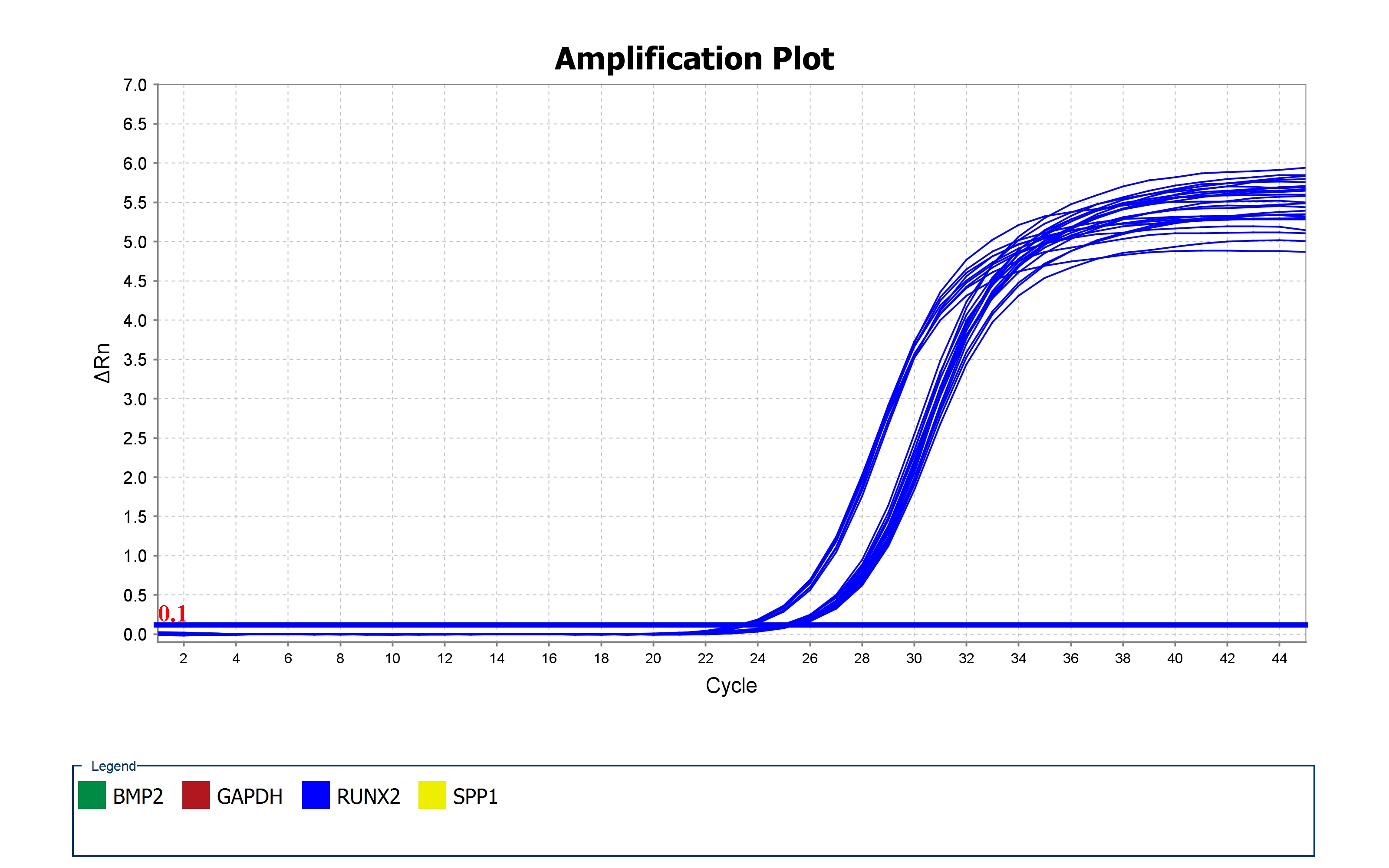

Supplement: Supplemental Information 2 [file peerj-10-14307-s002.zip › Raw data/Figure 2C RT-qPCR/Raw data/Amplification Plot RUNX2.jpg]

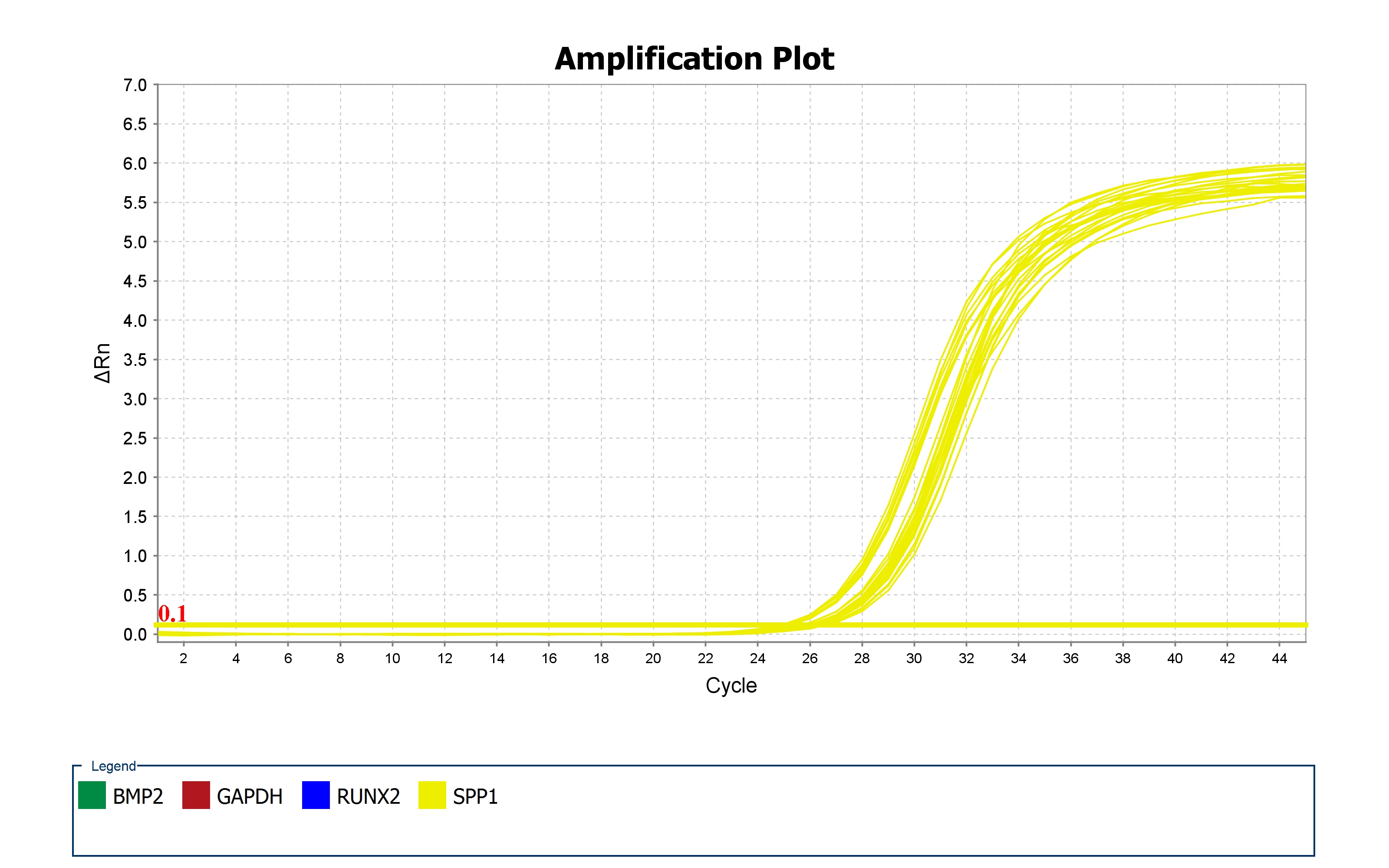

Supplement: Supplemental Information 2 [file peerj-10-14307-s002.zip › Raw data/Figure 2C RT-qPCR/Raw data/Amplification Plot SPP1.jpg]

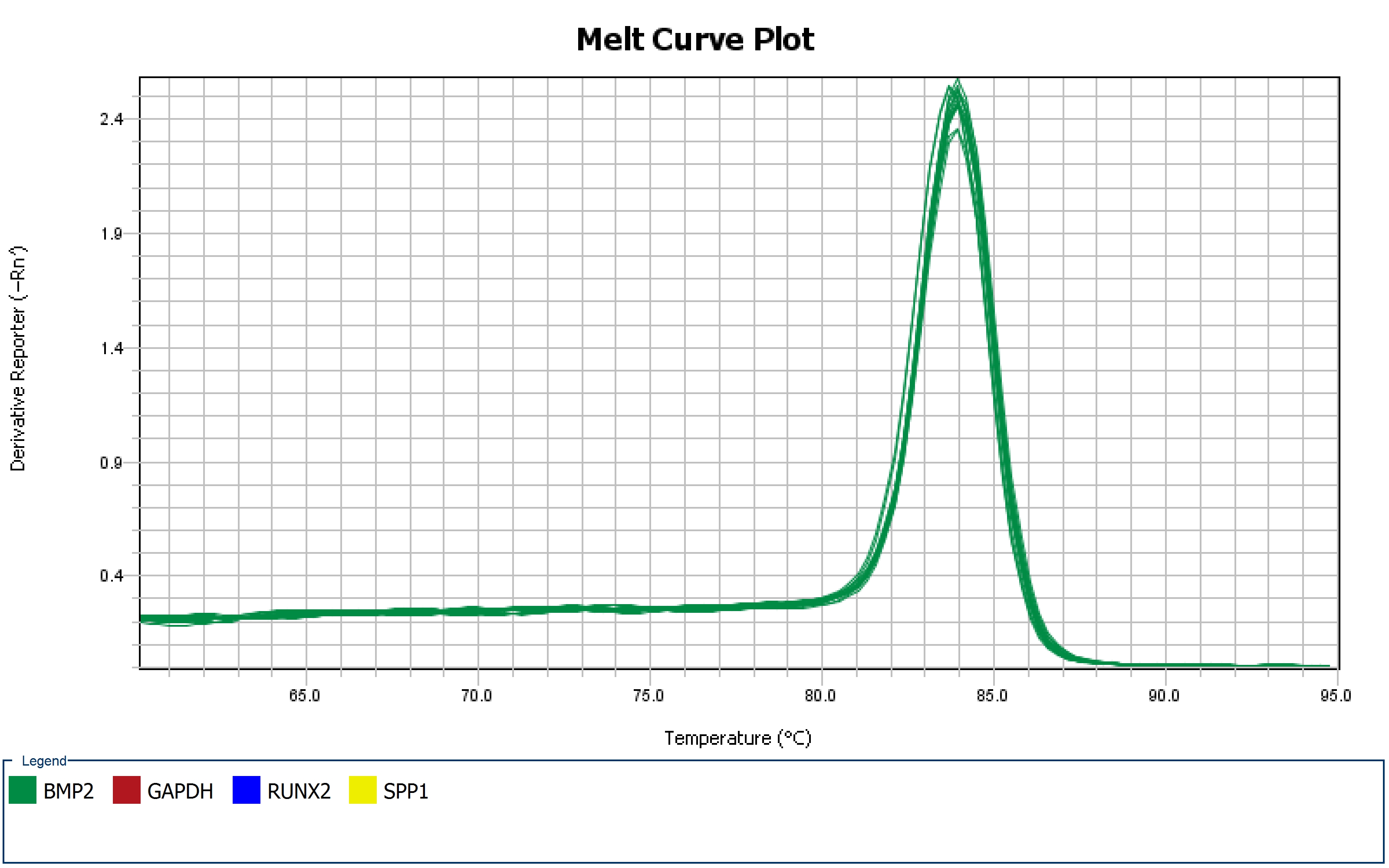

Supplement: Supplemental Information 2 [file peerj-10-14307-s002.zip › Raw data/Figure 2C RT-qPCR/Raw data/Melt Curve Plot BMP2.jpg]

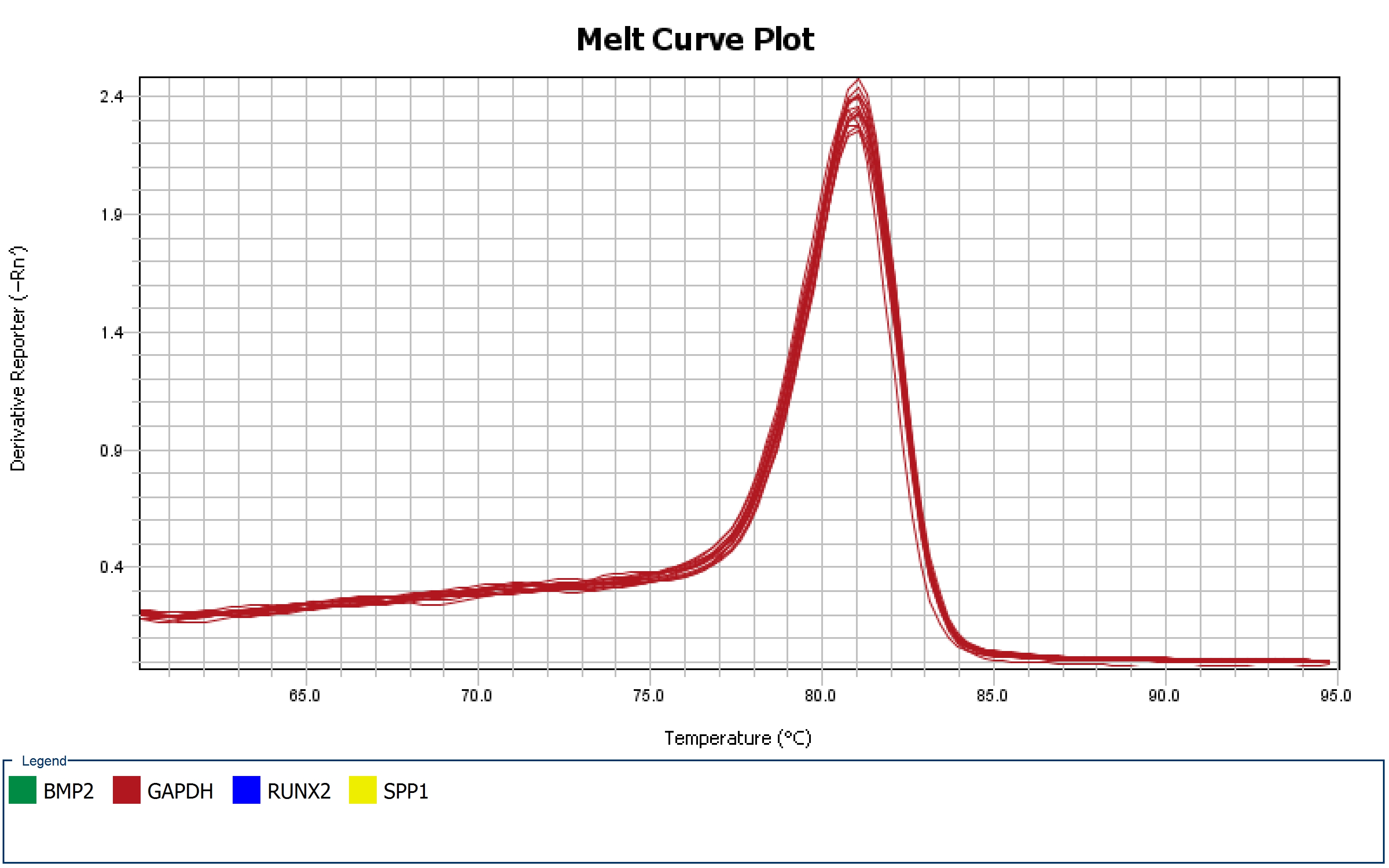

Supplement: Supplemental Information 2 [file peerj-10-14307-s002.zip › Raw data/Figure 2C RT-qPCR/Raw data/Melt Curve Plot GAPDH.jpg]

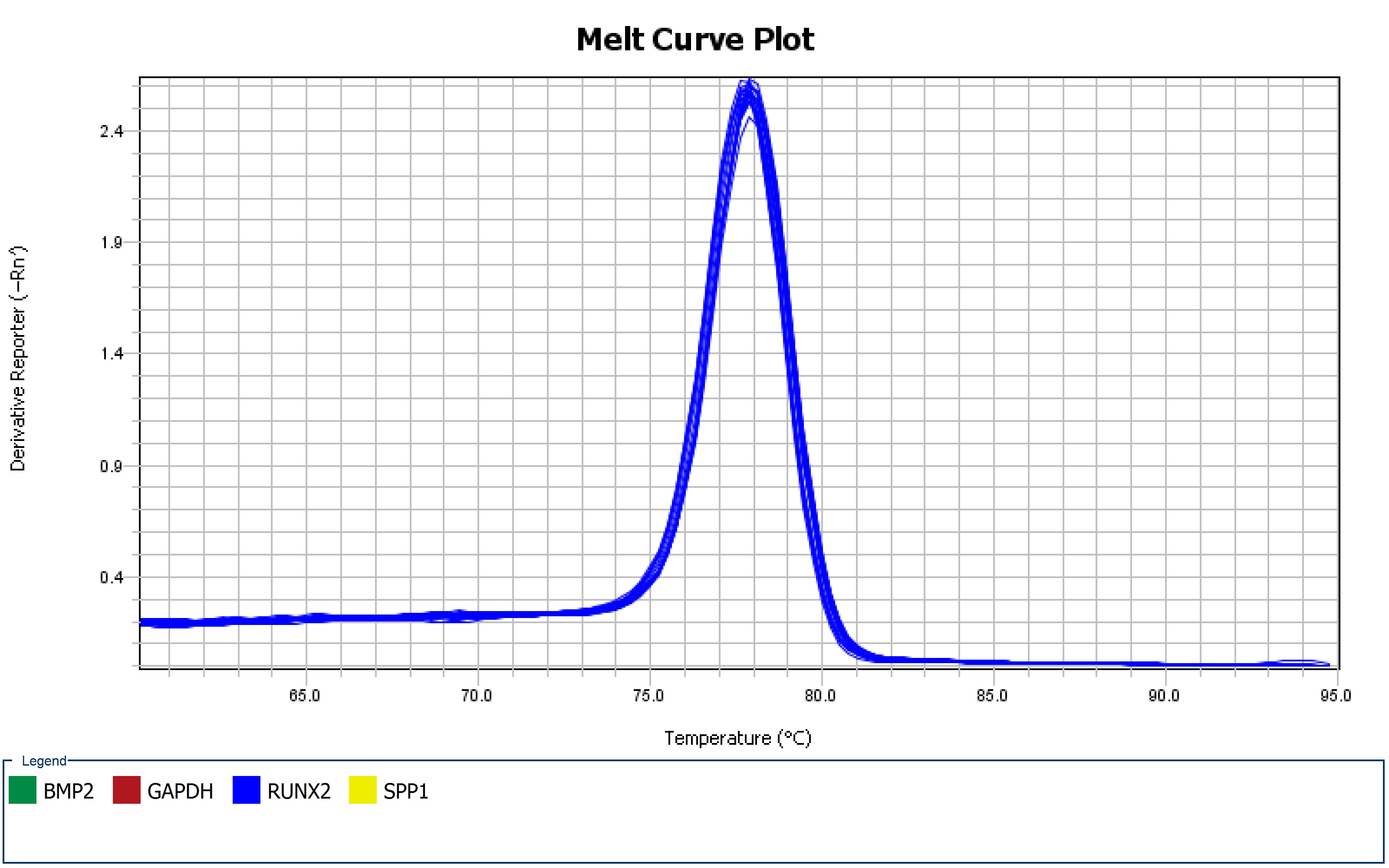

Supplement: Supplemental Information 2 [file peerj-10-14307-s002.zip › Raw data/Figure 2C RT-qPCR/Raw data/Melt Curve Plot RUNX2.jpg]

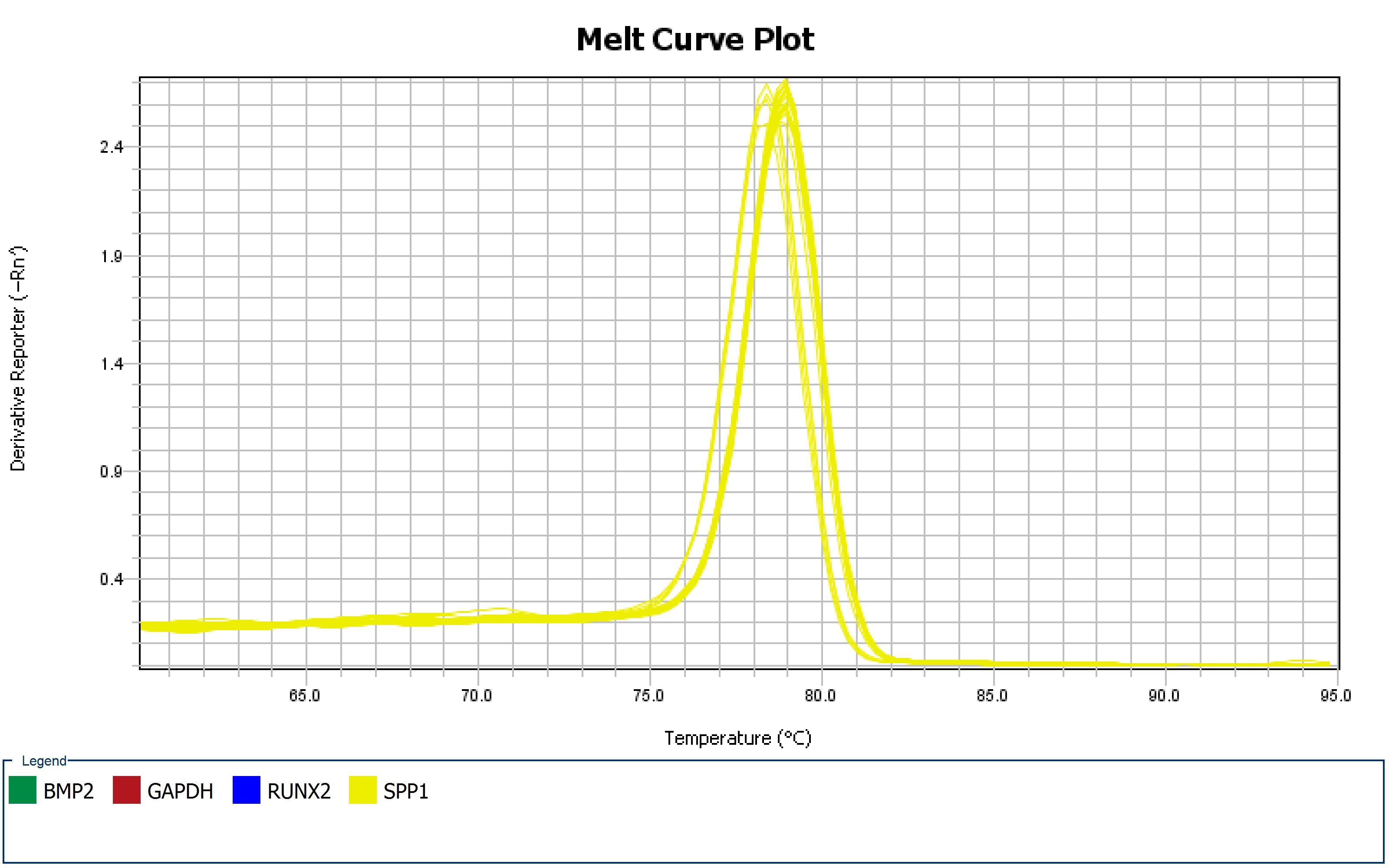

Supplement: Supplemental Information 2 [file peerj-10-14307-s002.zip › Raw data/Figure 2C RT-qPCR/Raw data/Melt Curve Plot SPP1.jpg]

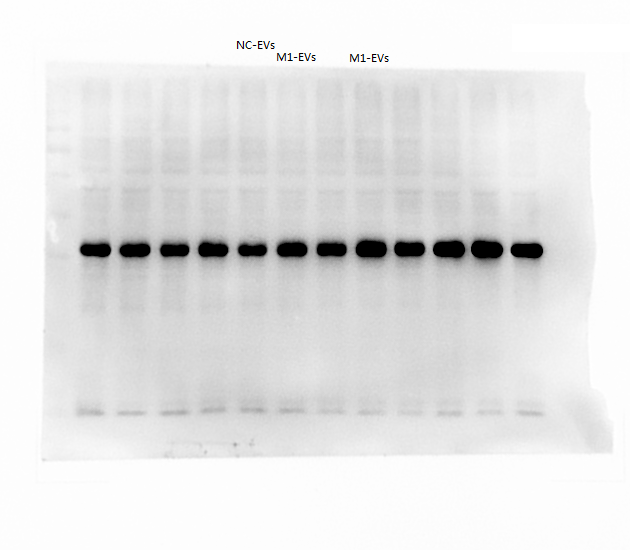

Supplement: Supplemental Information 2 [file peerj-10-14307-s002.zip › Raw data/Figure 2D WB/GAPDH.tif]

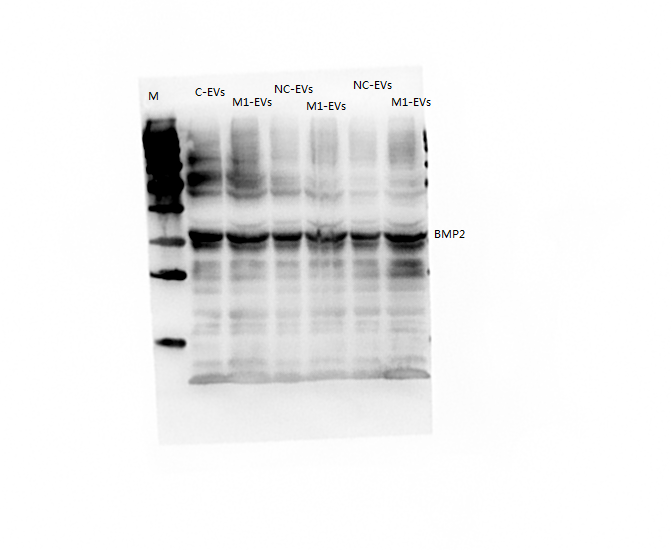

Supplement: Supplemental Information 2 [file peerj-10-14307-s002.zip › Raw data/Figure 2D WB/bmp2.tif]

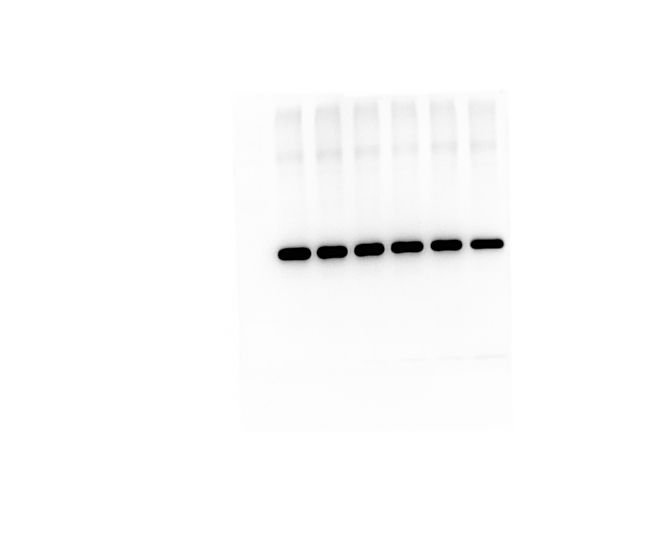

Supplement: Supplemental Information 2 [file peerj-10-14307-s002.zip › Raw data/Figure 2E WB/GAPDH.tif]

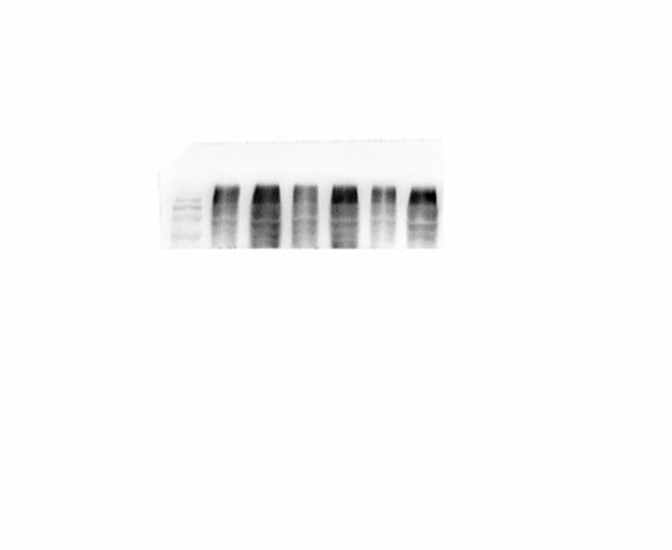

Supplement: Supplemental Information 2 [file peerj-10-14307-s002.zip › Raw data/Figure 2E WB/collagen I.tif]

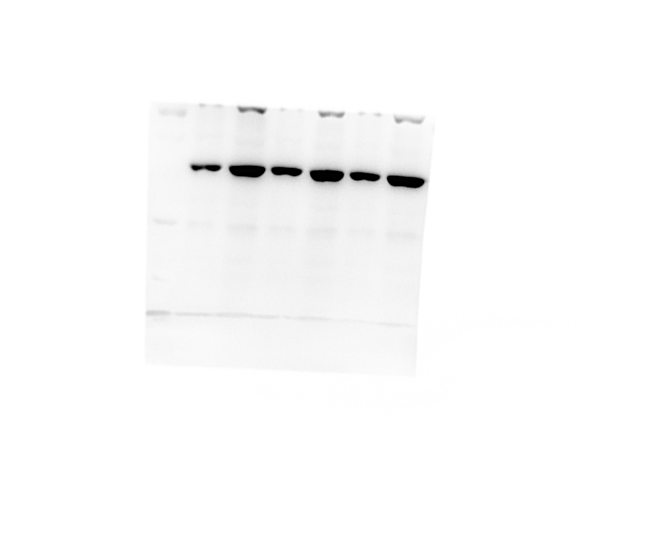

Supplement: Supplemental Information 2 [file peerj-10-14307-s002.zip › Raw data/Figure 2E WB/a┴-SMA.tif]

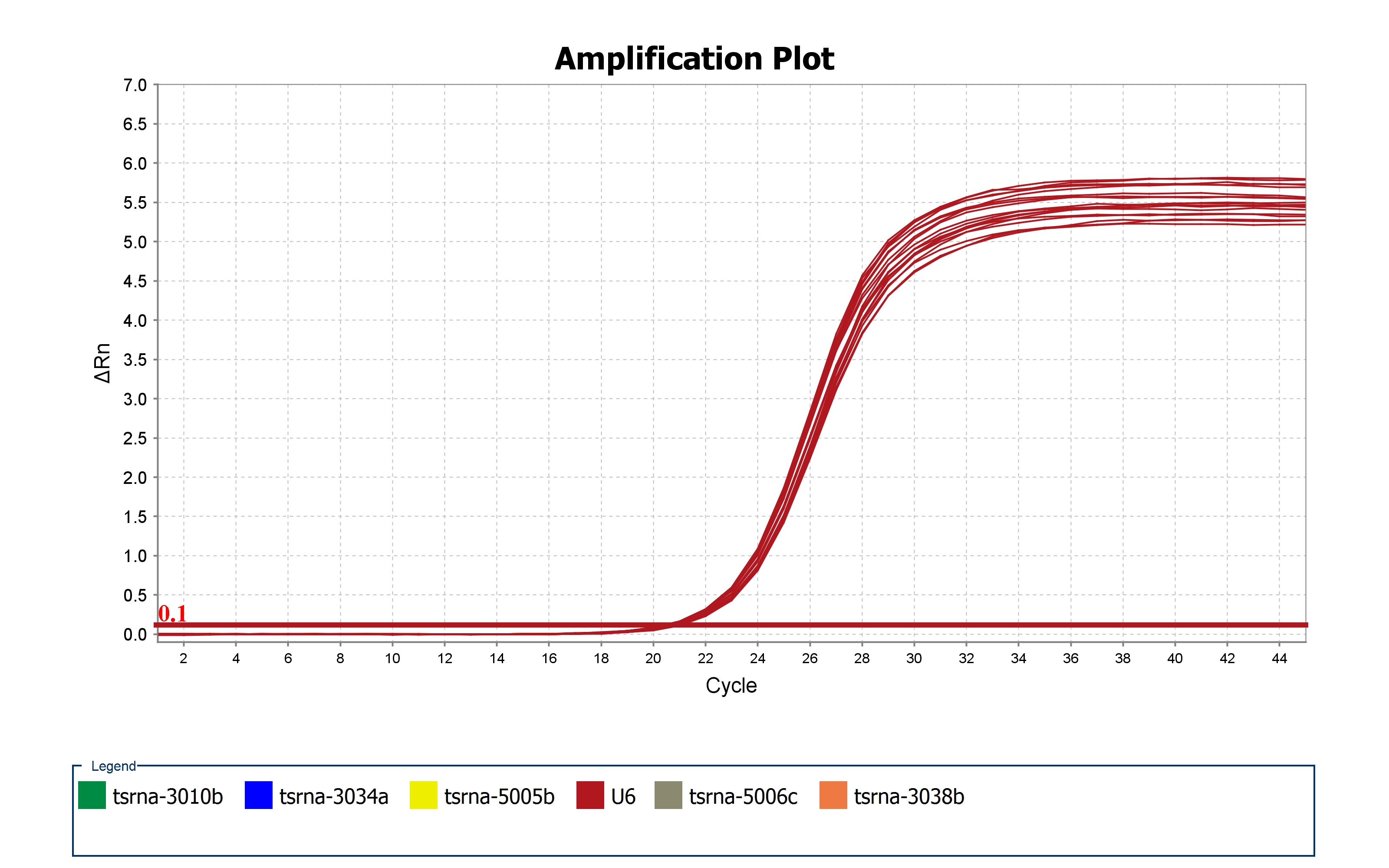

Supplement: Supplemental Information 2 [file peerj-10-14307-s002.zip › Raw data/Figure 4A RT-qPCR/Raw data/Amplification Plot U6.jpg]

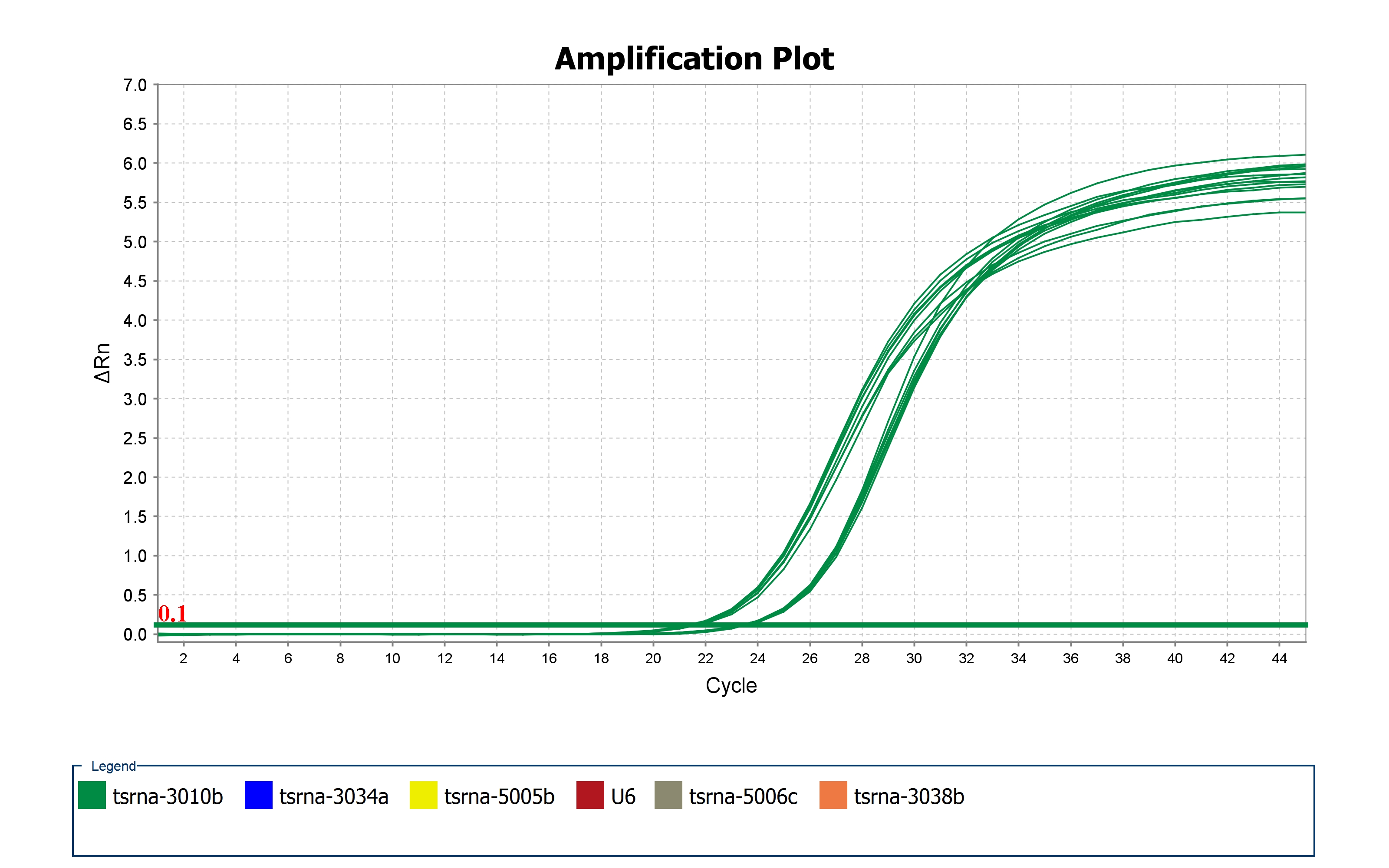

Supplement: Supplemental Information 2 [file peerj-10-14307-s002.zip › Raw data/Figure 4A RT-qPCR/Raw data/Amplification Plot tsrna-3010b.jpg]

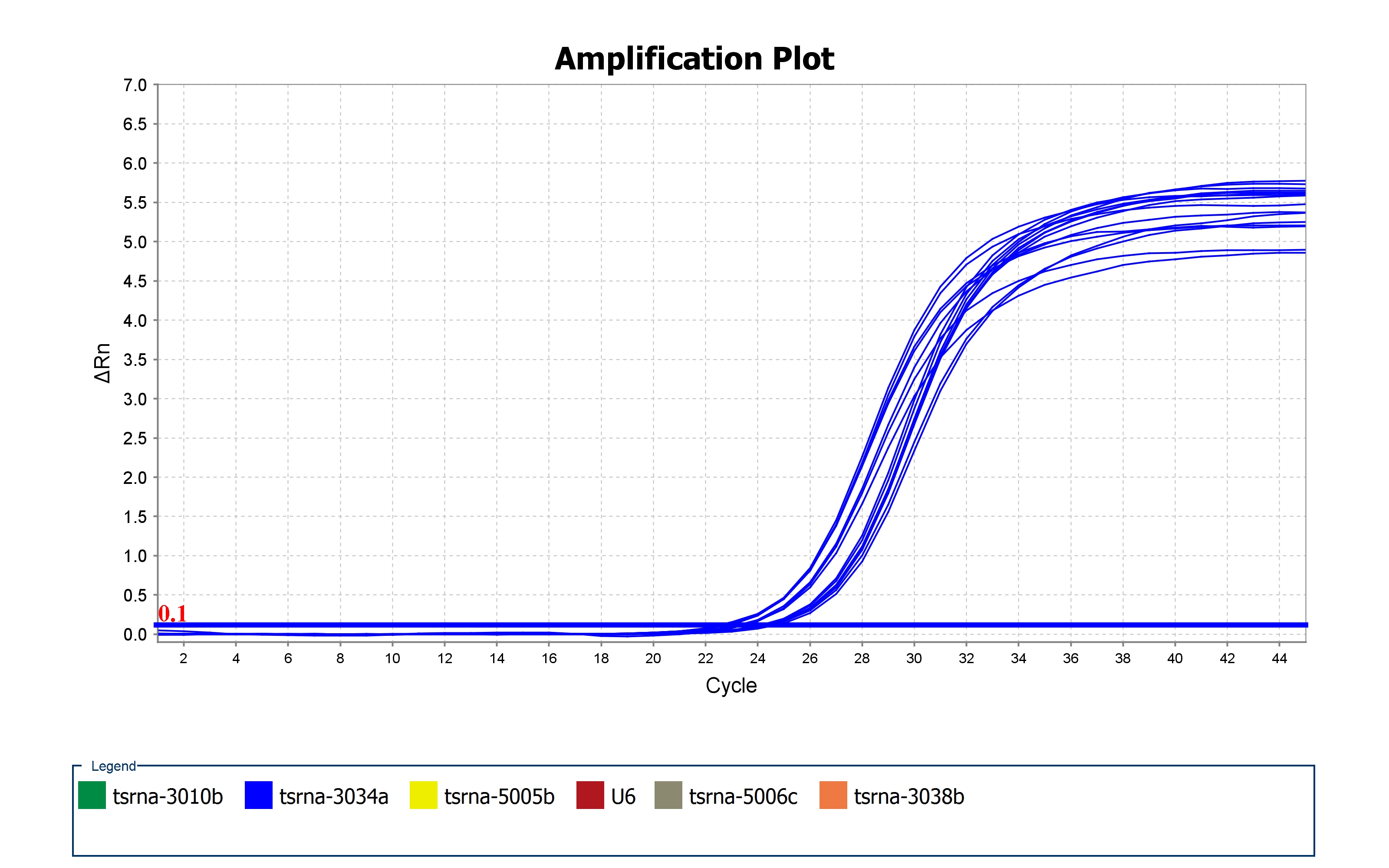

Supplement: Supplemental Information 2 [file peerj-10-14307-s002.zip › Raw data/Figure 4A RT-qPCR/Raw data/Amplification Plot tsrna-3034a.jpg]

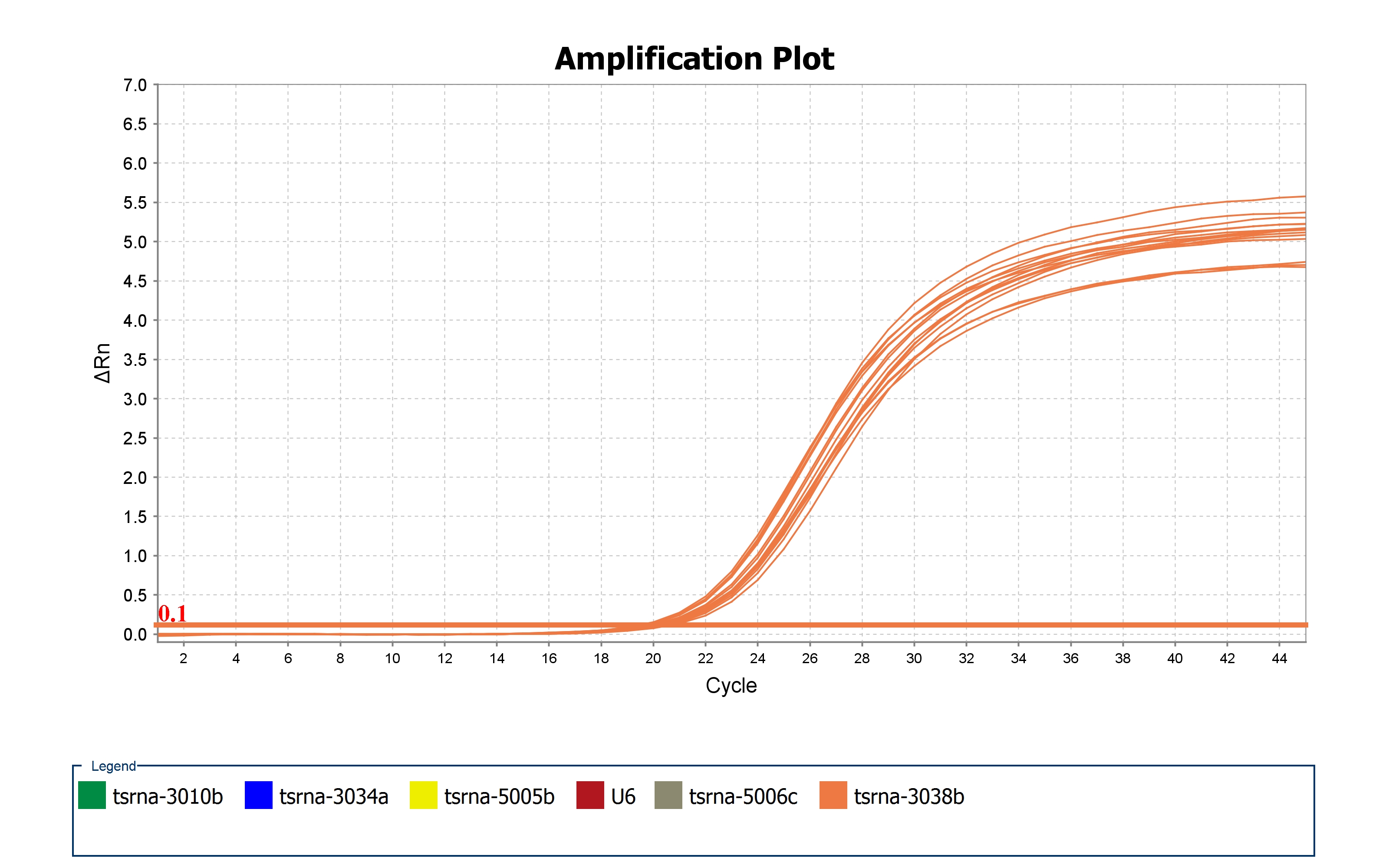

Supplement: Supplemental Information 2 [file peerj-10-14307-s002.zip › Raw data/Figure 4A RT-qPCR/Raw data/Amplification Plot tsrna-3038b.jpg]

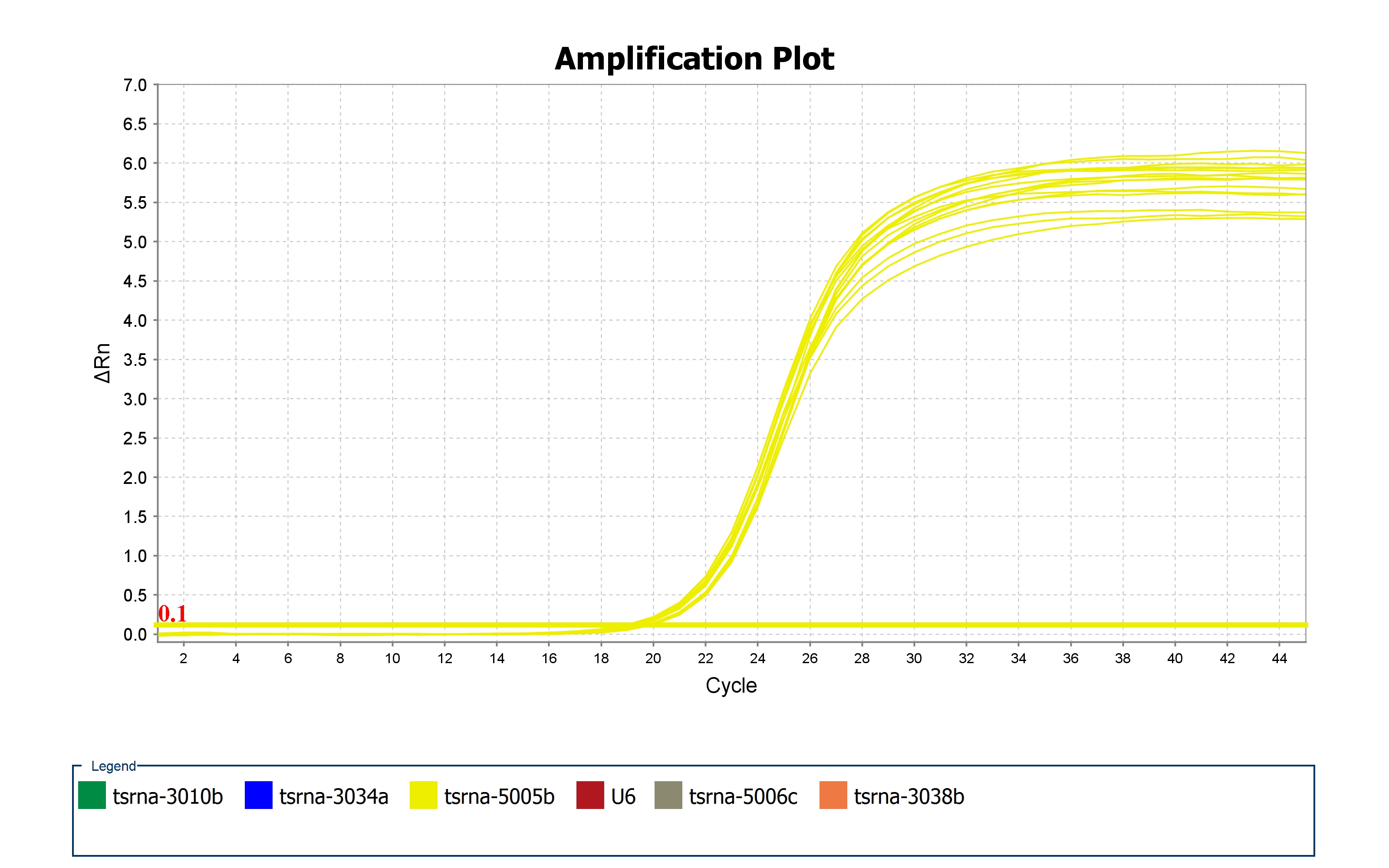

Supplement: Supplemental Information 2 [file peerj-10-14307-s002.zip › Raw data/Figure 4A RT-qPCR/Raw data/Amplification Plot tsrna-5005b.jpg]

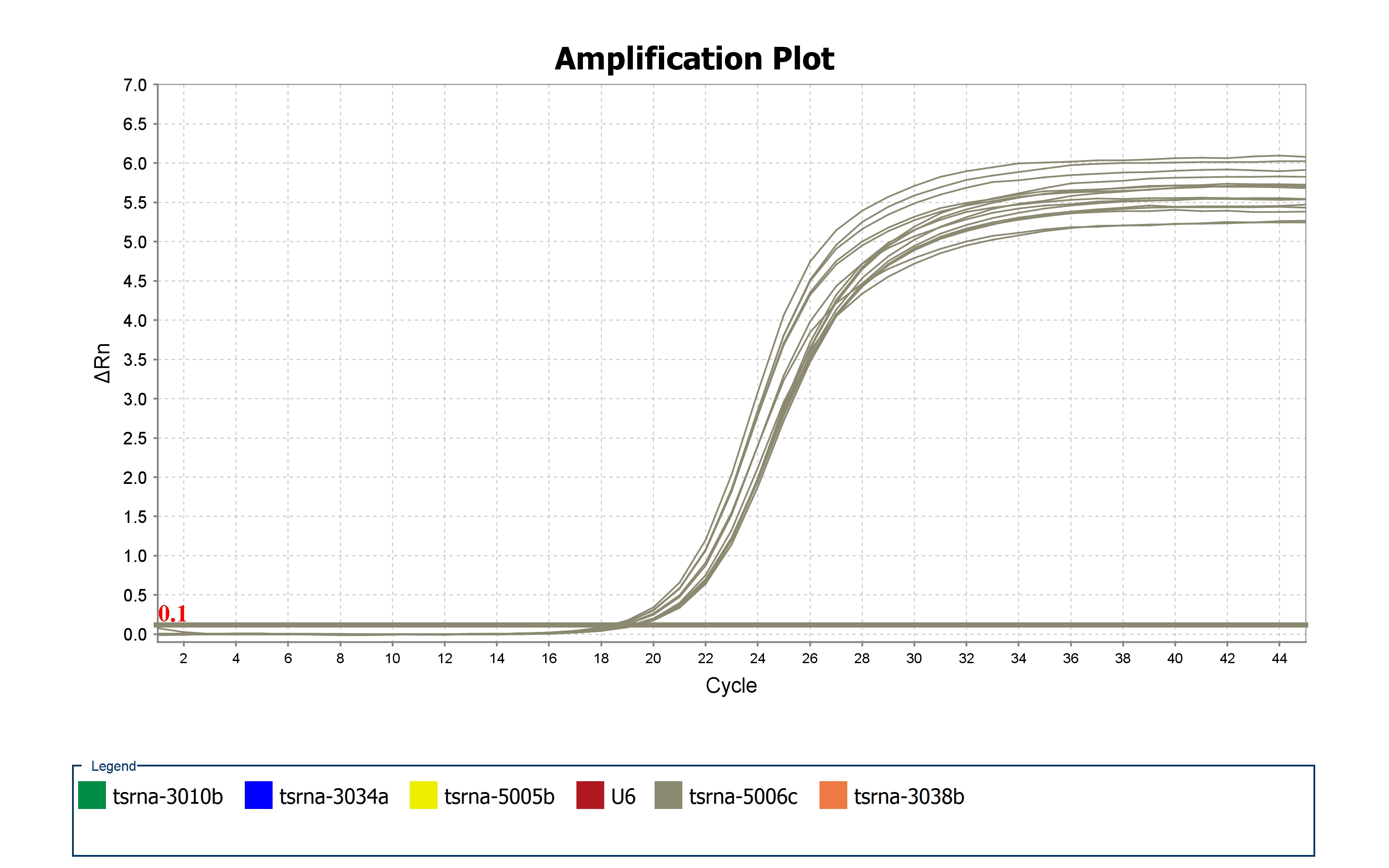

Supplement: Supplemental Information 2 [file peerj-10-14307-s002.zip › Raw data/Figure 4A RT-qPCR/Raw data/Amplification Plot tsrna-5006c.jpg]

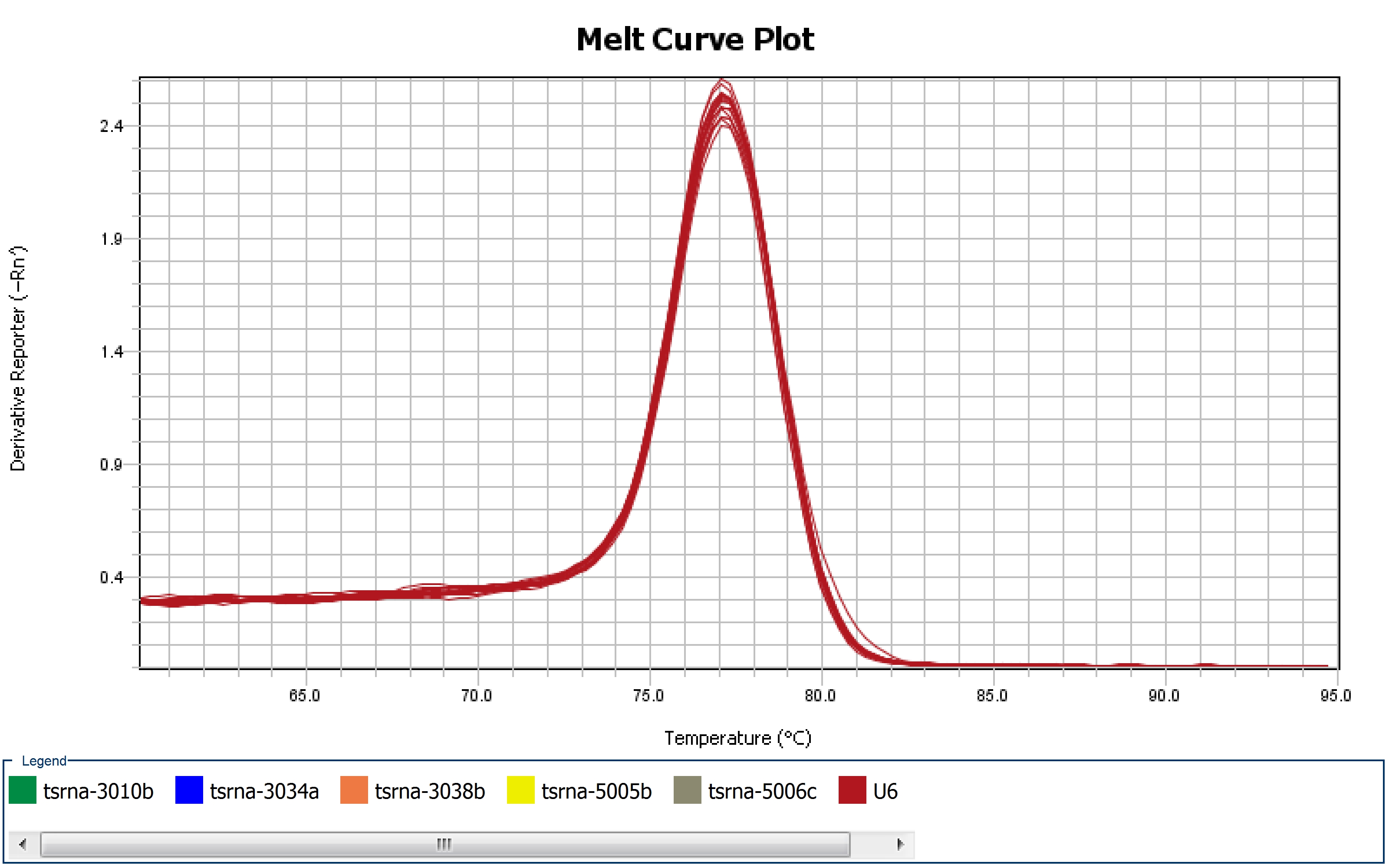

Supplement: Supplemental Information 2 [file peerj-10-14307-s002.zip › Raw data/Figure 4A RT-qPCR/Raw data/Melt Curve Plot U6.jpg]

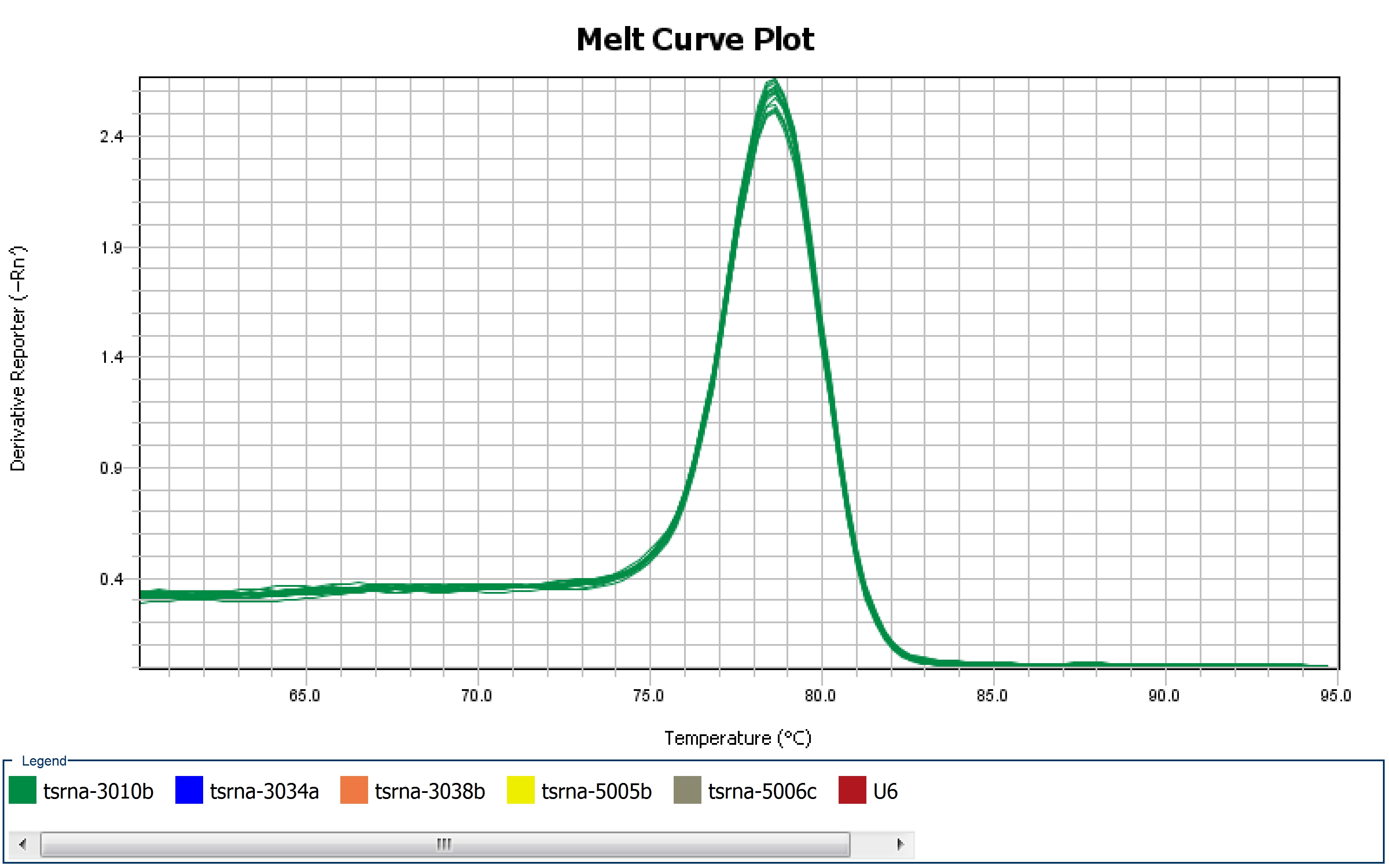

Supplement: Supplemental Information 2 [file peerj-10-14307-s002.zip › Raw data/Figure 4A RT-qPCR/Raw data/Melt Curve Plot tsrna-3010b.jpg]

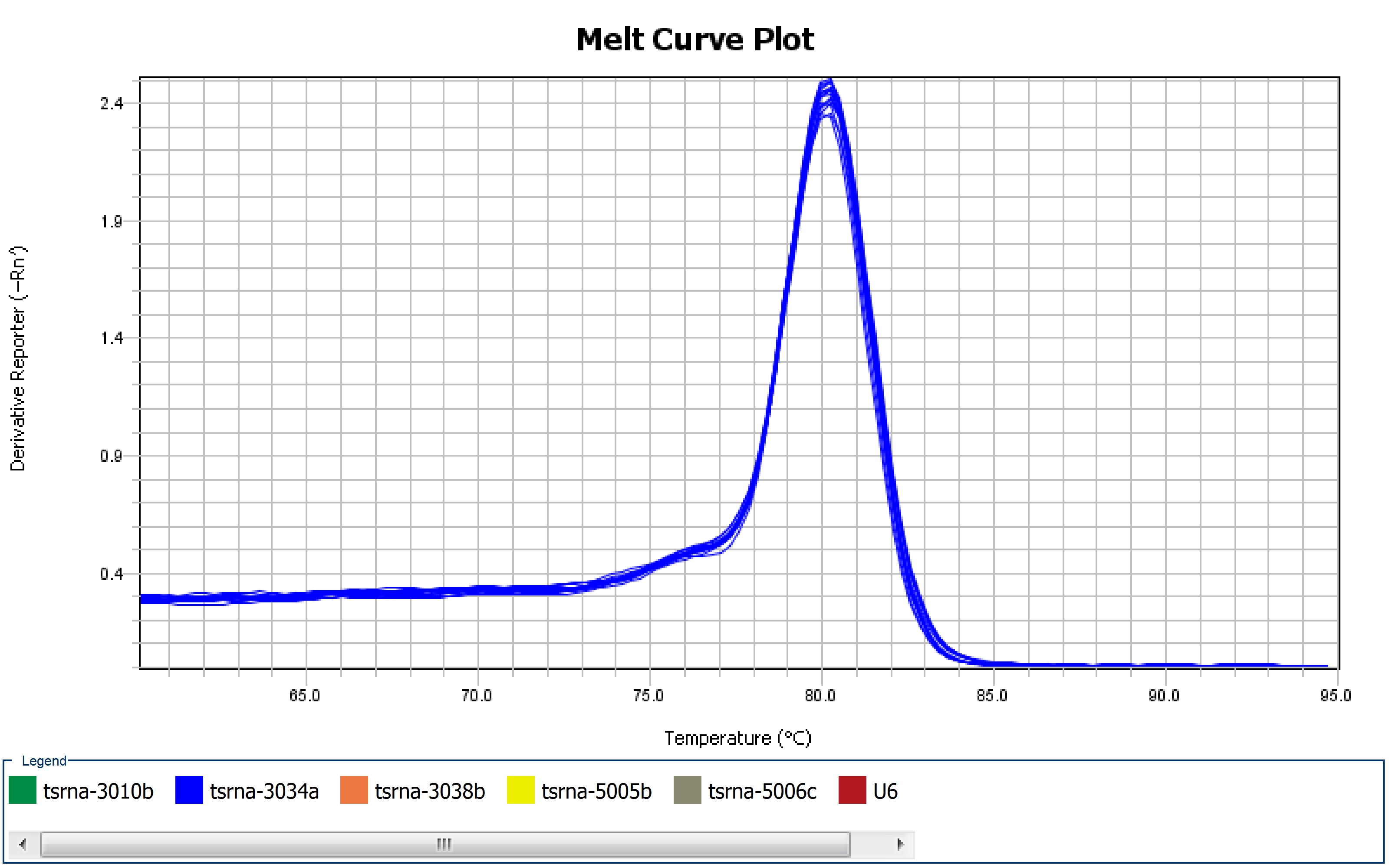

Supplement: Supplemental Information 2 [file peerj-10-14307-s002.zip › Raw data/Figure 4A RT-qPCR/Raw data/Melt Curve Plot tsrna-3034a.jpg]

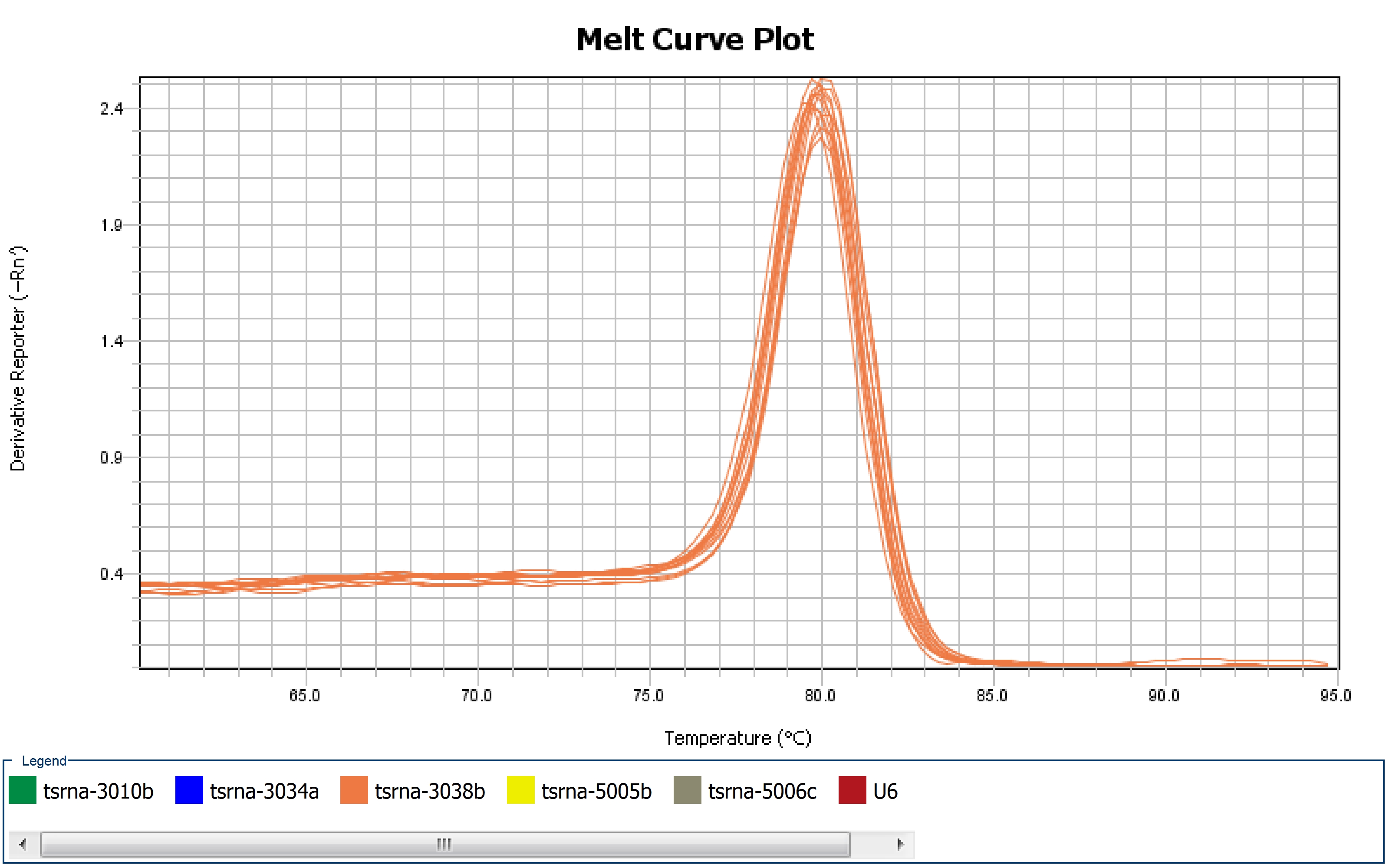

Supplement: Supplemental Information 2 [file peerj-10-14307-s002.zip › Raw data/Figure 4A RT-qPCR/Raw data/Melt Curve Plot tsrna-3038b.jpg]

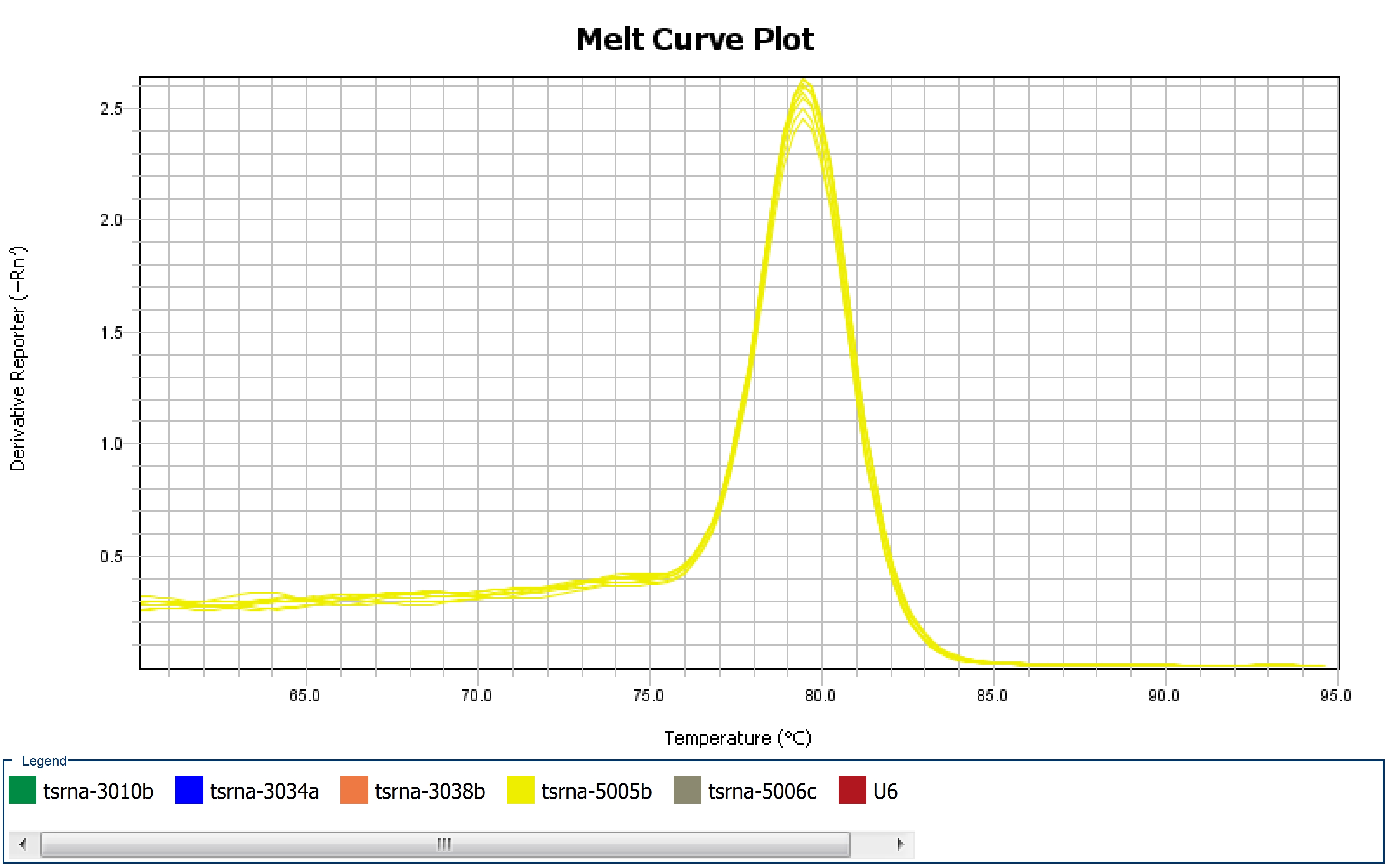

Supplement: Supplemental Information 2 [file peerj-10-14307-s002.zip › Raw data/Figure 4A RT-qPCR/Raw data/Melt Curve Plot tsrna-5005b.jpg]

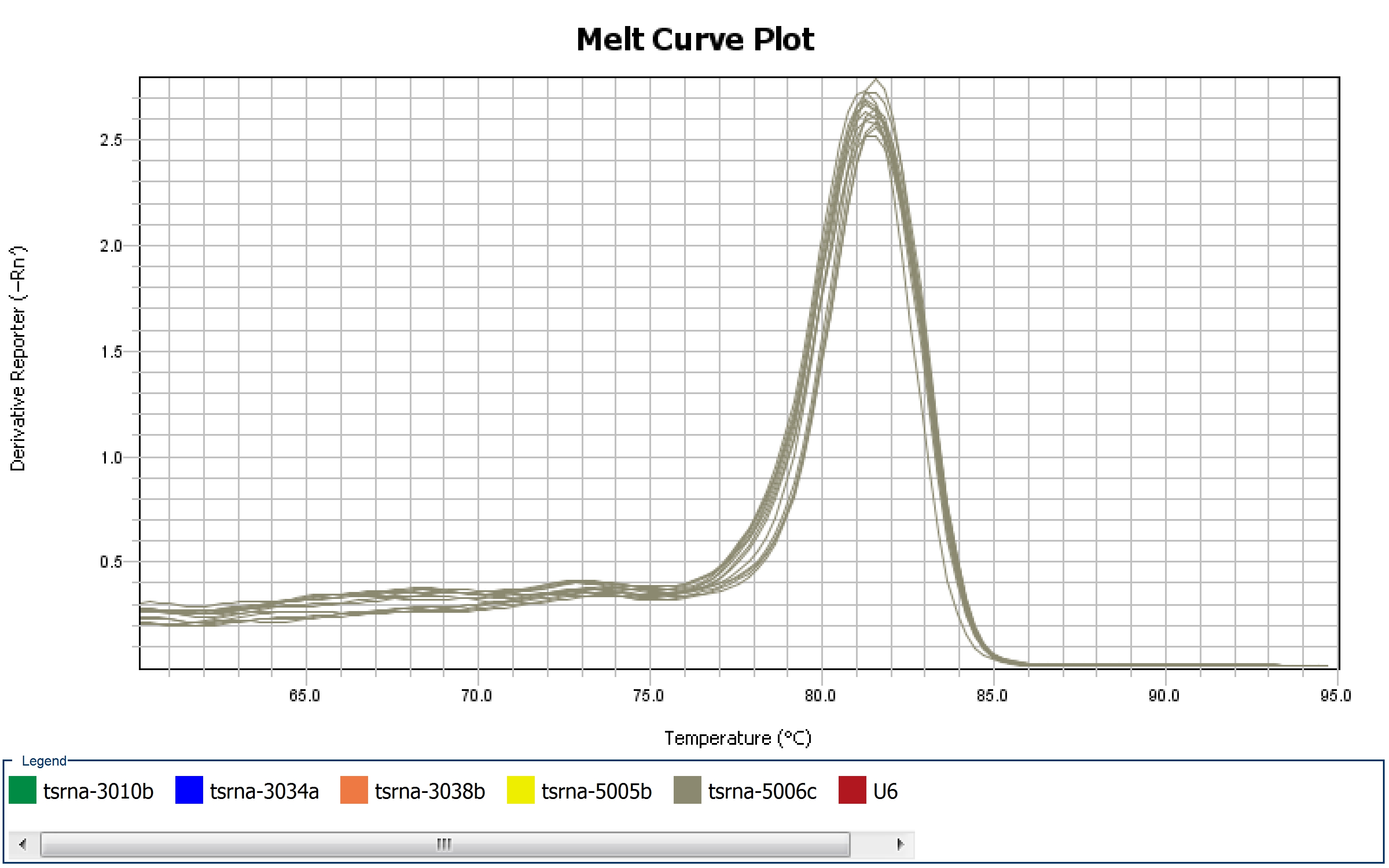

Supplement: Supplemental Information 2 [file peerj-10-14307-s002.zip › Raw data/Figure 4A RT-qPCR/Raw data/Melt Curve Plot tsrna-5006c.jpg]

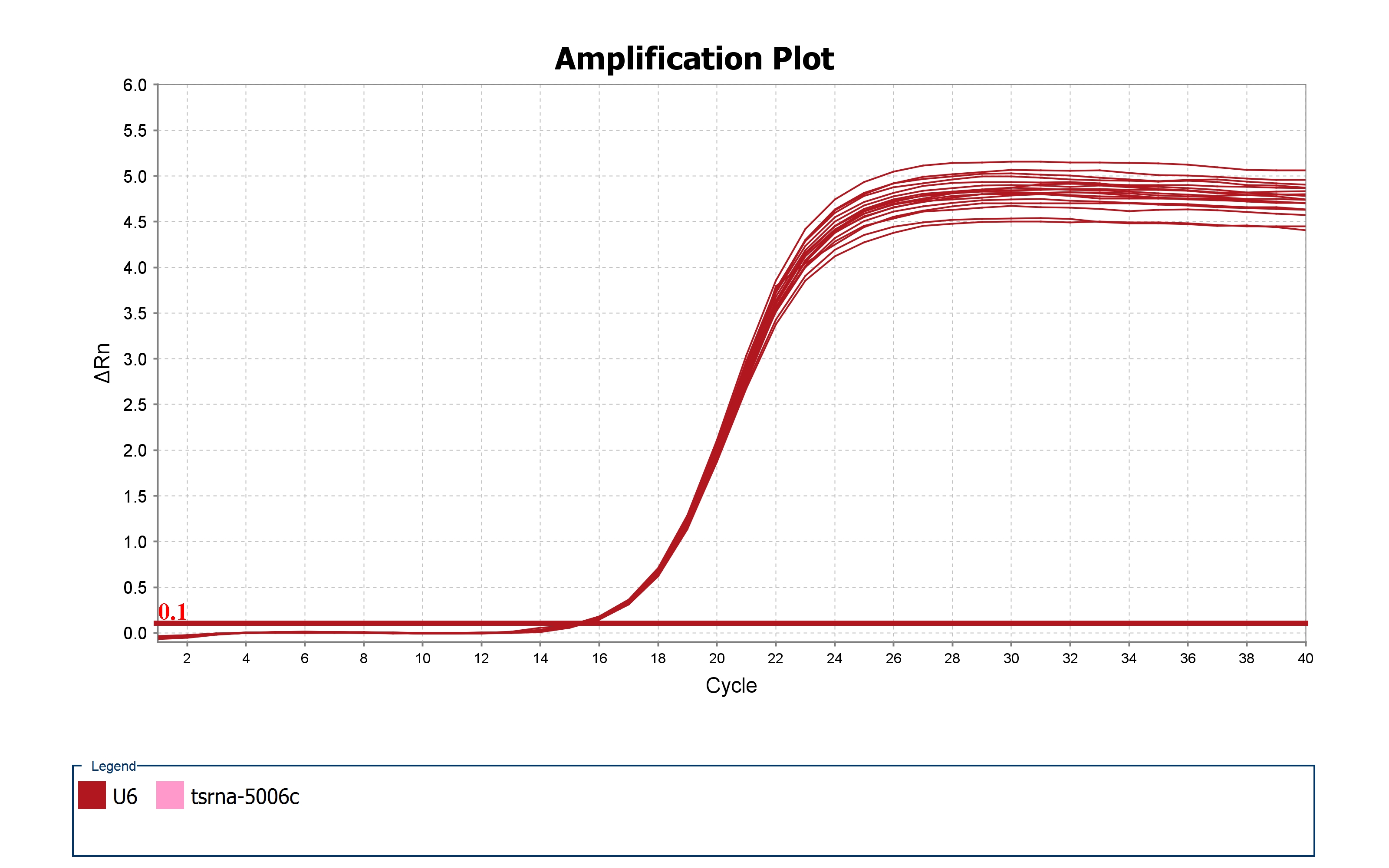

Supplement: Supplemental Information 2 [file peerj-10-14307-s002.zip › Raw data/Figure 4C RT-qPCR/Raw data/Amplification Plot U6.jpg]

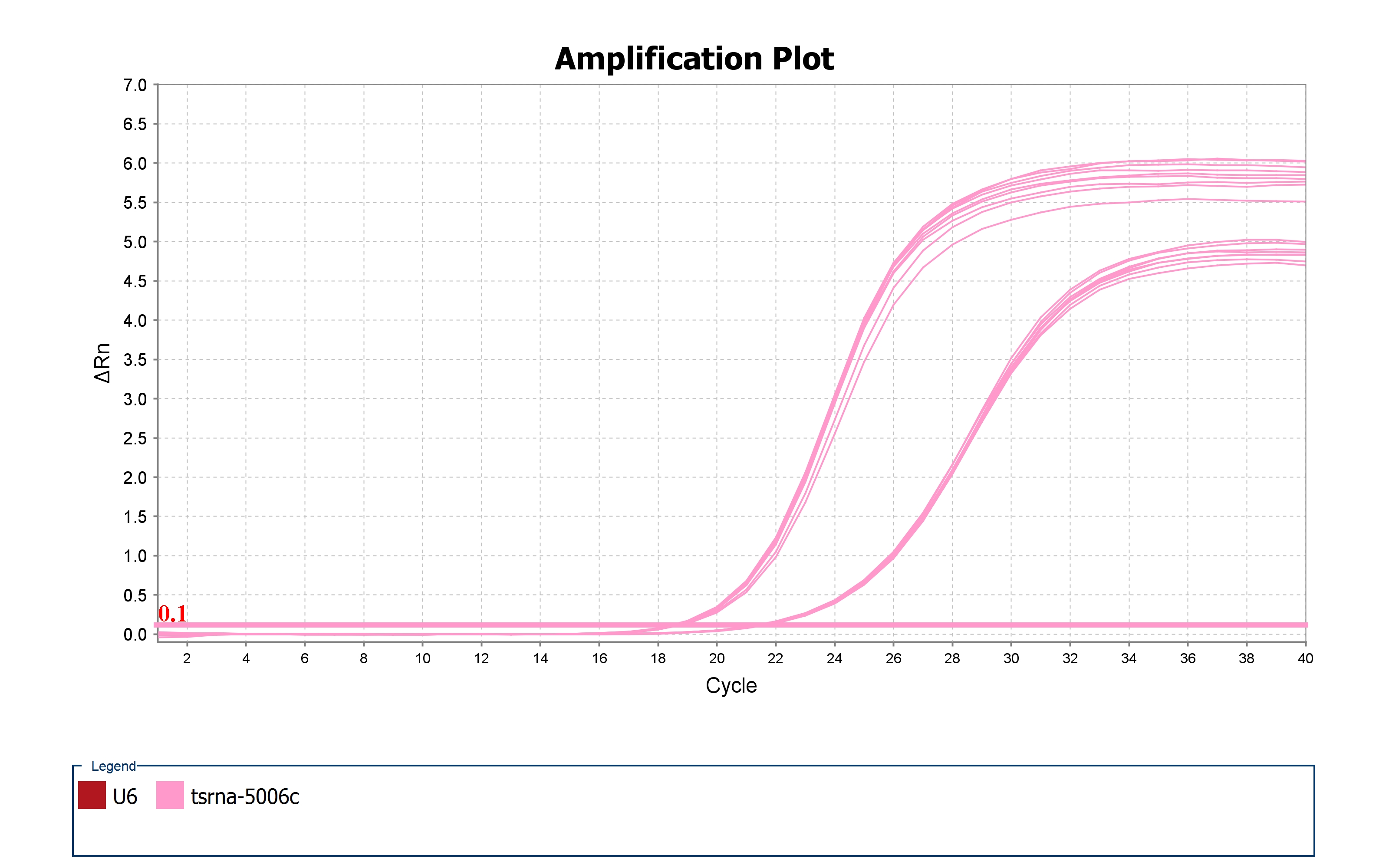

Supplement: Supplemental Information 2 [file peerj-10-14307-s002.zip › Raw data/Figure 4C RT-qPCR/Raw data/Amplification Plot tsrna-5006c.jpg]

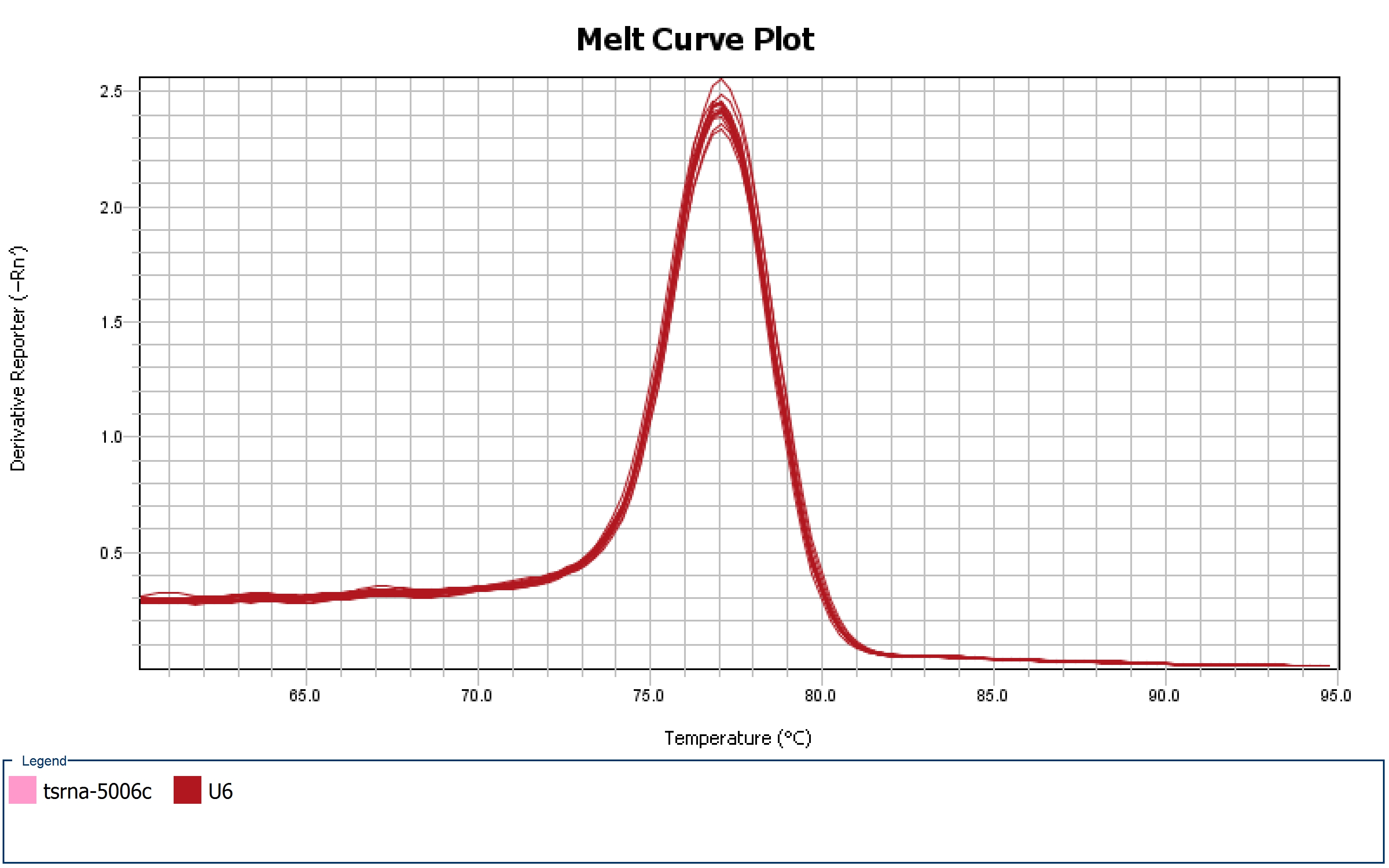

Supplement: Supplemental Information 2 [file peerj-10-14307-s002.zip › Raw data/Figure 4C RT-qPCR/Raw data/Melt Curve Plot U6.jpg]

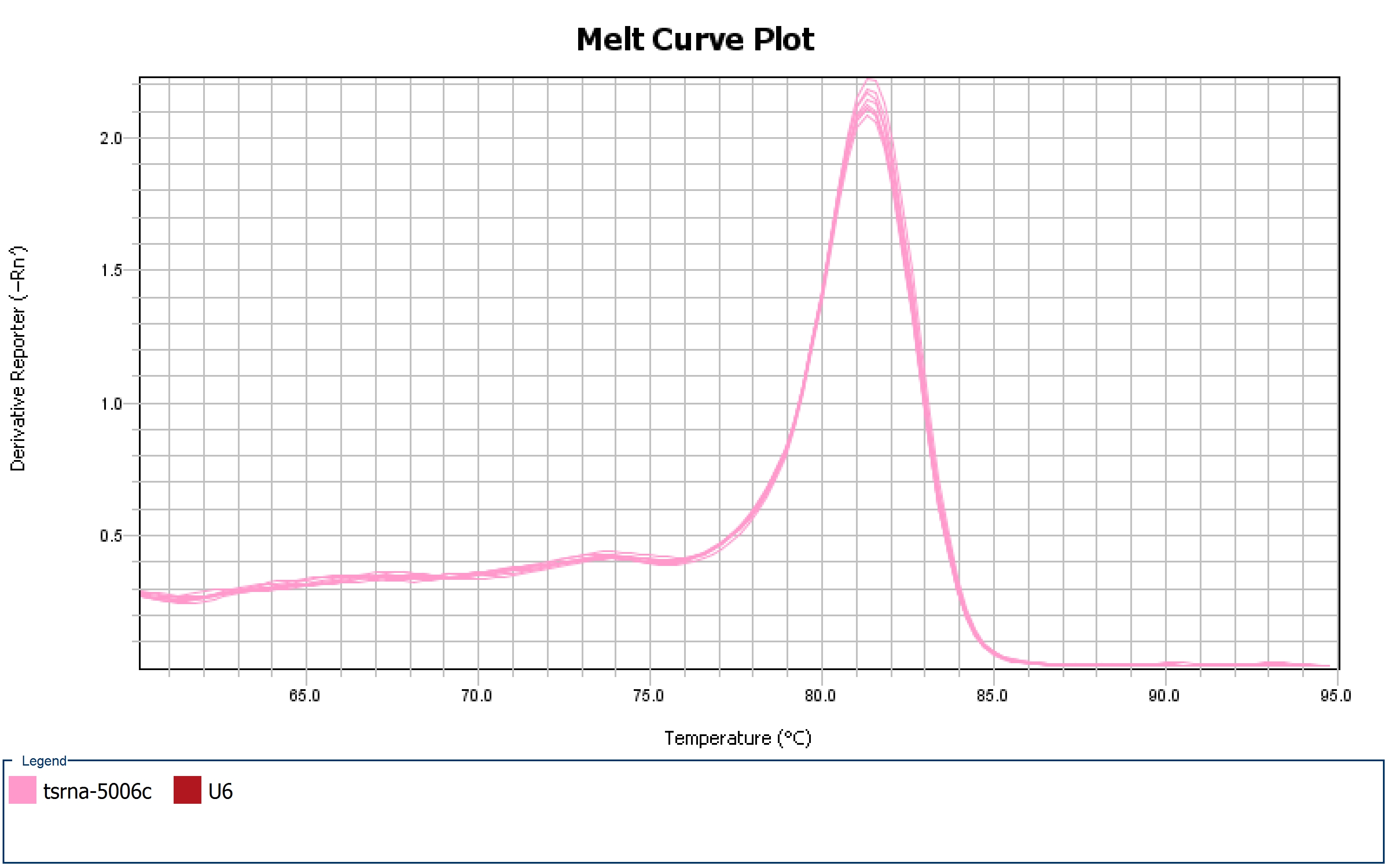

Supplement: Supplemental Information 2 [file peerj-10-14307-s002.zip › Raw data/Figure 4C RT-qPCR/Raw data/Melt Curve Plot tsrna-5006c.jpg]

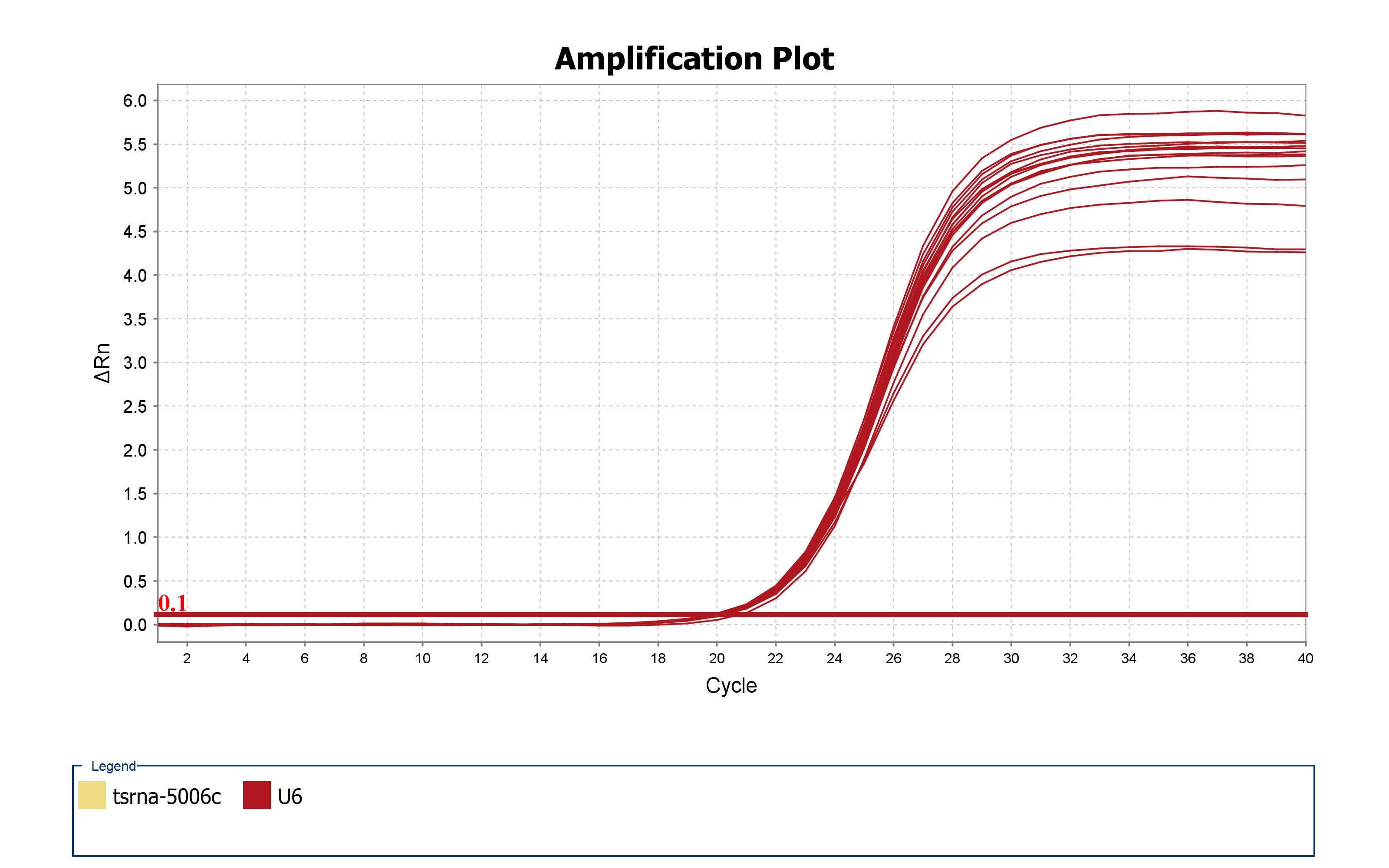

Supplement: Supplemental Information 2 [file peerj-10-14307-s002.zip › Raw data/Figure 4D RT-qPCR/Raw data/Amplification Plot U6.jpg]

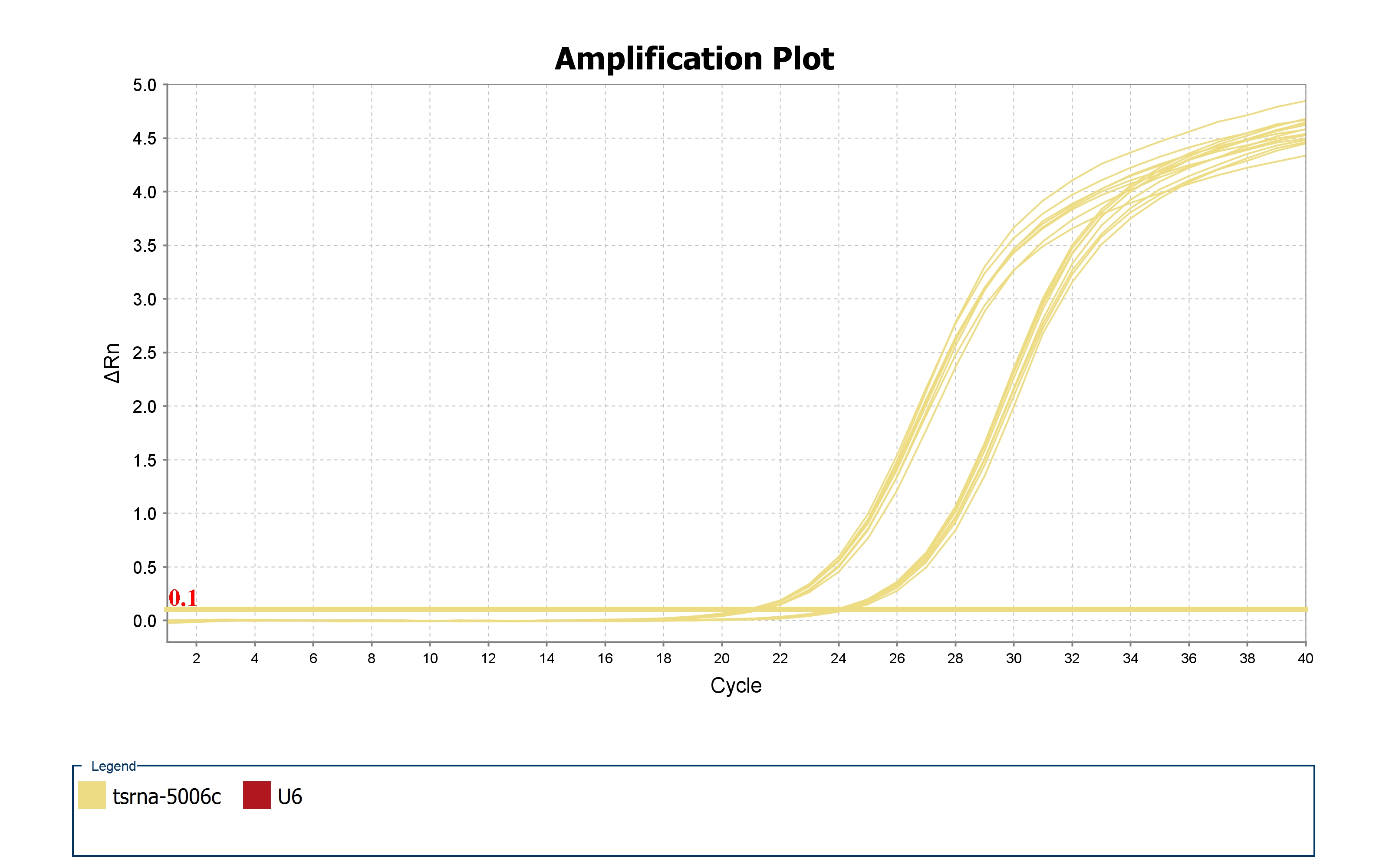

Supplement: Supplemental Information 2 [file peerj-10-14307-s002.zip › Raw data/Figure 4D RT-qPCR/Raw data/Amplification Plot tsrna-5006c.jpg]

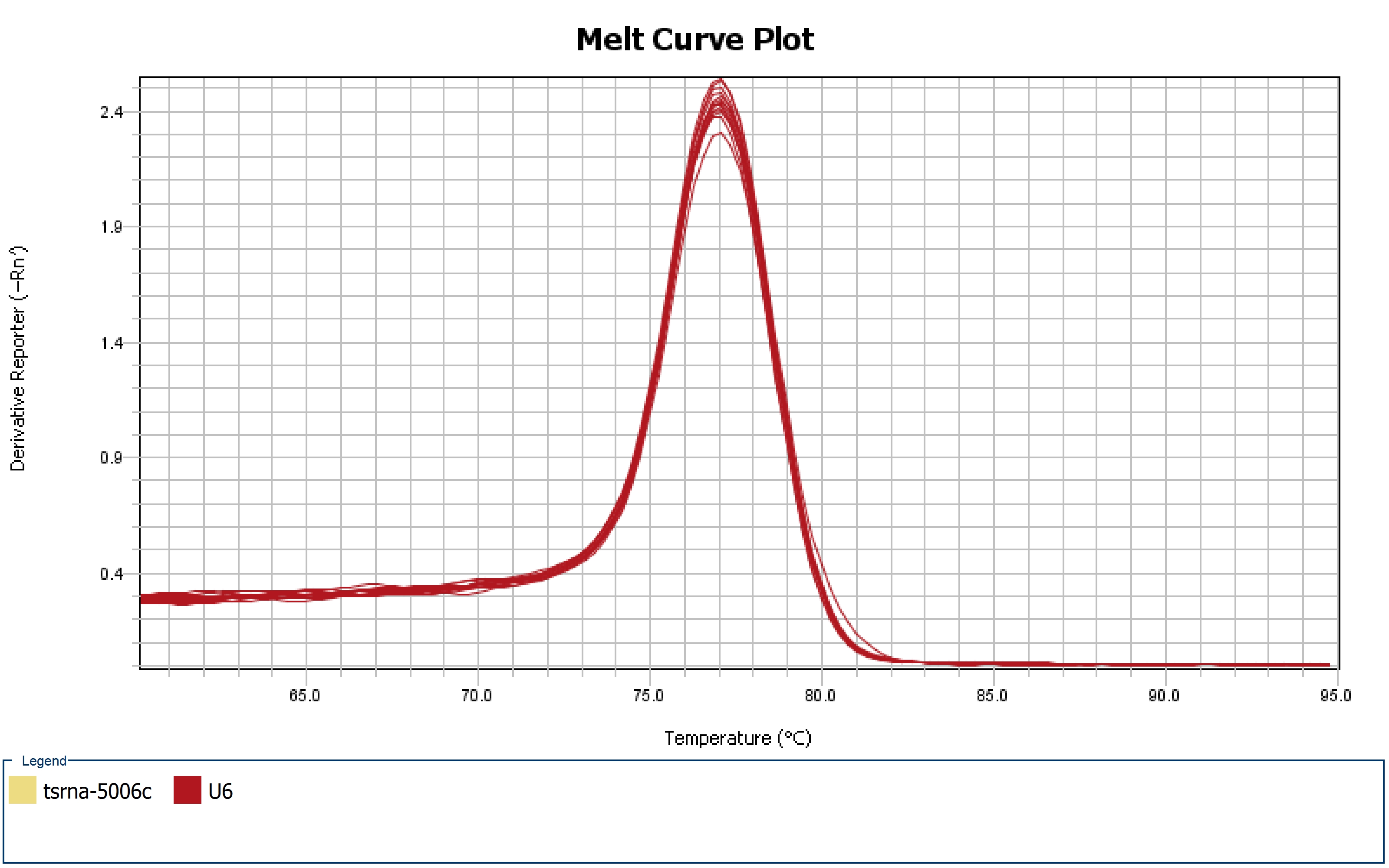

Supplement: Supplemental Information 2 [file peerj-10-14307-s002.zip › Raw data/Figure 4D RT-qPCR/Raw data/Melt Curve Plot U6.jpg]

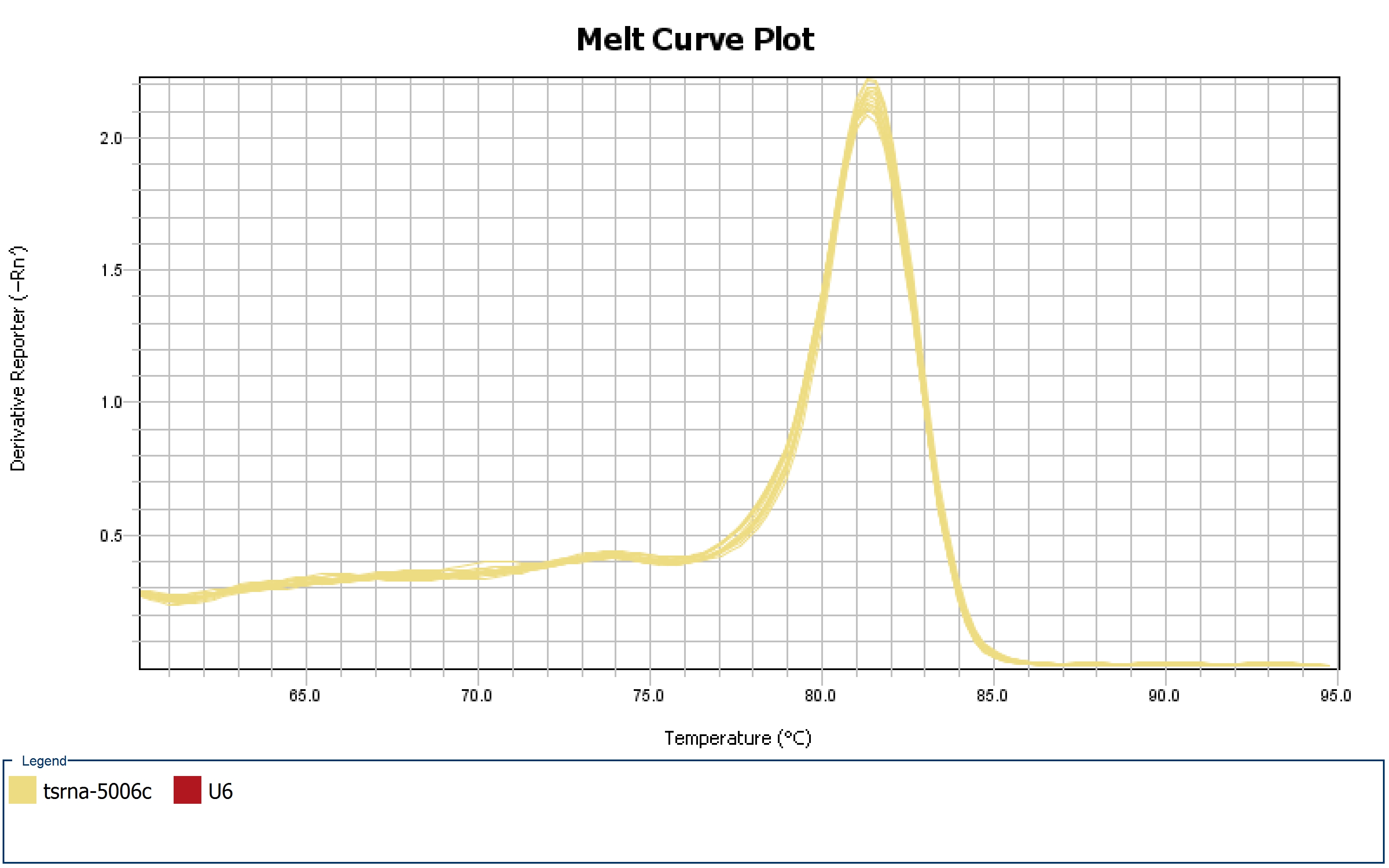

Supplement: Supplemental Information 2 [file peerj-10-14307-s002.zip › Raw data/Figure 4D RT-qPCR/Raw data/Melt Curve Plot tsrna-5006c.jpg]

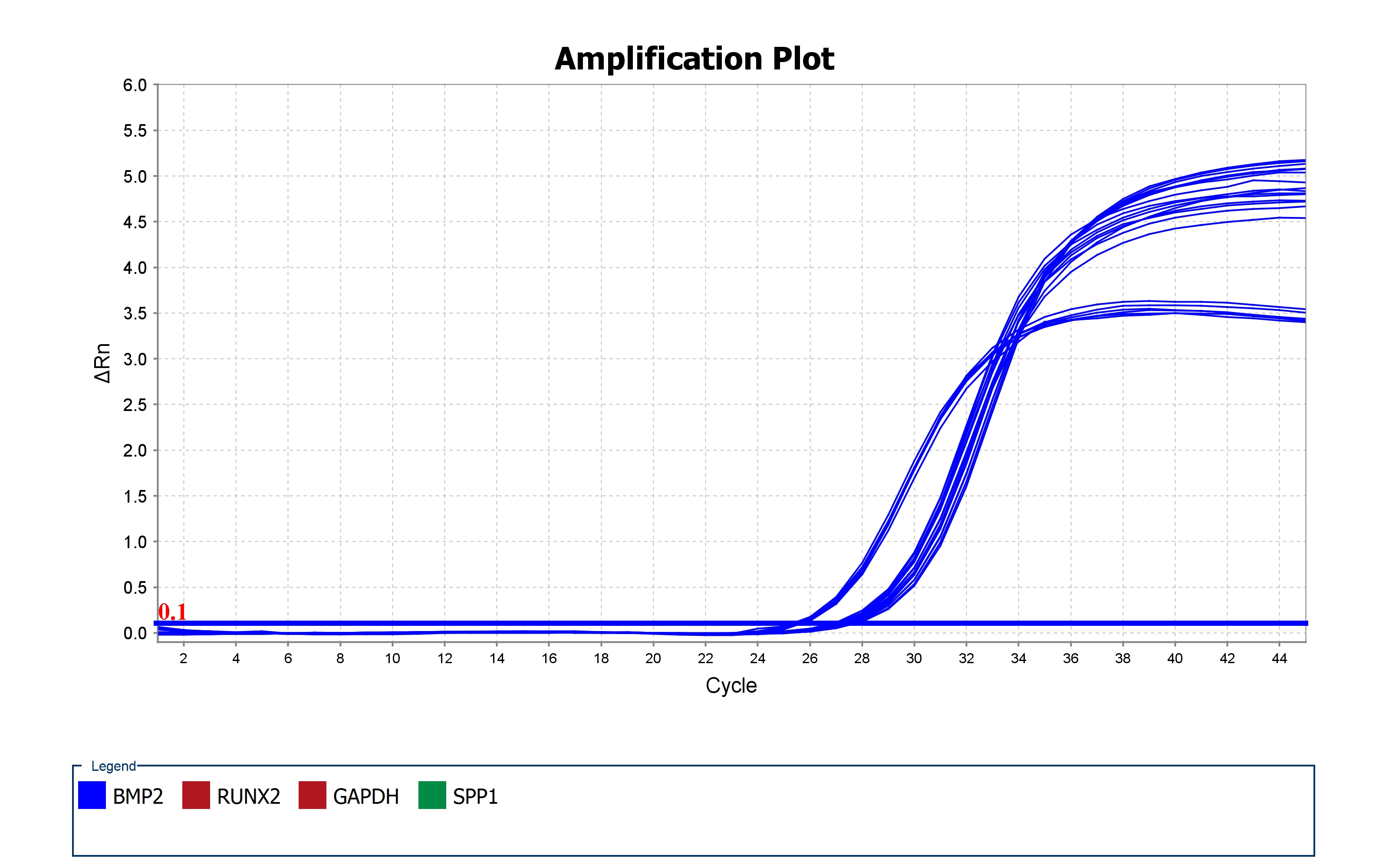

Supplement: Supplemental Information 2 [file peerj-10-14307-s002.zip › Raw data/Figure 5B RT-qPCR/Raw data/Amplification Plot BMP2.jpg]

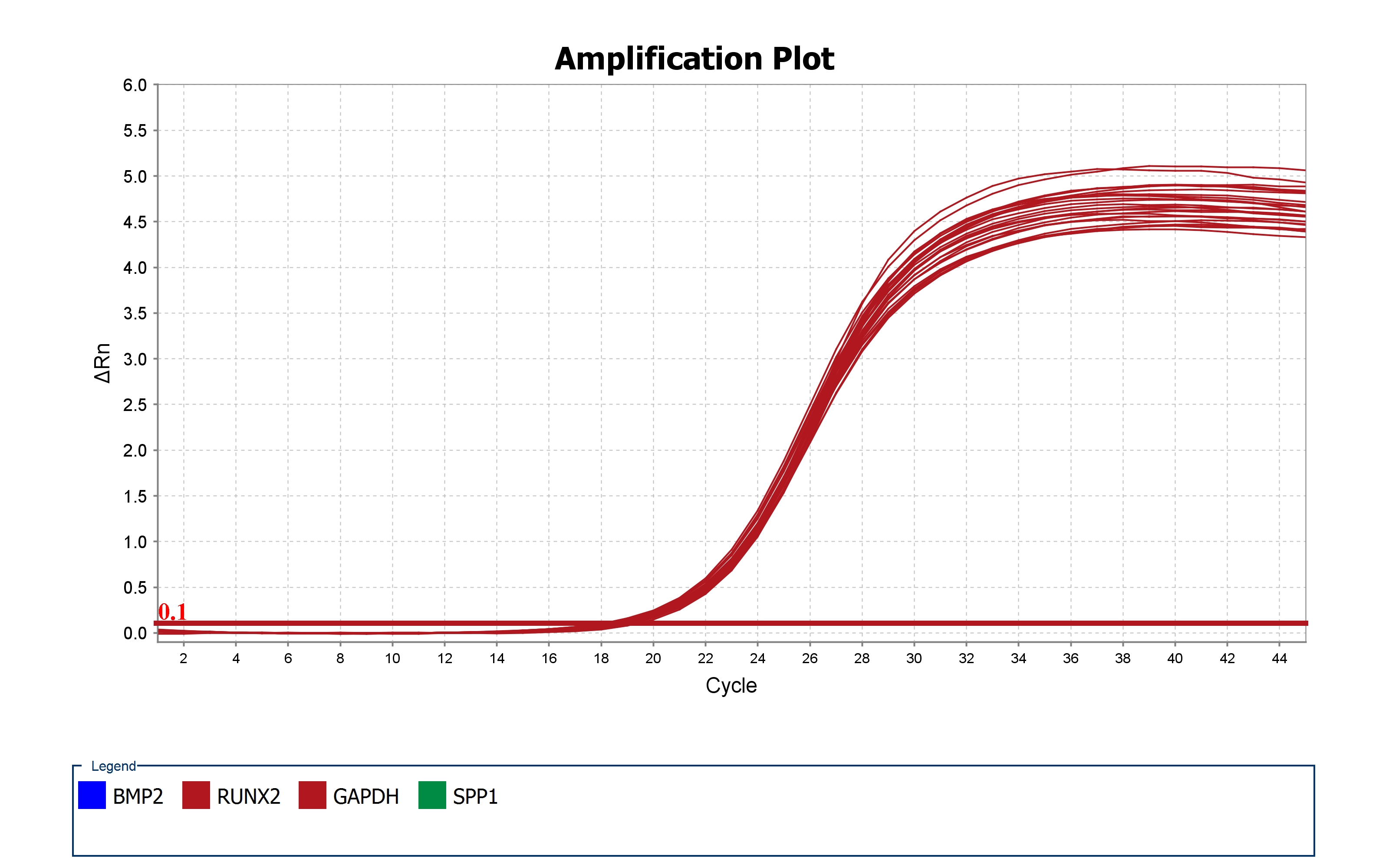

Supplement: Supplemental Information 2 [file peerj-10-14307-s002.zip › Raw data/Figure 5B RT-qPCR/Raw data/Amplification Plot GAPDH.jpg]

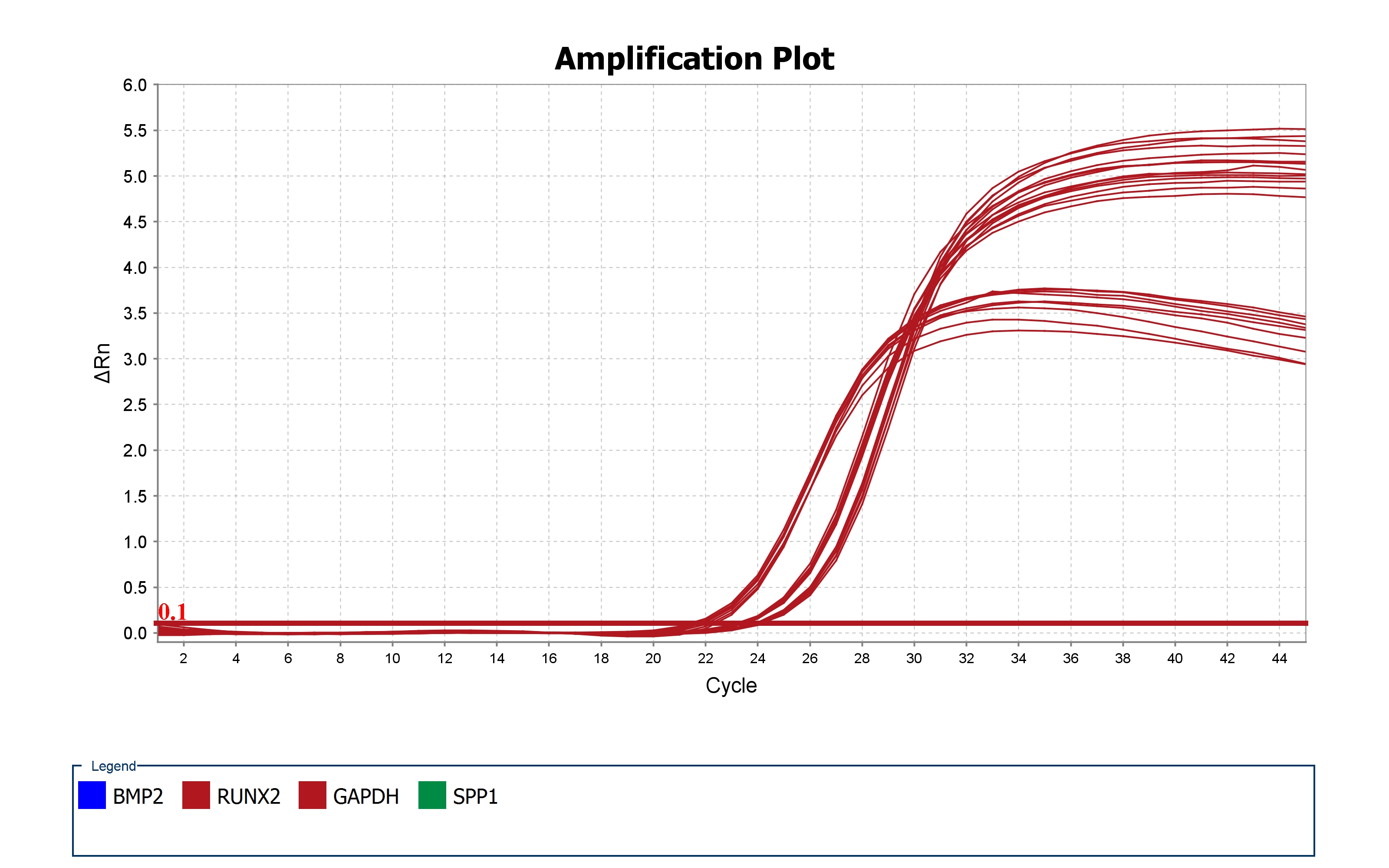

Supplement: Supplemental Information 2 [file peerj-10-14307-s002.zip › Raw data/Figure 5B RT-qPCR/Raw data/Amplification Plot RUNX2.jpg]

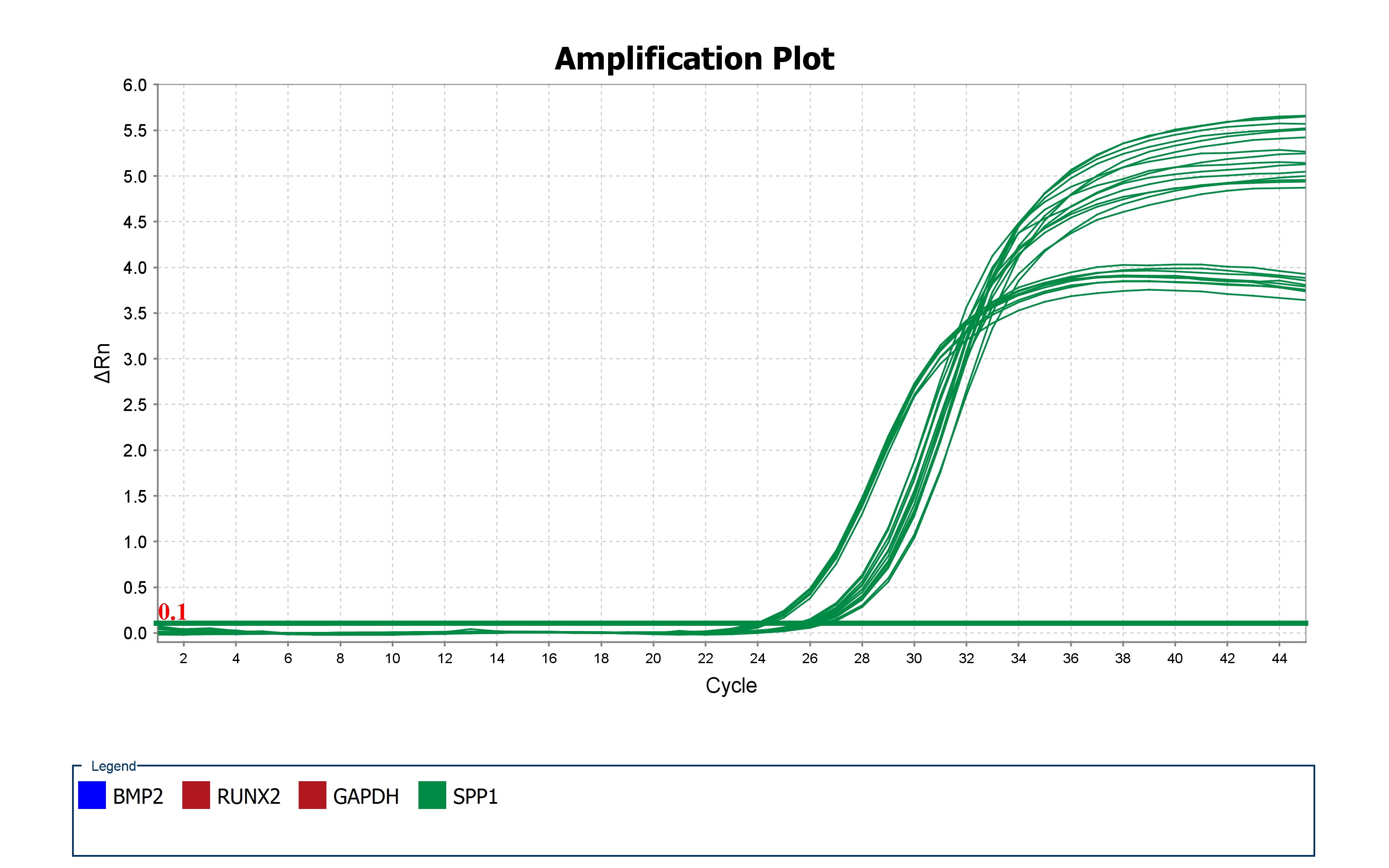

Supplement: Supplemental Information 2 [file peerj-10-14307-s002.zip › Raw data/Figure 5B RT-qPCR/Raw data/Amplification Plot SPP1.jpg]

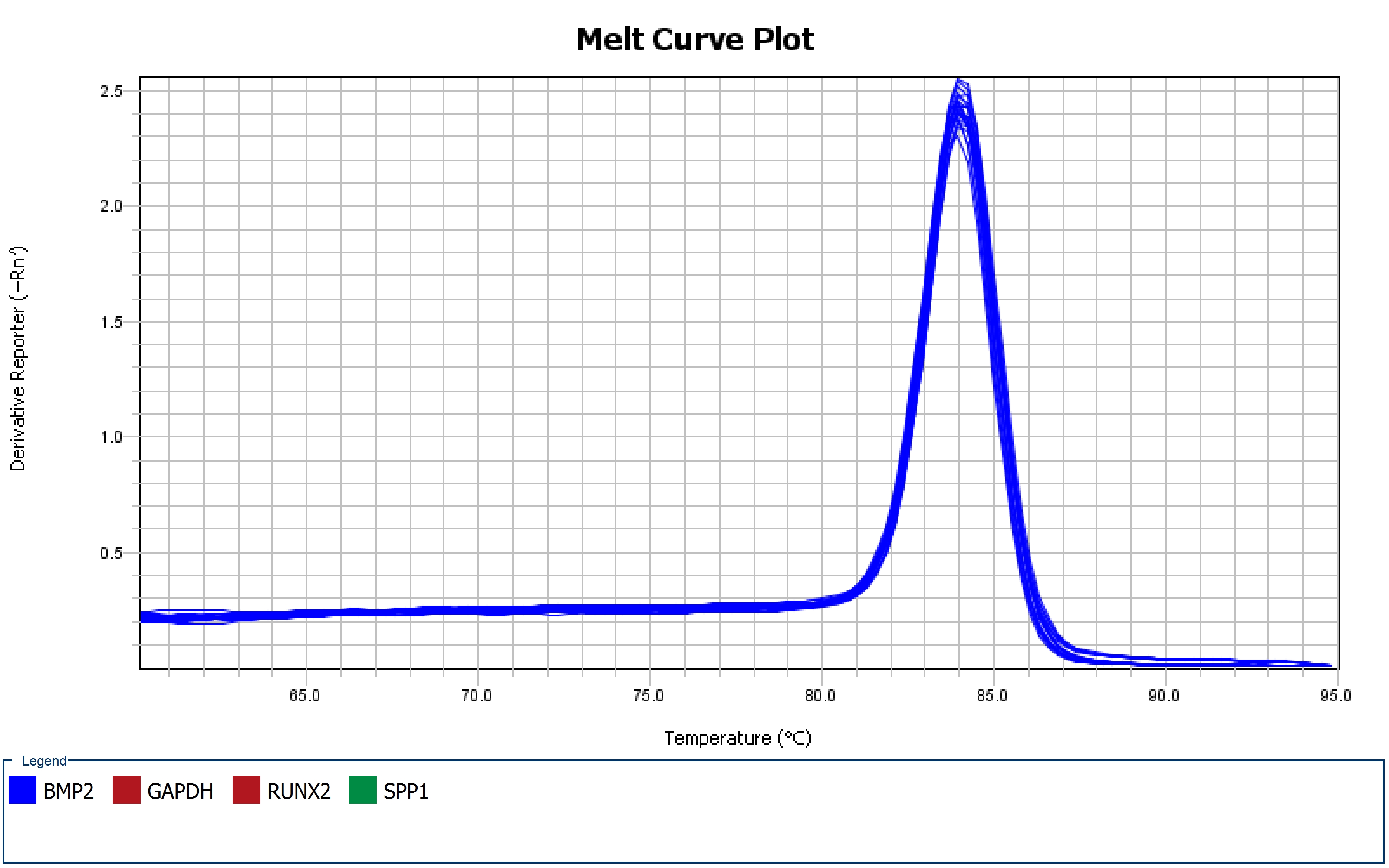

Supplement: Supplemental Information 2 [file peerj-10-14307-s002.zip › Raw data/Figure 5B RT-qPCR/Raw data/Melt Curve Plot BMP2.jpg]

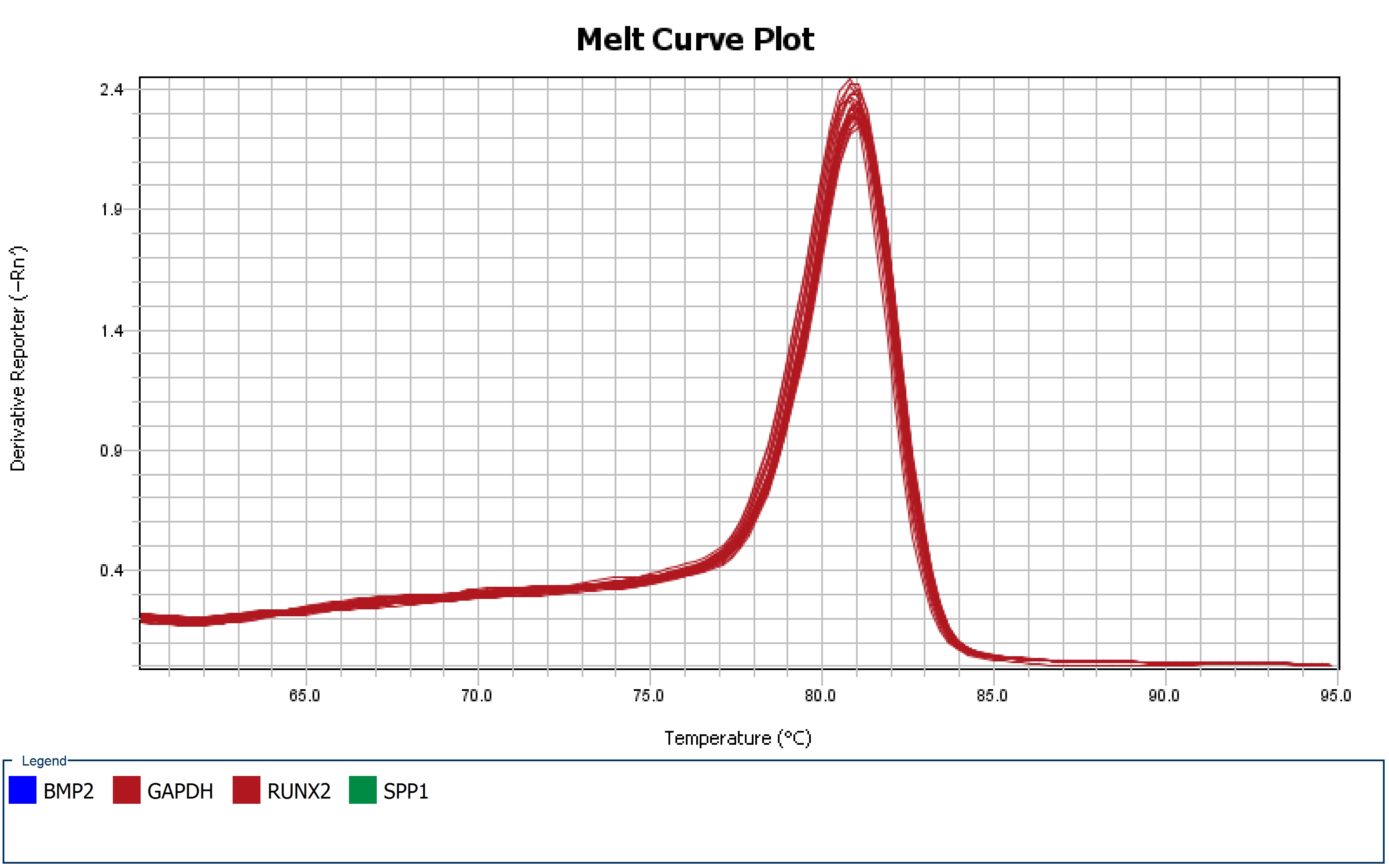

Supplement: Supplemental Information 2 [file peerj-10-14307-s002.zip › Raw data/Figure 5B RT-qPCR/Raw data/Melt Curve Plot GAPDH.jpg]

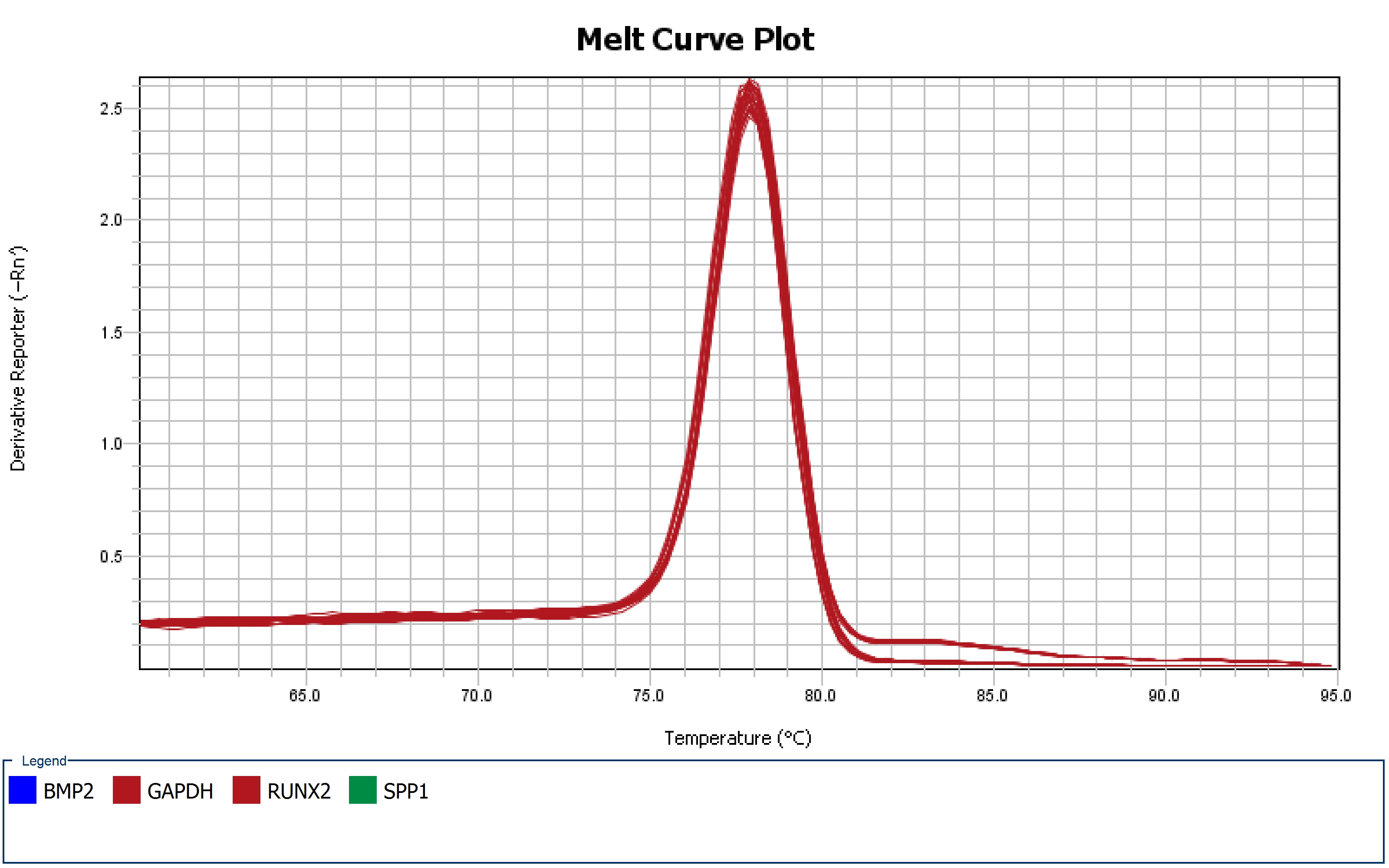

Supplement: Supplemental Information 2 [file peerj-10-14307-s002.zip › Raw data/Figure 5B RT-qPCR/Raw data/Melt Curve Plot RUNX2.jpg]

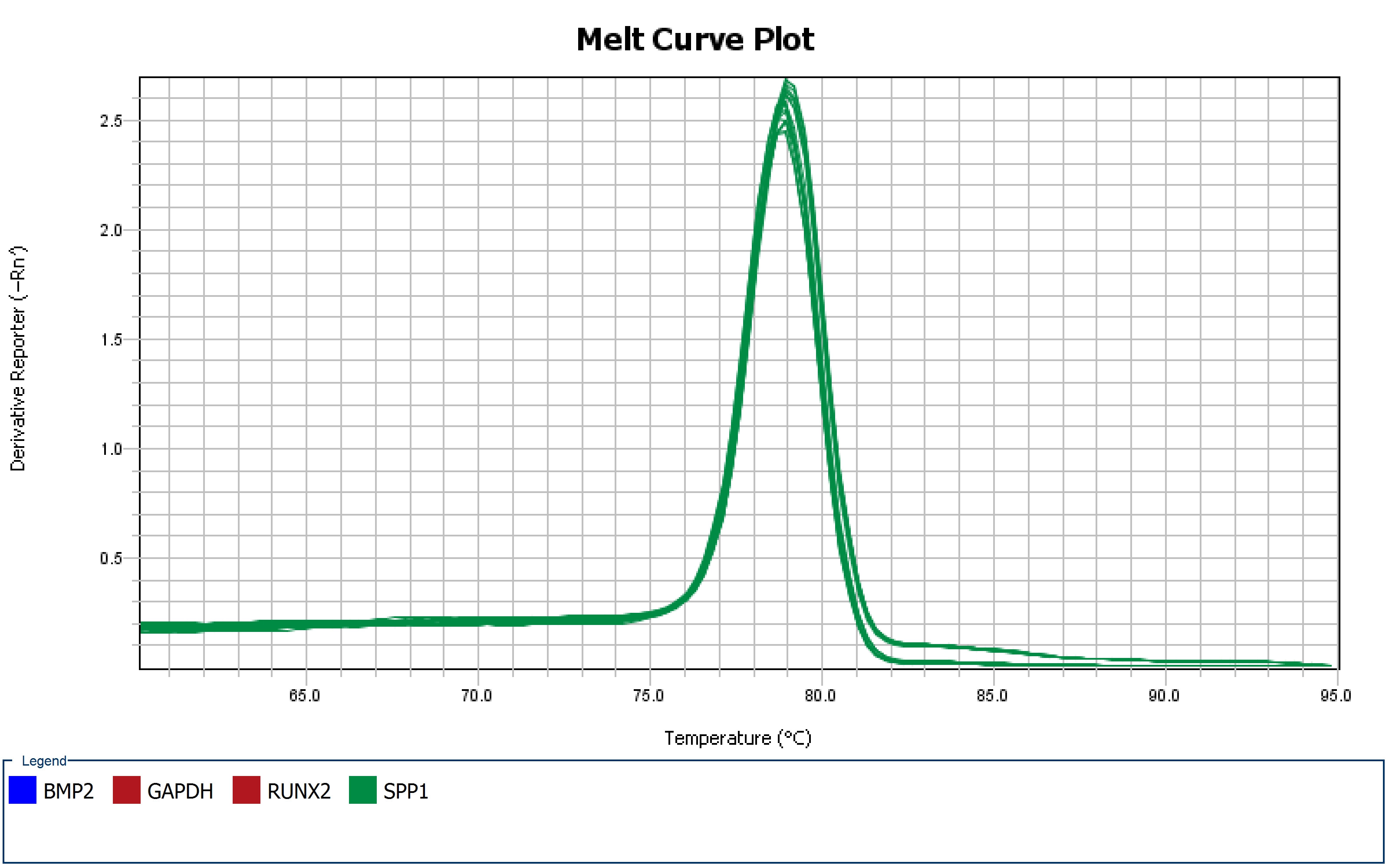

Supplement: Supplemental Information 2 [file peerj-10-14307-s002.zip › Raw data/Figure 5B RT-qPCR/Raw data/Melt Curve Plot SPP1.jpg]

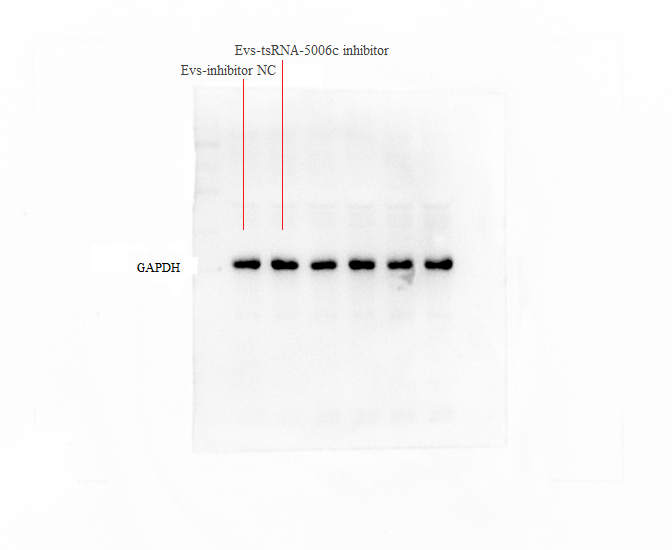

Supplement: Supplemental Information 2 [file peerj-10-14307-s002.zip › Raw data/Figure 5C WB/GAPDH.tif]

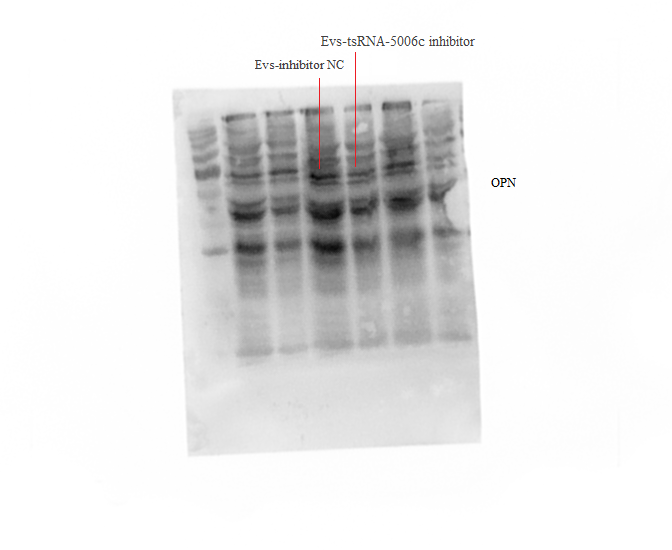

Supplement: Supplemental Information 2 [file peerj-10-14307-s002.zip › Raw data/Figure 5C WB/OPN.tif]

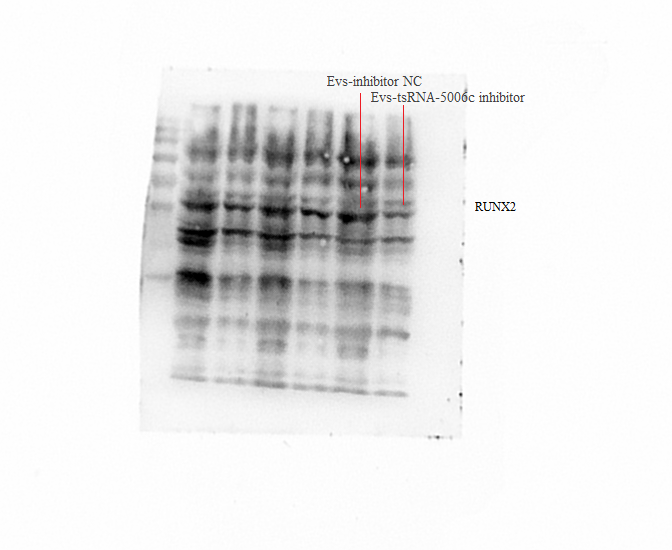

Supplement: Supplemental Information 2 [file peerj-10-14307-s002.zip › Raw data/Figure 5C WB/RUNX2.tif]

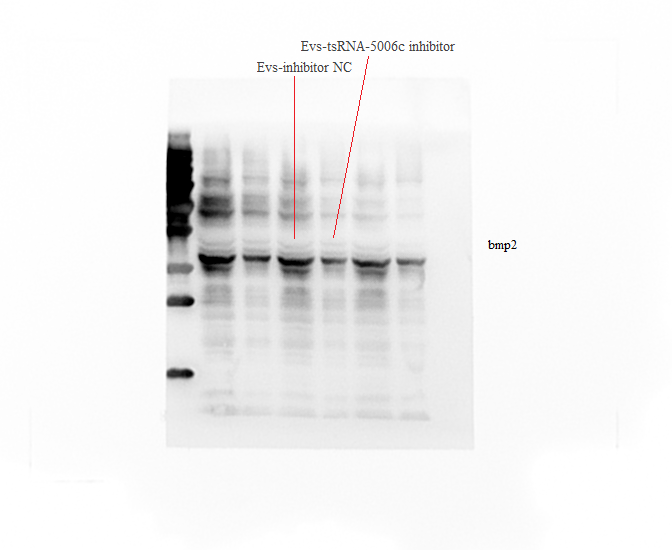

Supplement: Supplemental Information 2 [file peerj-10-14307-s002.zip › Raw data/Figure 5C WB/bmp2.tif]

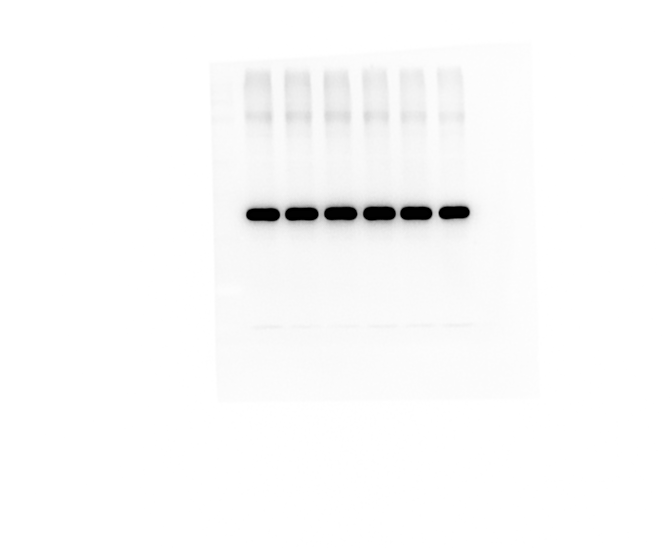

Supplement: Supplemental Information 2 [file peerj-10-14307-s002.zip › Raw data/Figure 5D WB/GAPDH.tif]

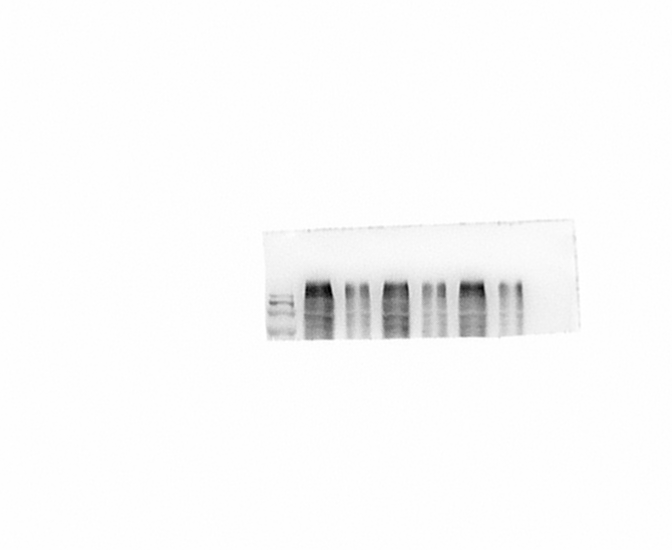

Supplement: Supplemental Information 2 [file peerj-10-14307-s002.zip › Raw data/Figure 5D WB/collagen I.tif]

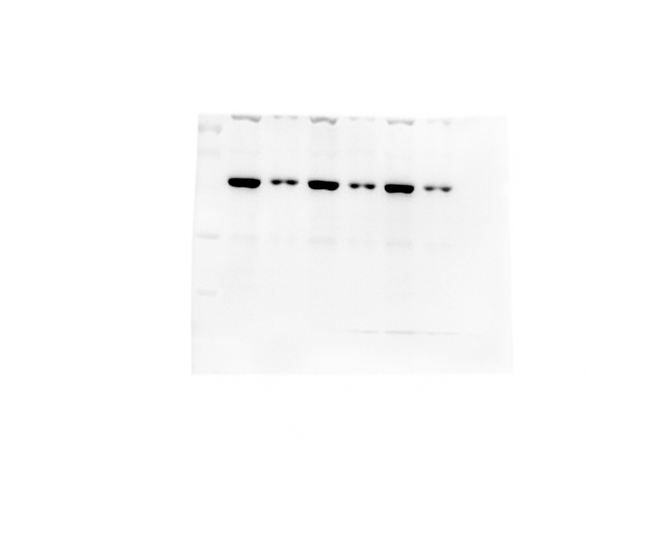

Supplement: Supplemental Information 2 [file peerj-10-14307-s002.zip › Raw data/Figure 5D WB/a┴-SMA.tif]

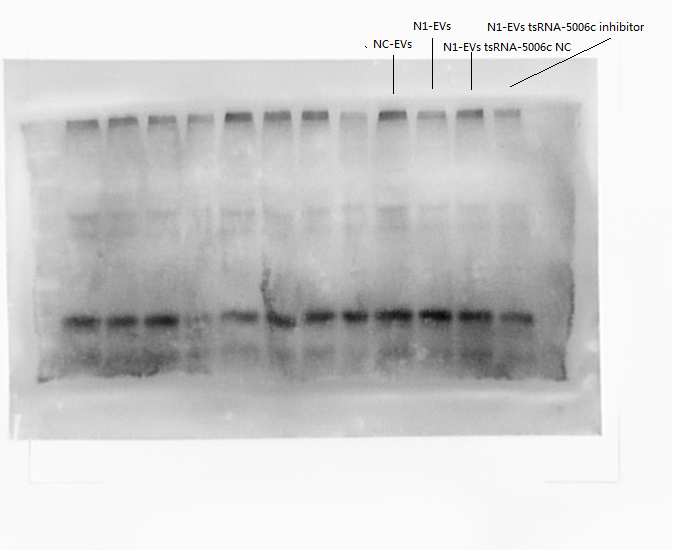

Supplement: Supplemental Information 2 [file peerj-10-14307-s002.zip › Raw data/Figure 6C WB/BNIP3 .tif]

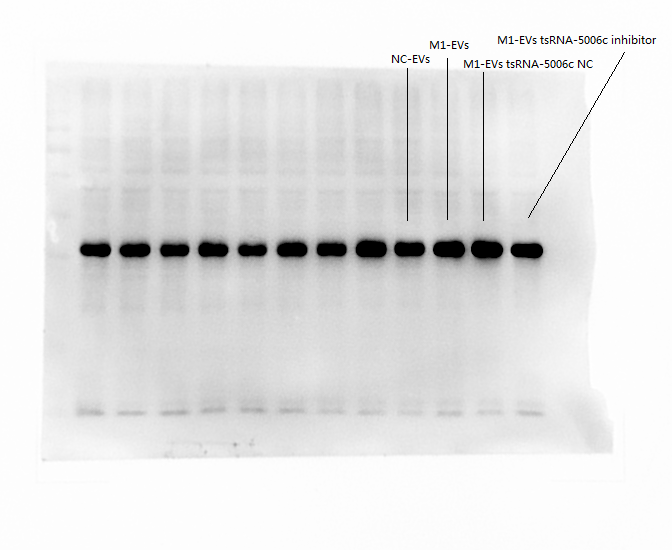

Supplement: Supplemental Information 2 [file peerj-10-14307-s002.zip › Raw data/Figure 6C WB/GAPDH.tif]

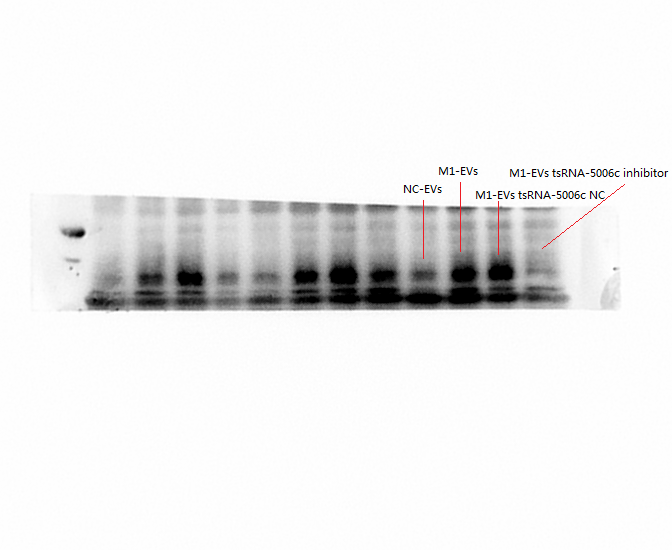

Supplement: Supplemental Information 2 [file peerj-10-14307-s002.zip › Raw data/Figure 6C WB/LC3.tif]

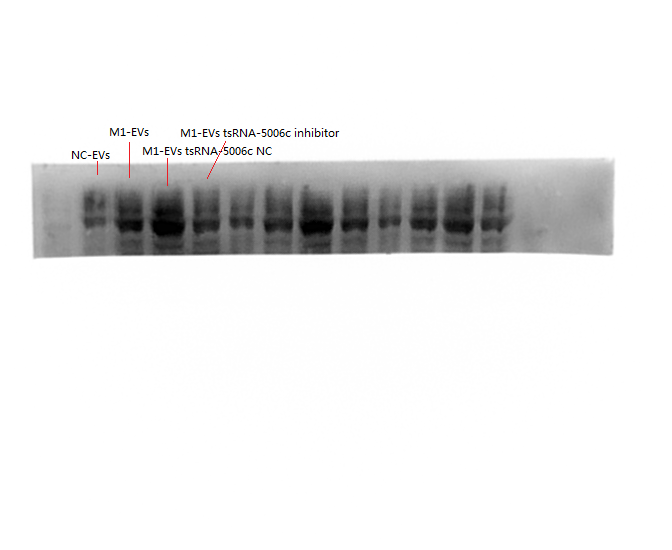

Supplement: Supplemental Information 2 [file peerj-10-14307-s002.zip › Raw data/Figure 6C WB/PGC.tif]
